# Octarepeat region flexibility impacts prion function, endoproteolysis and disease manifestation

**DOI:** 10.15252/emmm.201404588

**Published:** 2015-02-06

**Authors:** Agnes Lau, Alex McDonald, Nathalie Daude, Charles E Mays, Eric D Walter, Robin Aglietti, Robert CC Mercer, Serene Wohlgemuth, Jacques van der Merwe, Jing Yang, Hristina Gapeshina, Chae Kim, Jennifer Grams, Beipei Shi, Holger Wille, Aru Balachandran, Gerold Schmitt-Ulms, Jiri G Safar, Glenn L Millhauser, David Westaway

**Affiliations:** 1 Centre for Prions and Protein Folding Diseases, University of AlbertaEdmonton, AB, Canada; 2 Department of Medicine, University of AlbertaEdmonton, AB, Canada; 3 Department of Chemistry and Biochemistry, University of California Santa CruzSanta Cruz, CA, USA; 4 National Prion Disease Surveillance Center, Departments of Pathology and Neurology, School of Medicine, Case Western Reserve UniversityCleveland, OH, USA; 5 Department of Biochemistry, University of AlbertaEdmonton, AB, Canada; 6 CFIA LabNepean, ON, Canada; 7 Tanz Centre for Research in Neurodegenerative Diseases, Department of Laboratory Medicine and Pathobiology, University of TorontoToronto, ON, Canada

**Keywords:** C2, copper, endoproteolysis, octarepeats, prion

## Abstract

The cellular prion protein (PrP^C^) comprises a natively unstructured N-terminal domain, including a metal-binding octarepeat region (OR) and a linker, followed by a C-terminal domain that misfolds to form PrP^S^^c^ in Creutzfeldt-Jakob disease. PrP^C^ β-endoproteolysis to the C2 fragment allows PrP^S^^c^ formation, while α-endoproteolysis blocks production. To examine the OR, we used structure-directed design to make novel alleles, ‘S1’ and ‘S3’, locking this region in extended or compact conformations, respectively. S1 and S3 PrP resembled WT PrP in supporting peripheral nerve myelination. Prion-infected S1 and S3 transgenic mice both accumulated similar low levels of PrP^S^^c^ and infectious prion particles, but differed in their clinical presentation. Unexpectedly, S3 PrP overproduced C2 fragment in the brain by a mechanism distinct from metal-catalysed hydrolysis reported previously. OR flexibility is concluded to impact diverse biological endpoints; it is a salient variable in infectious disease paradigms and modulates how the levels of PrP^S^^c^ and infectivity can either uncouple or engage to drive the onset of clinical disease.

## Introduction

**Figure 1:**
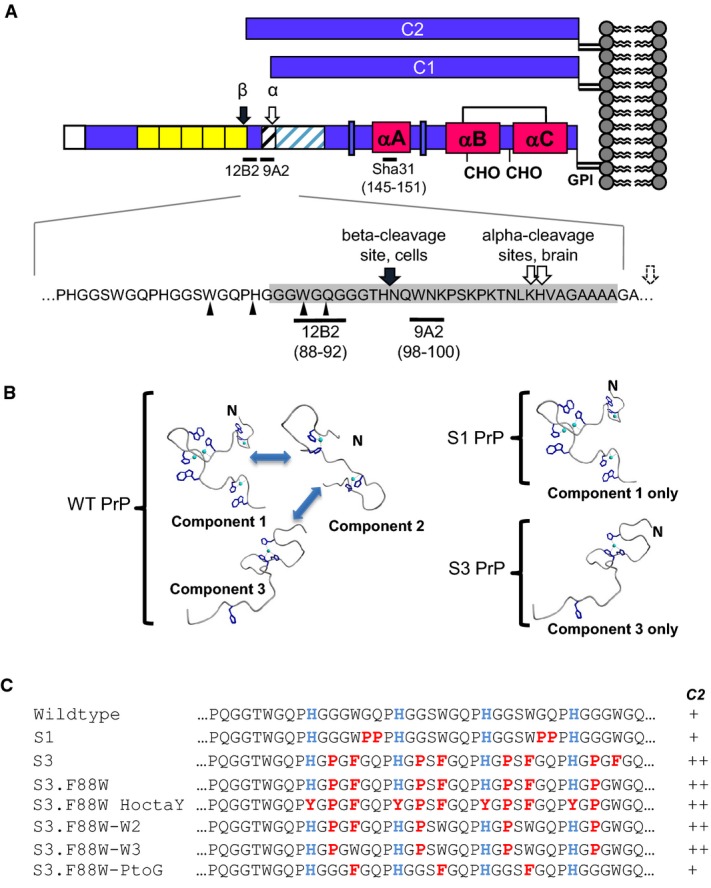
Copper-binding conformations and PrP octarepeat sequence mutations
A The structure of full-length PrP and derivative C1 and C2 fragments with an enlarged view of the cleavage sites is shown. The C2 (β) site was mapped in HpL3-4 cells by (Mange *et al*, 2004). Ragged C1 (α) cleavage sites in brain are shown (Chen *et al*, 1995), and a dotted arrow indicates that additional sites have been mapped rightward of this position *in vitro* (McDonald *et al*, 2014). Ragged N-terminal cleavage sites of PrP27-30 from prion-infected mouse brain are indicated by small arrows (Howells *et al*, 2008). The positions of antibody epitopes are also shown (residues in brackets). Grey shading reflects the minimum length for a C2 fragment defined operationally by reactivity with 12B2 antibody.B S1 and S3 alleles, by virtue of limited geometries (modified from Chattopadhyay *et al*, 2005), may have access to distinct subsets of the full range of biological activities of WT PrP, which has access to interchangeable component 1, 2 and 3 geometries.C Sequences of synthetic PrP alleles differing in the octarepeat region of mouse PrP. Conserved histidines in octarepeats 2–5 are coloured blue and missense mutations are shown in red. Overproduction of C2 fragment is indicated by ++ signs to the right of the panel.

Prion diseases such as Creutzfeldt-Jakob disease (CJD) and bovine spongiform encephalopathy (BSE) are transmissible neurodegenerative disorders. The cellular form of the prion protein (PrP^C^), encoded by the *Prnp* gene, is displayed on the cell surface by a glycophosphatidylinositol (GPI) anchor and serves a precursor role, undergoing a change from a mainly alpha-helical structure to the beta-rich conformation of PrP^Sc^ during disease. Its function is debated such that it could be involved in neuroprotection (Kuwahara *et al*, 1999; Weise *et al*, 2004; Watt *et al*, 2005; Rangel *et al*, 2007), copper homeostasis (Pauly & Harris, 1998; Herms *et al*, 1999; Millhauser, 2004, 2007), signal transduction (Mouillet-Richard *et al*, 2000; Spielhaupter & Schatzl, 2001; Chiarini *et al*, 2002) or peripheral myelin maintenance (Nishida *et al*, 1999; Bremer *et al*, 2010). In structural terms, PrP^C^ is composed of a flexible N-terminal region (including a charged patch), two hexarepeats, five tandem repeats of eight amino acids forming an octarepeat region (OR), a hydrophobic linker region sometimes referred to as the ‘HD’ (hydrophobic domain) and a C-terminal globular domain (Fig1A).

A number of similarities have emerged between prion diseases and Alzheimer's disease (AD), two having particular relevance here. The first concerns the crucial role of endoproteolytic events. One important misfolded protein species in AD is the amyloid beta peptide, Aβ, this being liberated by the concerted actions of the endoproteases called β- and γ-secretase on the beta amyloid precursor protein, APP. Conversely, α-secretase processing of APP by a disintegrin and metalloproteases (ADAM) generates a benign peptide called P3 and prevents amyloid formation. Notably, PrP^C^ is subject to analogous endoproteolysis events (Fig1A); α-cleavage of PrP^C^ occurs adjacent to the linker region at residue 110 to generate a fragment called C1 and is also attributed to the action of an ADAM protease (Chen *et al*, 1995; Jimenez-Huete *et al*, 1998; Vincent *et al*, 2000, 2001; Laffont-Proust *et al*, 2005; Walmsley *et al*, 2009). Furthermore, α-cleavage of PrP^C^ serves a benign role insofar as formation of C1 precludes the formation of PrP^Sc^ upon exposure to infectious prion inocula as the protease-resistant core of PrP^Sc^ called PrP27-30 is longer, starting in the vicinity of residue 90 (Prusiner *et al*, 1984; Oesch *et al*, 1985). Indeed, recent studies have shown that expression of C1 PrP may be protective during disease (Lewis *et al*, 2009; Westergard *et al*, 2011). Conversely, β-processing to make C2 PrP is thought to be conducive to prion replication and while *in vivo* cleavage of APP by β-secretase, BACE1, is well understood, this is not the case for C2 PrP, where facilitated cleavage has only been produced *in vitro* (McMahon *et al*, 2001; Watt *et al*, 2005).

The second similarity between prion disease and AD concerns the assignment of crucial pathogenic pathways. While PrP and Aβ are the aetiological causative agents defined by many transmission studies and genetics, respectively, the exact physical forms of the agents that cause toxicity at disease end-stage—perhaps oligomeric assemblies for Aβ and perhaps a ‘lethal’ PrP form denoted PrP^L^—have proven difficult to study (Legname *et al*, 2004; Sandberg *et al*, 2011; Aguzzi & Falsig, 2012; Benilova *et al*, 2012). In the case of prions, instances of apparent uncoupling between the accumulation of protease-resistant PrP and clinical signs of disease caused confusion in the field and were once used to argue against the validity of the prion hypothesis (Lasmezas *et al*, 1997).

The studies presented here focus upon PrP N-terminal sequences. Though not obligatory for laboratory infection of rodents (Fischer *et al*, 1996; Flechsig *et al*, 2000), extra copies of the octarepeats to make an expanded OR are associated with familial prion diseases with heterogeneous clinical presentations, being variously classified as genetic CJD, Gerstmann–Sträussler–Scheinker syndrome or psychiatric disorders (Goldfarb *et al*, 1991; Laplanche *et al*, 1999; Moore *et al*, 2001; Mead *et al*, 2006). N-terminal sequences are thought to bind a number of partners (Beland & Roucou, 2012), with binding of Cu(II) and Zn(II) ions by the OR being an area of active interest (reviewed in Millhauser, 2007). Based on the concept that the N-terminal region of metal-free PrP^C^ (apo-PrP^C^) is natively unstructured but that different stoichiometries of metal binding can impart different structures upon the OR (called ‘component 1’, ‘component 2’ and ‘component 3’ (Chattopadhyay *et al*, 2005); Fig1B), we used structure-guided design to create new PrP alleles (Fig1C). While conformation components 1, 2 and 3 may interchange depending upon local physiological conditions, we were interested in the concept that they have different functions and sought to restrict inter-conversion to study their biological properties individually. One new PrP allele called ‘S1’ locks the OR in an extended conformation (component 1 metal-binding geometry), and this conformation is conducive to binding up to 4 copper ions per OR. Another allele called ‘S3’ locks PrP in a compact conformation that can bind only one copper ion per OR, corresponding to component 3 metal-binding geometry (the intermediate conformation component 2 was not modelled in our studies). The S1 and S3 PrP alleles were expressed in cells and transgenic mice and revealed a number of unexpected interrelationships between OR flexibility, physiological function, pathogenesis of infectious disease and β-cleavage.

## Results

### Increased C2 fragment levels are associated with S3 PrP, not S1 PrP

**Figure 2:**
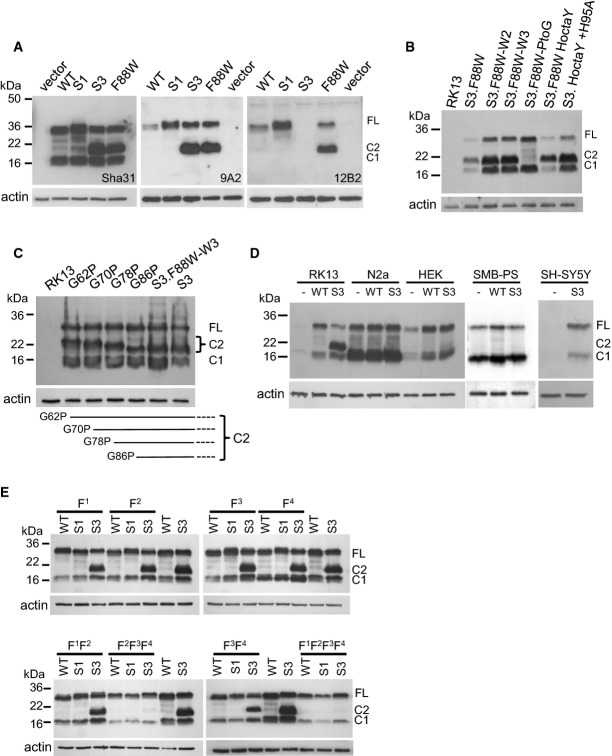
Western blot analysis of lysates from cells transiently transfected with plasmids encoding PrP mutants
A Lysates from RK13 cells transfected with WT, S1, S3 and S3.F88W PrP were probed with antibody Sha31, 9A2 and 12B2 after digestion with PNGaseF.B, C Additional PrP mutants were examined by Western blot with Sha31 after PNGaseF digestion of RK13 cell lysates. A schematic of the different lengths of C2 caused by single mutations in the OR is shown in the lower portion of (C).D Lysates of N2a, HEK, SMB-PS and SH-SY5Y cells not transfected (−) and transiently transfected with WT PrP or S3 PrP plasmids were digested with PNGaseF and analysed by Western blot using Sha31.E Analysis of lysates from RK13 cells transfected with plasmids incorporating Phe substitutions into the hydrophobic domain of WT PrP, S1 PrP and S3 PrP. Lysates were PNGaseF-digested before Western blot using the Sha31 antibody. F^1^: A114F, F^2^: G118F, F^3^: G122F, F^4^: G126F
Source data are available online for this figure.

Using a series of synthetic peptides and electron paramagnetic resonance (EPR) analysis of metal protein complexes, we identified residue substitutions in ORs 2–5 that constrained PrP to favour component 1 or 3 binding geometry (Supplementary Fig S1) (Chattopadhyay *et al*, 2005). We then derived the corresponding mutant alleles of the *Prnp* gene S1 PrP and S3 PrP (as well as a WT control construct equipped with the same 5′ UTR leader sequences) that could encode the conformationally constrained proteins (Fig1B and C). To confirm expression, the plasmids were transiently transfected into RK13 cells and lysates analysed for PrP^C^ by Western blot with the antibody Sha31 (Feraudet *et al*, 2005) after PNGaseF treatment to remove glycans (Fig2A). The S1 and S3 alleles were glycosylated in a similar manner to WT PrP and showed similar localization within permeabilized RK13 cells (Supplementary Fig S2A and B). Deglycosylated samples showed full-length (FL) PrP and C1 PrP, but cells expressing S3 PrP also had increased levels of the C2 fragment (Fig2A). To confirm these data, PrP antibodies 9A2 and 12B2 were used (Fig2A), which do not recognize the C1 fragment due to the location of their epitopes (Fig1A). No signal was observed for S3 PrP using the 12B2 antibody, but a tryptophan residue of this antibody's epitope is altered in S3 PrP. Since this amino acid change was close to the ‘beta’ cleavage site observed in infected tissue (Chen *et al*, 1995; Mange *et al*, 2004), we reverted a phenylalanine in S3 to the WT residue at this position, tryptophan (S3.F88W). Using Sha31 and 9A2 antibodies, we observed that the C2 fragment remained prominent in RK13 cells expressing the F88W plasmid. When the 12B2 antibody was applied, signal for FL and C2 PrP returned (Fig2A), indicating that a cleavage site to generate C2 PrP from the S3 allele is positioned N-terminal to residue 88 (Fig1A).

Based on these results, we generated stable RK13 clones expressing WT PrP (WT-10), S1 PrP (S1–29), S3 PrP (S3–27) and S3.F88W PrP (F88W-5). Cell surface proteins were biotin-labelled using a membrane-impermeable reagent and trypsin-digested following specific chase times (Supplementary Fig S2C). The mutant S1 and S3 full-length PrP, as well as the C2 fragment, was found to be accessible on the surface of cells, and the mutants were internalized with similar kinetics to a WT PrP control.

### Effects of histidine substitutions and cell type on C2 cleavage of S3 PrP

The levels of endogenous copper in our cell media were determined to be between 0.46 μM and 1.4 μM (average = 0.85 μM ± 0.14 SEM, *n* = 5), but a series of experiments modulating these baseline values by adding chelators or metals or by altering cell plating density failed to alter C2 formation by the S3 or WT PrP alleles (Supplementary Figs S3, S4 and S5). As PrP^C^'s component 3 geometry binds copper using OR histidines (Stöckel *et al*, 1998; Viles *et al*, 1999; Chattopadhyay *et al*, 2005), we assessed the effect of mutating these residues to tyrosine (‘S3.F88W HoctaY’ allele). As presented in Fig2B, the C2 fragment was observed in cells expressing the S3.F88W HoctaY mutant, indicating that the increased levels of C2 fragment associated with S3 and S3.F88W PrP were prompted by effects of the residue substitutions and unrelated to metal occupancy *in vivo*. This conclusion was emphasized by a further mutant that also produced abundant C2, S3.HoctaY.H95A, where His 95 of the non-OR ‘copper site 5′ (Jackson *et al*, 2001) was replaced by an alanine residue to result in a net perturbation of all major copper-binding sites in PrP^C^'s N-terminus.

To define the residues responsible for altered cleavage of PrP, we reverted substitutions within S3 PrP back to their WT status (Fig1C) and transfected these plasmids into RK13 cells. The C2 fragment was present in lysates from cells expressing S3.F88W-W2 (substitutions to proline and phenylalanine in octarepeat 2 and prolines in octarepeats 3–5) and S3.F88W-W3 (substitutions to proline in octarepeats 2–5), but was absent from cells expressing S3.F88W-PtoG, which had substitutions to phenylalanine in octarepeats 2–4 (Fig2B). This indicated that the PHGGGWGQ-> PHGPGWGQ proline substitutions within S3 PrP determine C2 cleavage. To test this concept further, we mutated individual prolines within octarepeats 2, 3, 4 and 5. Proceeding in reverse order from octarepeat 5 through octarepeat 2 (i.e. proceeding in an N-terminal direction), the size of the C2 fragment increased accordingly and was compatible with one cleavage per PHGPGWGQ-containing octarepeat (Fig2C).

Endoproteolysis of PrP^C^ to C2 fragments has previously been suggested to occur through reactive oxygen species (ROS) generated by the binding of copper to the PrP OR (McMahon *et al*, 2001; Pushie & Vogel, 2008, 2009), where the free radicals react with a peptidyl bond and cause cleavage. However, the C2 fragment was produced in cells expressing S3.F88W HoctaY, which cannot bind copper via the OR. Moreover, the mechanism proposed from previous *in vitro* studies only requires PrP, a ROS-generating system, and copper for an autocatalytic reaction; however, when the S3 PrP plasmid was introduced into four cell lines other than RK13, the C2 fragment was not detected (Fig2D). This indicates that other factors may affect cleavage, such as a protease present in RK13 cells yet absent from N2a, HEK, SH-SY5Y and SMB-PS cells.

### Hydrophobic domain substitutions impact β-cleavage of S3 PrP

Due to the possibility of N-terminal/C-terminal interactions occurring in *cis* (Thakur *et al*, 2011; Sonati *et al*, 2013; Spevacek *et al*, 2013), we investigated whether bulky side-chain substitutions that might interfere with the flexibility of the intervening linker region could affect C2 cleavage. As seen in Fig2E, when residues 114G, 118G, 122G and 126G were mutated to phenylalanine individually or in pairs (F^1^F^2^; F^3^F^4^), there was no effect on the cleavage of S3 PrP. However, if compounded into triple or quadruple mutations (F^2^F^3^F^4^ and F^1^F^2^F^3^F^4^), C2 production was attenuated. There was no visible influence of the phenylalanine residue substitutions on PrP with WT or S1 versions of the OR, and cleavage to the C1 fragment was maintained in cells expressing all mutation variants (Fig2E).

### Effects of S1 and S3 alleles in uninfected animals

**Figure 3:**
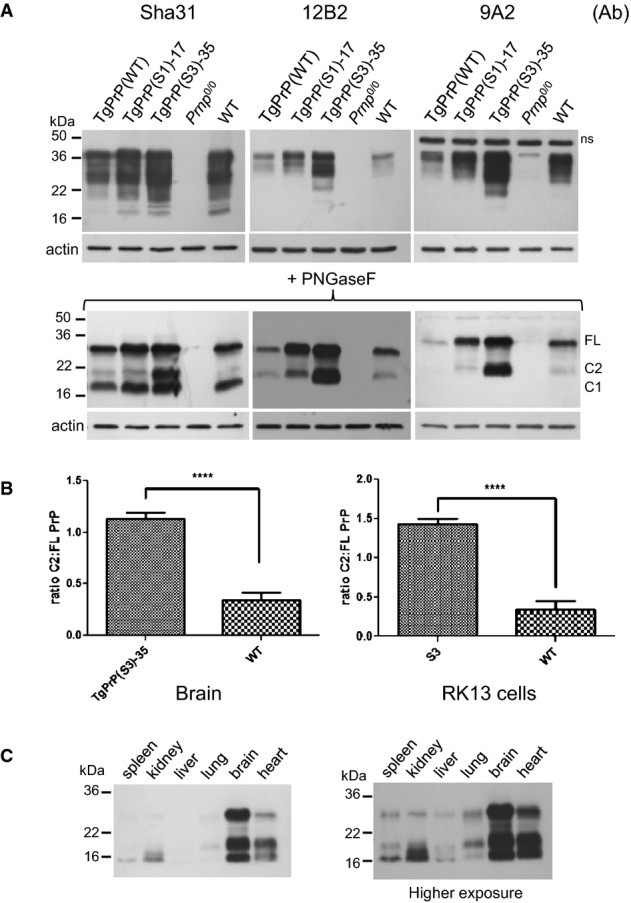
PrP expression in transgenic mice
A Brain homogenates of TgPrP(WT), TgPrP(S1)-17 and TgPrP(S3.F88W)-35 mice were analysed by Western blot before and after PNGaseF digestion using the PrP antibodies Sha31, 12B2 and 9A2. *Prnp*^0/0^, and WT brain homogenates were used as controls.B The ratio of C2 to full-length PrP in TgPrP(S3.F88W)-35 and WT mice (left panel, *n* = 7, *P* = 1.46E-06) and in RK13 cells expressing WT and S3 PrP (right panel, *n* = 5, *P* = 4.01E-05) is shown. Values are presented as mean ± SEM. Unpaired, two-tailed *t*-test, ****P < 0.0001.C Different tissue homogenates from a TgPrP(S3.F88W)-35 mouse were PNGaseF-digested and analysed for PrP using the antibody 1A6. FL, full-length PrP; ns, non-specific signal detected in the immunoblotting procedure.
Source data are available online for this figure.

Next, Tg mice were created using a ‘half-genomic’ construct expression vector derived from the mouse *Prnp* locus (Borchelt *et al*, 1996; Fischer *et al*, 1996). Since the S3.F88W PrP allele was associated with increased levels of the C2 fragment like S3 PrP (Fig2A), this was used for transgenesis instead of S3 PrP, to allow the 12B2 antibody to be used for protein expression analysis. Following standard pronuclear microinjection procedures, we established 6 new transgenic lines from founder animals. Brain homogenates of adult Tg mice (i.e. TgPrP(WT), TgPrP(S1)-17 and TgPrP(S3.F88W)-35) were analysed for PrP by Western blot using the antibodies Sha31, 12B2 and 9A2 (Fig3A). High levels of the C2 fragment were observed in TgPrP(S3.F88W)-35 mice. After normalization to full-length brain PrP, densitometric analysis indicated an increment in excess of three-fold (WT C2:C1 0.34:1 ± 0.07 SEM *n* = 7 versus TgS3.F88W 1.14:1 ± 0.06, *P* = 1.46E-06; Fig3B), which is consistent with densitometric analysis from S3 vs WT PrP-expressing RK13 cells (Fig3B right panel). Furthermore, albeit present at lower levels, C2 fragment was detected in non-transgenic mice using the 12B2 antibody, confirming that WT PrP^C^ is cleaved at a position N-terminal to amino acid 88 to produce the C2 fragment under physiological conditions. We assessed the amount of FL PrP^C^ and its C1 and C2 fragments in uninfected transgenic mice by Western blot analysis of brain homogenates using six distinct antibodies (Table1, Supplementary Fig S6). TgPrP(S3.F88W) lines 35 and 14 over-expressed FL PrP approximately 3× WT levels. Two lines of TgPrP(S1) mice had 1.7–1.8× the amount of PrP compared with WT mice, whereas a third line TgPrP(S1)-39 expressed low levels of PrP (0.7×) that were similar to TgPrP(WT) (Supplementary Fig S6; Mays *et al*, 2014). Using synchrotron-based techniques for neuroanatomical mapping of trace metals (Pushie *et al*, 2011), we confirmed altered distribution of Cu and Zn ions in TgPrP(S1) and TgPrP(S3.F88W) mice (M.J. Pushie, A. McDonald, K.H. Nienaber, R. Aglietti, A. Lau, D. Westaway, G.N. George, in preparation).

**Table 1:** Levels of full-length PrP and PrP fragments in transgenic mice relative to wild-type animals

Genotype	Full-length (FL) PrP^C^ determined by Western blot^a^	FL+ C2 PrP^C^ determined by Western blot^a^	FL+ C2 PrP^C^ determined by CDI with 12B2 antibody^b^
WT	1.00	1.00	1.00
TgPrP(S1)-17	1.69 ± 0.20	1.49 ± 0.17	1.12 ± 0.14
TgPrP(S1)-19	1.83 ± 0.20	1.58 ± 1.8	1.11 ± 0.14
TgPrP(S1)-39	0.74 ± 0.12	0.57 ± 0.09	0.29 ± 0.04
TgPrP(S3.F88W)-35 male mice	2.93 ± 0.29	4.42 ± 0.67	3.01 ± 0.50
TgPrP(S3.F88W)-14	2.97 ± 0.26	4.56 ± 0.68	4.74 ± 1.32
TgPrP(WT)	0.79 ± 0.15	0.74 ± 0.15	0.43 ± 0.1

a Averaged data from adolescent uninfected mice obtained from the use of 12B2, SAF83, 2D6, 9A2, VRQ61 and 1A6 antibodies in Supplementary Fig S6. Apart from the TgPrP(S3.F88W)-35 line, with data just derived from male mice (also applicable to Tables2 and 3), data were derived from both genders. Ages for the samples range between 37 and 138 days.

b The 12B2 epitope lies outside the boundaries of the C1 fragment and hence will detect the sum of full-length PrP^C^ plus the C2 fragment. These estimates represent PrP^C^-containing velocity gradient fractions (fractions 9 and 10) of brain homogenates of uninfected mice at ages 37–80 days; data are derived from 3 mouse brain homogenates per genotype pooled and then gradient-fractionated.

When different tissues of a TgPrP(S3.F88W)-35 mouse were analysed, the ratios of C2 fragment to C1 or full-length PrP^C^ differed (Fig3C). In the spleen, the dominant band was the C1 fragment, but the C2 fragment was strongest in the heart and lung (Fig3C). Additionally, the size of the C2 fragment in the kidney appeared to be smaller compared to other tissues. When considered alongside our studies for mutant PrP in tissue-culture cells, these data do not provide strong support for the concept that C2 cleavage of S3 PrP *in vivo* derives from a metal-assisted hydrolysis event mediated by the PrP polypeptide chain itself.

Although increased levels of C2 fragments occur in prion disease states (Chen *et al*, 1995; Yadavalli *et al*, 2004; Dron *et al*, 2010) and mice programmed to express a C2 fragment develop a spontaneous disease syndrome (Colby *et al*, 2010), we were unable to detect spontaneous neurological disease in TgPrP(S1) and TgPrP(S3.F88W) mice at nearly 2 years of age, nor the presence of a PK-resistant PrP species by Western blot of brain homogenates from these mice (Supplementary Table S1).

**Figure 4:**
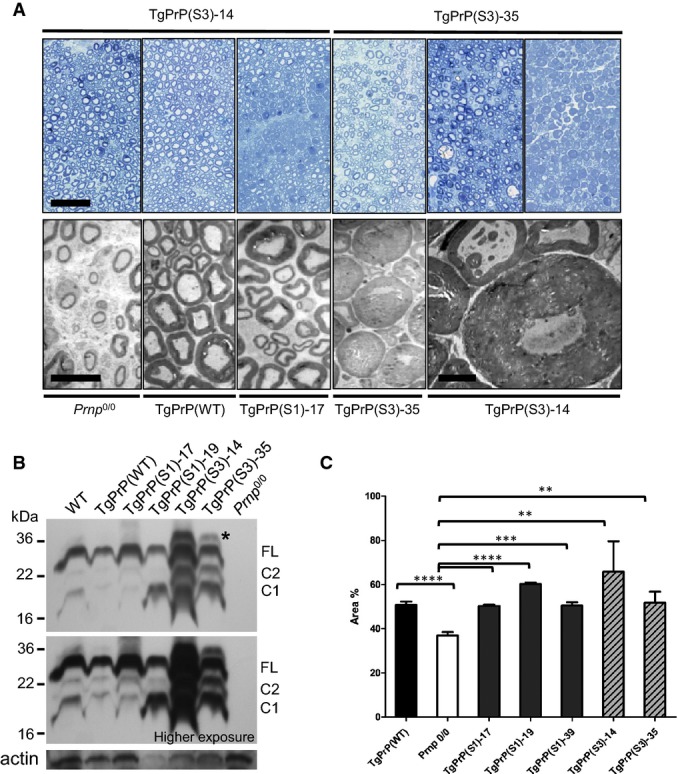
Variable rescue of the demyelinating polyneuropathy of *Prnp*^0/0^ mice by the S3 PrP allele
A Sections of sciatic nerve from TgPrP(S3.F88W)-14 female mice (all 610 days old) and TgPrP(S3.F88W)-35 female mice (614, 570 and 614 days old, respectively) were analysed using toluidine blue staining (upper row), with the third panels for each line showing a hypermyelinated phenotype. Scale bar in left panel represents 50 μm. The lower rows show EM analyses of mice of the indicated genotypes. Scale bar is 10 μm for panels 1–4, with the fifth panel showing a high power view to illustrate hypermyelinated fibres in a mouse expressing S3.F88W PrP (scale bar, 5 μm).B Western blot analysis of sciatic nerve protein extracts (10 μg) from female mice after PNGaseF treatment shows a similar hierarchy of PrP expression in Tg lines to that of brain (see Fig3). The asterisk indicates a greater than full-length fragment present in TgPrP(S3.F88W) mice, possibly corresponding to incomplete removal of the N-terminal signal peptide.C Quantification of fibre morphology in aged animals (492–608 days old), representing the percentage of the nerve occupied by fibres versus the total cross-sectional area of the nerve. The S1 and S3.F88W alleles rescue *Prnp*^0/0^ nerves to a similar percentage as the WT allele, but the variance and *P*-value is greater for the two Tg lines expressing the TgPrP(S3.F88W) allele. Unpaired, two-tailed *t*-test, **P < 0.01, ***P < 0.001, ****P < 0.0001. Sample sizes and exact *P*-values compared with *Prnp*^0/0^ (*n* = 9) were as follows: TgPrP(WT) *n* = 6, *P* = 3.63E-05; *Prnp*^0/0^, *n* = 9; TgPrP(S1)-17 *n* = 5, *P* = 3.54E-05; TgPrP(S1)-19 *n* = 3, *P* = 6.35E-06; TgPrP(S3.F88W)-14 *n* = 3, *P* = 3.70E-03; and TgPrP(S3.F88W)-35 *n* = 7, *P* = 7.18E-03.
Source data are available online for this figure.

Outside of the CNS, independent lines of PrP knockout mice exhibit a demyelinating polyneuropathy (DMP) (Nishida *et al*, 1999; Bremer *et al*, 2010), which is also present in our colony of Zrch1 *Prnp*^0/0^ mice at ages greater than or equal to 10 months. To assess the abilities of the S1 and S3.F88W alleles to rescue the DMP phenotype, we examined the status of the sciatic nerve in aged S1 and S3. F88W mice versus *Prnp*^0/0^ mice (Fig4A, Supplementary Fig S7). Here, genetic complementation analysis revealed that three TgPrP(S1) lines and the TgPrP(WT) line rescued the DMP syndrome (Supplementary Fig S7). These animals express both full-length and C1 PrP in sciatic nerves (4Fig B), as is the case for the CNS (Fig3A), which is compatible with the contention that α-cleavage may contribute to peripheral nervous system (PNS) maintenance (Bremer *et al*, 2010). Morphometric analysis of sciatic nerves established that mice with DMP syndrome had less than 40% of nerve cross-sectional area occupied by fibres, whereas this figure was above 50% for animals with genetic rescue of the syndrome (Fig4C, Supplementary Fig S8). With respect to the amount of PrP^C^ needed to prevent the DMP syndrome, TgPrP(WT) mice exhibit a low level of expression (Table1 and Mays *et al*, 2014), which is similar to that of *Prnp*^0/+^ mice with only one copy of the *Prnp* gene per diploid genome (these mice do not exhibit DMP (Bremer *et al*, 2010)). Similarly, TgPrP(S1)-39 mice with expression below endogenous levels (Table1) do not exhibit the DMP syndrome. Analysis of aged TgPrP(S3.F88W)-35 and TgPrP(S3.F88W)-14 mice (Fig4A) revealed genetic rescue of the DMP syndrome, albeit with a greater degree of variability. Most TgPrP(S3.F88W)-35 and TgPrP(S3.F88W)-14 mice had nerve morphology with a uniform distribution of myelinated fibres resembling the WT state. However 20% of TgPrP(S3.F88W) mice had areas of apparently normal fibre morphology lying adjacent to fields of hypermyelinated fibres (Fig4A), with transmission electron microscopy of samples from the same animals also revealing normally myelinated fibres lying adjacent to hypermyelinated fibres. This variation in gross anatomy was observed alongside larger net variance in percentage of the nerve occupied by fibres for both Tg lines expressing the S3 PrP allele (Fig4C) and less significant *P*-values versus knockout controls (*P* < 0.01 against *Prnp*^0/0^ for both Tg lines expressing S3 PrP versus *P* < 0.001 or lower for Tg lines expressing the S1 PrP allele).

### Primary passage of the RML prion isolate into TgPrP(S1) and TgPrP(S3.F88W) mice

We next assessed the responses of TgPrP(S1)-17 and TgPrP(S1)-19 mice upon challenge with infectious prions using the RML isolate of mouse-adapted scrapie. As presented in Table2, mean survival times of TgPrP(S1)-17 mice and TgPrP(S1)-19 mice were either 15 days shorter or indistinguishable from WT mice even though these Tg lines express ∽1.7-fold more full-length PrP^C^ substrate (Table1; ****P = 0.0001 and ns at *P* = 0.19, respectively), with increased expression level being a parameter that normally decreases incubation times (Carlson *et al*, 1994). This indicates that a facet of pathogenesis is attenuated in mice expressing the S1 PrP^C^ allele. TgPrP(WT) mice (Mays *et al*, 2014) comprised a further control for this experiment; this Tg line had low amounts of PrP and survival times were greater than 400 days, in a similar fashion to hemizygous mice producing ∽50% the amount of PrP^C^ of WT mice (Bueler *et al*, 1994; Fischer *et al*, 1996). More notably, both lines of TgPrP(S3.F88W) mice had incubation times at least 60 days shorter than WT mice (Table2): 84 and 89 day averages for TgPrP(S3.F88W)-35 and TgPrP(S3.F88W)-14 (****P = 1.75E-18 and 2.44E-19, respectively versus WT control).

**Table 2:** Survival times for transgenic mice following intracerebral challenge with the RML isolate of mouse-adapted prions

Mouse genotype	Genetic background	Mean survival ± SEM	*N*
TgPrP(S3.F88W)-35	FVB *Prnp*^0/0^	84.1 ± 2.6	12
TgPrP(S3.F88W)-14	FVB *Prnp*^0/0^	89.1 ± 2.5	16
TgPrP(S1)-17	FVB *Prnp*^0/0^	139.1 ± 2.8	22
TgPrP(S1)-19	FVB *Prnp*^0/0^	152.5 ± 2.5	17
TgPrP(S1)-39	FVB *Prnp*^0/0^	424.4 ± 9.2	9
TgPrP(WT)	FVB *Prnp*^0/0^	398.3 ± 8.1	12
WT	FVB	155.9 ± 2.0	16

**Figure 5:**
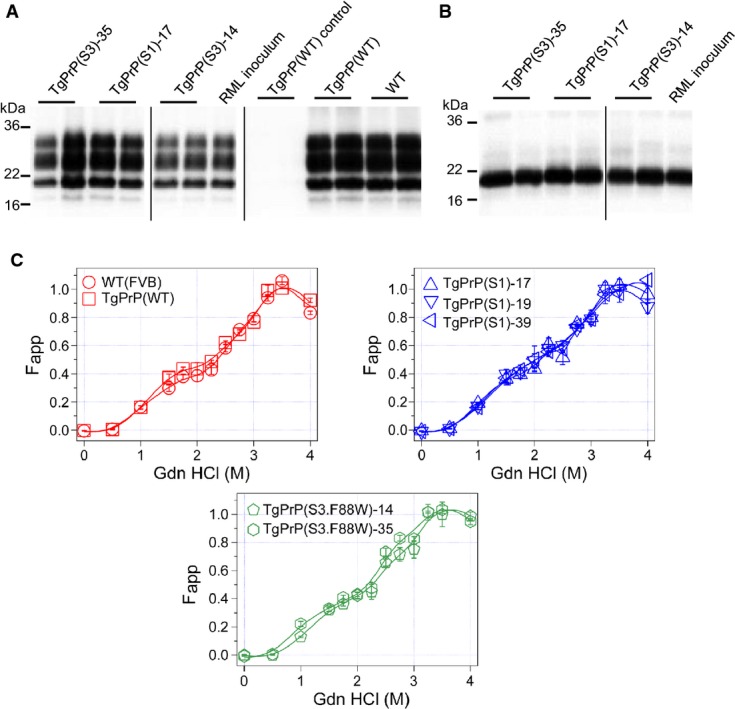
Biochemical properties of PrP^Sc^ from infected transgenic mice
A, B Infected brain homogenates were PK-digested and analysed by Western blot using (A) Sha31 antibody and (B) after PK and PNGaseF digestion using Sha31.C Conformational stability assay on RML-infected brain homogenates incubated with increasing concentrations of GdnHCl. The best-fit alignments of the data are presented. Genotypes are noted on the individual panels.
Source data are available online for this figure.

TgPrP(S1)-17 and TgPrP(S3.F88W)-35 animals were also challenged with prions from cases of natural scrapie and classical BSE. Animals were held for over 400 days without signs of clinical disease (scrapie, range 416–594 dpi; classical BSE, range 445–537 dpi; Supplementary Table S2). These data indicate that typical barriers against the transmission of foreign prions from one species of mammal to another were not circumvented by expression of the S1 and S3.F88W PrP alleles (and in this respect did not differ from the performance of WT PrP (Fraser *et al*, 1992)). Barriers to prion infections can also be caused by PrP coding sequence polymorphism within a species (Carlson *et al*, 1994). These transmission barriers derive from a change in the primary structure of PrP^C^ substrate and can result in altered selection pressures that will facilitate the emergence of new strains (Collinge & Clarke, 2007). We assessed this possibility for prions deriving from the use of S1 and S3.F88W allelic forms of PrP^C^ versus WT PrP^C^, employing PK digestion to examine changes in glycosylation profile or fragment size of PrP^Sc^, as well as observing differences in susceptibility to PK digestion after exposure to increasing concentrations of the denaturant guanidine that may indicate a change in structure. However, using the C-terminal antibody Sha31, electrophoretic profiles of PK-digested PrP species from brain homogenates of the transgenic mice were similar to the RML inoculum; this resemblance remained after incubation with the glycosidase PNGaseF, when a single band was present in all three genotypes (Fig5A and B). Similarly, guanidine denaturation curves of the RML prions propagated in WT mice were similar to those in TgPrP(WT) mice (Fig5C, Supplementary Fig S9). The guanidine denaturation curves of the RML prions propagated in different Tg lines deriving from the same S3.F88W or S1 PrP constructs (represented by 2 and 3 Tg lines, respectively) were not only similar to each other, but also resembled the denaturation profile of the WT prototypes. These data argue against the emergence of a new prion strain(s) in TgPrP(S1) and TgPrP(S3.F88W) mice infected with the RML prion isolate.

**Figure 6:**
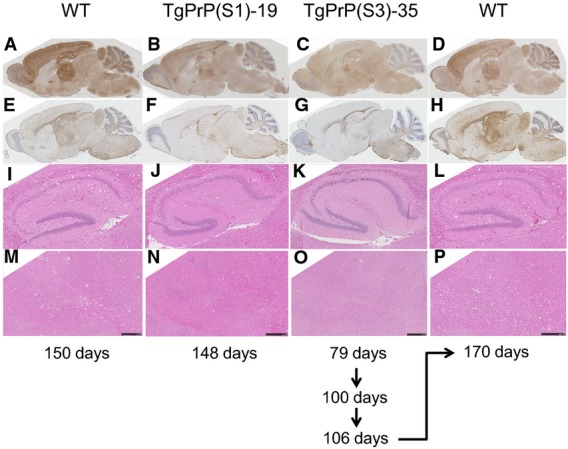
Pathology of RML-infected mice
A-H The pattern of PrP^Sc^ deposition (A–D) and GFAP immunostaining (E–H) is presented.I-P A higher power view of H&E stained hippocampus (I–L) and thalamus (M–P) to reveal vacuolation is presented.
Data information: Animals of the same genotype are shown within a column, and incubation times of the presented animals with the RML prion isolate are shown (days). Mice expressing PrP S1 and S3 alleles (central columns) exhibit attenuated PrP^Sc^ deposition, gliosis and spongy change. Pathological features of the RML isolate are not altered upon sequential passage through TgPrP(S3.88W)-35 mice (see schematic at bottom of the diagram indicating sequential passages and finally, passage back into WT mice, compare the data in first and fourth columns).

In terms of neuropathology, RML prion-challenged TgPrP(S1) and TgPrP(S3.F88W) exhibited reduced levels of deposition of protease-resistant PrP^Sc^, reduced gliosis and reduced spongiosis (Fig6). They did not diverge qualitatively from WT prototypes in that the pattern of PrP^Sc^ deposition did not expand to include novel neuroanatomical structures. Within these immunostaining signals, punctate staining could be seen in some but not all animals, in the corpus callosum, which is analogous to a profile present within WT animals infected with RML prions (Fig6). Interestingly, the original signature of PrP^Sc^ deposition by the RML agent present in WT animals was retained after three passages through TgPrP(S3.F88W) mice, indicating that expression of the S3.F88W PrP allele did not favour the appearance of a new prion strain with different pathological properties. Incubation times were longer than for direct passage into WT mice (164.3 ± 3.5 (SEM) versus 155.9 ± 2.0 days (*n* = 12, *n* = 16; *P* = 0.05)) and were consistent with a drop in titre measured by other techniques, as presented below.

### Profiling prions from infected TgPrP(S1) and TgPrP(S3.F88W) mice

We carried out further analytical chemical procedures to increase our understanding of the pathogenic process in TgPrP(S1) and TgPrP(S3.F88W) mice. We separated protein species on the basis of size by velocity gradient fractionation of infected brain samples performed in the presence of the detergent sarkosyl. Next, the conformation-dependent immunoassay (CDI), which provides a signature of global protein folding via the accessibility of antibody epitopes, was used to assess the gradient fractions. Using these two technologies, and by performing analyses of gradient fractions in the presence or absence of proteinase K, we compiled an inventory of oligomeric PrP^Sc^ species: this inventory included net levels of protease-sensitive and protease-resistant (r) forms of PrP^Sc^ (rPrP^Sc^), as well as PrP^C^ levels in fractions at the top of the gradients (Tables1 and 3). As a prelude to these experiments, control analyses excluded the possibility that S1 and S3 allelic forms of PrP^C^ from uninfected mice had pre-existing conformational changes within the 12B2 antibody epitope used for CDI assays (Supplementary Fig S10). The sedimentation analyses showed a similar pattern of accumulation of PrP^Sc^ oligomeric species from RML-infected WT, TgPrP(S1) and TgPrP(S3.F88W) animals (Supplementary Fig S11). A different parameter, the denatured to native ratio of PrP^Sc^ (i.e. with and without exposure to 4 M GdnHCl; ‘D/N ratio’), revealed subtle distinctions in the PrP^Sc^ populations in the brains of RML-infected animals after PK treatment (Supplementary Fig S12). PrP^Sc^ derived from RML-infected WT (*Prnp*^a^), TgPrP(S1)-17 and TgPrP(S1)-19 mice behaved in a similar manner with an increased D/N value after PK treatment, whereas PrP^Sc^ from infected TgPrP(S3.F88W)-14 and TgPrP(S3.F88W)-35 mice exhibited a drop in ratio. The assay used to obtain these data uses the 8H4 antibody for PrP capture and a Europium-labelled version of the 12B2 antibody for detection (with two epitopes located at residues 175–185 and 88–92, respectively). As alterations were not detected in the C-terminal protease-resistant core of PrP^Sc^ (Fig5) and since a CDI signal is only generated if both epitopes are available, the drop in D/N ratio leads us to infer a shift in accessibility of the 12B2 epitope in rPrP^Sc^ populations of infected TgPrP(S3.F88W) mice.

**Table 3:** PrP species from brain homogenates of prion-infected mice analysed by CDI after velocity centrifugation

Mouse line^a^	PrP^C^ (ng/ml) ± SEM	PrP^Sc^ (ng/ml) ± SEM	rPrP^Sc^ (ng/ml) ± SEM	PrP^C^/PrP^Sc^ ratio ± SEM	Incubation time (days) ± SEM
WT	757 ± 36.0	1769 ± 69	460 ± 8.1	1.6 ± 0.03	158 ± 5
TgPrP(WT)	274 ± 5.3	1101 ± 39	323 ± 13	0.8 ± 0.01	364 ± 11
TgPrP(S1)-17	414 ± 6	364 ± 18	110 ± 3	3.8 ± 0.06	139 ± 5
TgPrP(S1)-19	748 ± 13	589 ± 12	153 ± 4	4.9 ± 0.03	147 ± 1
TgPrP(S1)-39	172 ± 2.9	299 ± 4	82 ± 2	2.1 ± 0.01	411 ± 14
TgPrP(S3)-35	1408 ± 13.7	288 ± 5	107 ± 3	13.1 ± 0.10	74 ± 1
TgPrP(S3)-14	1816 ± 87.3	265 ± 8	94 ± 2	19.2 ± 0.39	91 ± 5

a All animals were infected with the RML isolate of mouse-adapted scrapie prions.

**Figure 7:**
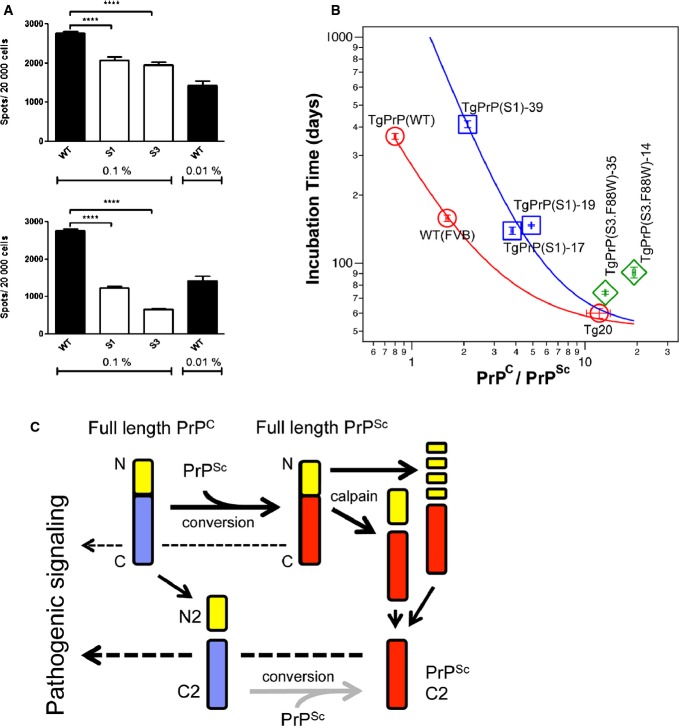
Biochemical properties of PrP^Sc^ assessed by guanidine denaturation and CDI
A Spot counts in the scrapie cell assay using WT, TgPrP(S1)-17 and TgPrP(S3.F88W)-14 brain homogenates (0.1% or 0.01%) from mice infected with RML are shown in the upper panel (WT vs S1 *P* = 2.09E-05 and vs S3 *P* = 4.62E-08) with numbers corrected for PrP^C^ expression levels as per Table1 presented in the lower panel (WT vs S1 *P* = 1.04E-13 and vs S3 *P* = 3.39E-20; two-tailed *t*-test, *n* = 3).B A comparison of incubation time vs PrP^C^:PrP^Sc^ ratio for mice expressing different levels of WT PrP (WT, TgPrP(WT), tga20), S1 PrP (TgPrP(S1)-17, TgPrP(S1)-19, TgPrP(S1)-39) and S3 PrP (TgPrP(S3.F88W)-14, TgPrP(S3.F88W)-35).C Formation and biological activities of PrP species. Synthetic and proteolytic pathways are shown from left to right, while pathways for pathologic signalling from PrP^Sc^ through PrP^C^ and PrP^C^-derived fragments are shown from right to left (dashed arrows). Alternative pathways to remove N-terminal sequences (yellow) from the protease-resistant C-terminal Core (red) in infected cells are shown, while N2 is removed from a protease-sensitive C-terminal core (blue) in healthy cells. Grey arrow indicates that formation of PrP^Sc^ C2 from protease-sensitive C2 is not particularly efficient as mice with high levels of C2 are not more efficient in synthesizing PrP^Sc^. Heavy dashed arrow through protease-sensitive C2 indicates that this species may be adept at pathogenic signalling as mice with lower PrP^Sc^ burden than WT mice succumb to disease earlier. For simplicity, physiological signalling pathways through PrP^C^ species are not shown.

Rather more notably, the CDI assay defined dramatic changes in quantities of protease-sensitive PrP^Sc^, with infected TgPrP(S1) and TgPrP(S3.F88W) mice at disease endpoint both having lower levels than those of WT mice (Table3, Supplementary Fig S12). For total PrP^Sc^ measured versus WT mice, there were averages of 4.2-fold and 6.4-fold reductions for TgPrP(S1) and TgPrP(S3.F88W) mice, respectively. Figures for the analogous reductions in protease-resistant PrP^Sc^ averaged across Tg lines of the same allelic type were 4.0× and 4.6×, respectively. These lower values for PK-sensitive PrP^Sc^ (and rPrP^Sc^) were also paralleled by a drop in infectious titre as established by a homologous serial passage, which revealed a prolongation of incubation times (first passage 84.1 ± 2.6 days, *n* = 12 versus second passage 111.6 ± 3.8, *n* = 5, **P = 0.0018; third passage 95.0 ± 0.6, *n* = 5, **P = 0.0079 versus second passage, *P = 0.015 versus first passage). Secondly, brain homogenates from primary passages into TgPrP(S1) and TgPrP(S3.F88W) mice generated significantly lower spot counts by the standard scrapie cell assay (SSCA; a technique to measure prion infectivity where a cell monolayer is infected with prions and eventually digested with PK to examine the number of cells with PrP^Sc^ and is in some respects similar to a plaque assay for viral particles (Klohn *et al*, 2003)) (Fig7A). The reduction in titre of infectivity derived from TgPrP(S1) and TgPrP(S3.F88W) became more striking when the data were normalized for the extra levels of full-length PrP^C^ present in Tg versus WT mice.

While our data define a compromised ability to replicate RML prions in mice expressing S1 or S3 PrP, the shortened incubation periods in TgPrP(S3.F88W) mice stand in apparent contrast to this effect. To explore this effect and to accommodate the post-translational reduction in PrP^C^ that occurs during the course of disease (Mays *et al*, 2014), we plotted disease incubation times versus endpoint PrP^C^:PrP^Sc^ ratios. Here, TgPrP(S1)-17, TgPrP(S1)-19 and TgPrP(S1)-39 lines plotted to a different regression from mice expressing different levels of the WT PrP^C^ allele (*Prnp*^a^), while the TgPrP(S3.F88W)-14 and TgPrP(S3.F88W)-35 lines behaved in yet a third distinctive manner (Fig7B), indicating unique pathogenic mechanisms operating in mice expressing the S1 and S3 PrP alleles. In sum, apart from the signature of denatured/native ratio, our CDI data did not reveal any qualitative differences in the nature of PrP^Sc^ in TgPrP(S1) and TgPrP(S3.F88W) mice; instead, repeated measures revealed drops in the quantities of protease-sensitive PrP^Sc^, rPrP^Sc^, infectious titre and associated neuropathology. Lastly, lower prion titre by SSCA (Fig7A, *P* < 0.001) yet shorter incubation times (Table2, *P* = 2.44E-19 and 1.75E-18 in TgPrP(S3.F88W)-14 and TgPrP(S3.F88W)-35 mice, respectively) versus WT mice indicates an uncoupling effect such that these Tg animals respond to pathologic forms of PrP differently from their WT counterparts (Fig7C), as discussed below.

## Discussion

### β-processing of PrP^C^ directed by a variant octarepeat region

An unexpected result in our study of OR function was that the expression of mutant PrP associated with component 3 geometry (i.e. the S3 and S3F88W PrP alleles) in RK13 cells or Tg mice resulted in a ∽3.5-fold elevated level of a ∽20–21 kDa C2 fragment. A reciprocal trend for decreased C2 production by S1 alleles (Table1) did not reach significance. The increased C2 levels observed in our experiments cannot be attributed to differential turnover effects as S3 PrP and WT PrP have identical amino acid sequences for this fragment (see Fig1); instead, the effect is attributed to increased cleavage. Though Tg mice expressing S3 PrP have increased levels of full-length PrP compared to WT, this alone does not account for the increased levels of C2 fragment as uninfected tga20 mice overexpressing PrP six-fold do not over-produce the C2 fragment (Fischer *et al*, 1996; Westergard *et al*, 2011). While protease-sensitive C2 is present in uninfected cells (Mange *et al*, 2004) and accumulates in infected animals, there is comparatively little known about its genesis *in vivo*. With recombinant PrP (McMahon *et al*, 2001) and with additives to cultured cells (Watt *et al*, 2005), there is a relationship to reactive oxygen species, but our cumulative data indicate that increased C2 production from S3 alleles is unlikely to be a direct effect of Cu-binding within the OR, as suggested by previous *in vitro* studies (McMahon *et al*, 2001; Pushie & Vogel, 2008, 2009). The possibility that ablation of OR metal binding by HoctaY substitutions delegates activity to the adjacent metal-binding site, ‘site 5’, involving H95 and H110 residues (Jackson *et al*, 2001; Kramer *et al*, 2001; Qin *et al*, 2002) was excluded by a compound mutated allele composed of OR HoctaY + H95A, which still exhibited robust production of the C2 fragment (Fig2B).

Although prolines play a key role cuing the S3 PrP allele for C2 proteolysis (Figs1C and 2C), two observations argue that this effect does not arise simply from an enzymatic preference for these residues. First, while there is a CNS-expressed protease with a preference for cleaving after prolines, the nature of the beta-propeller structure guarding the active site in prolyl endopeptidase means (as its alternative name prolyl oligopeptidase suggests) that peptides are preferred over polypeptides as substrates (Szeltner & Polgar, 2008). Second, preliminary mass spectrometry studies of PrP^C^ immunoprecipitated from brain and cleaved chemically at Met128 reveal no prolines in positions immediately N-terminal to C2-like fragments. Instead, the PrP fragments with confident assignments are the same in TgPrP(S3.F88W) mice and Tga20 mice expressing WT PrP^C^ (G. Schmitt-Ulms, personal communication). Thus, S3 PrP is likely processed by the same protease that processes WT PrP^C^.

Enhanced C2 generation by the S3 allele appears to reflect a facet of N-terminal domain function that is not related to the nature of amino acid residue side chains, but that is impacted by the flexibility of the polypeptide backbone. Proline is a unique component of polypeptides with partial double-bond character in the carbon–nitrogen backbone (between the nitrogen of the ring structure and proceeding carbon atom with a carbonyl group) that restricts free rotation. While two GQ > PP substitutions in the S1 PrP allele had no effect upon C2 production (Figs3), single G > P substitutions in ORs 2–5, GGG -> GPG, appear crucial for scission of S3 PrP (Fig2B) and distinguish this effect functionally and structurally from a potential role of the adjacent tryptophan-glycine (WG) motifs, which may affect RNA processing events (Gibbings *et al*, 2012). The role of single proline residues in facilitating cleavage is exemplified by experiments to introduce GPG motifs one-by-one into the GGG sequences of octarepeats 5, 4, 3 and 2, which causes a ladder of increments in the length of the C2 fragment (Fig2C). As increased C2 production is not joined by a similar increase in C1 production, there is a consequent alteration in the C2:C1 ratio. Since TgPrP(S3.F88W) mice with altered C2:C1, in turn, behave in a singular manner for some biological endpoints (below), determining the exact mechanism of chain scission—presumably mediated by a protease with tissue-specific distribution—will be an important future goal. Furthermore, since C2 PrP contains the major binding site for oligomeric forms of Aβ (Lauren *et al*, 2009), β-processing of PrP may have ramifications that extend to AD pathogenesis.

Lastly, by way of clarification, supraendogenous levels of C2 PrP are observed in prion infections (Chen *et al*, 1995; Yadavalli *et al*, 2004). In this context, C2 PrP resembles the core of the infectious prion protein PrP27–30 that is PK resistant, while C2 derived from PrP^C^ remains PK sensitive (Mays *et al*, 2014) (Supplementary Table S1). This type of C2 PrP may arise from a processive protease activity often referred to as ‘N-terminal trimming’ and attributed, at least in cells, to the action of lysosomal proteases (Taraboulos *et al*, 1992). The Ca^2+^-activated non-lysosomal protease calpain has also been invoked for the production of this species (Yadavalli *et al*, 2004). Whether the ultimate effect of C2 PrP on infection differs depending on the mechanism of production remains to be determined.

### Prion replication and pathogenesis in TgPrP(S1) and TgPrP(S3.F88W) mice

Transgenic studies have shown an unambiguous requirement for PrP^C^ C-terminal sequences to support efficient prion propagation (Fischer *et al*, 1996; Supattapone *et al*, 1999; Flechsig *et al*, 2004), but there is a growing appreciation that the N-terminal natively unstructured region contains targeting signals that impact the eligibility of PrP^C^ substrates (Sunyach *et al*, 2003) and also undergoes *cis* interactions with distal sequences (Flechsig *et al*, 2000; Qin *et al*, 2000; Sonati *et al*, 2013; Spevacek *et al*, 2013). In earlier studies, deletions that remove all or part of the OR (PrPΔ32–80, PrPΔ69–84) were associated with a ten-fold drop in titre and a four-fold drop in protease-resistant PrP^Sc^ (Fischer *et al*, 1996). PrPΔ32–93 deletion alleles were associated with a 10–30× drop in titre and 30–50× drop in PrP^Sc^ precipitated with sodium phosphotungstate, and no overt CNS pathology (Flechsig *et al*, 2000). Studies described here also caused drops in levels of misfolded PrPs but did not perturb PrP residues 32–50 which contain a different type of motif, namely two hexarepeats (Flechsig *et al*, 2000). Instead, we used missense mutations restricted to the OR region (residues 50–90 in mouse) to gain insights into the function of this enigmatic domain within the context of full-length PrP^C^ molecules.

For both the S1 and S3 PrP alleles, with the single exception of altered denatured/native ratios for S3 (Supplementary Fig S12), we were unable to discern allelic differences in the quality of protease-resistant forms of PrP using gel electrophoresis, GdnHCl denaturation and sedimentation profiling of oligomeric species (Fig5, Supplementary Figs S9 and S11). Quantitative changes were apparent, however.

For S1 PrP, while expressed at higher than endogenous WT levels in both the TgPrP(S1)-17 and TgPrP(S1)-19 lines, PrP^Sc^ isoforms at disease endpoint (Table3, Supplementary Fig S11) and spot counts in SSCA (Fig7A) were depressed. We can conclude that S1 PrP is less efficient than WT PrP^C^ in the tempo and ceiling value for production of total PrP^Sc^, rPrP^Sc^ and prion titre. These data therefore extend earlier genetic studies that deleted the OR and flanking sequences (Fischer *et al*, 1996; Flechsig *et al*, 2000) by defining the competence for chain flexibility in the OR as a factor that allows for accumulation of prion titre, total PrP^Sc^ and rPrP^Sc^ to attain WT levels.

In the case of S3 PrP, the Tg mice expressing this allele succumbed to prion disease ∽60 days earlier than TgPrP(S1)-17 and TgPrP(S1)-19 mice. Two variables are relevant in drawing these comparisons: the levels of full-length PrP^C^ in uninfected TgPrP(S3.F88W)-14 and TgPrP(S3.F88W)-35 mice are increased 0.7× over TgPrP(S1)-17 and TgPrP(S1)-19 mice, while the ratio of C2 production changes by greater than three-fold (Table1). Regarding the former, it is well known that increasing full-length PrP^C^ above WT levels decreases disease duration (Carlson *et al*, 1994). In the case of mice expressing WT PrP at 3–4× levels, incubation times with the same RML prion isolate were 100 ± 17 days (Fischer *et al*, 1996) versus 89.1 ± 2.5 and 84.1 ± 2.6 days in the TgPrP(S3.F88W)-14 and TgPrP(S3.F88W)-35 lines with 3× expression, respectively (Tables1 and 2); these figures are in reasonable agreement given the margins of error in scoring the terminal phase of prion disease and could suggest that expression level is the key variable in arriving at incubation times < 100 days. However, quite curiously, S3 PrP alleles also have levels of pathogenesis-associated species that are below those of WT controls; lower spot counts in SSCA (Fig7A); a four-fold drop in rPrP^Sc^; and a > six-fold drop in protease-sensitive PrP^Sc^ isoforms at disease endpoint (Table3, Supplementary Fig S11). Our studies also found a prolongation of incubation time period upon passage through TgPrP(S3.F88W)-35 mice but the drop in infectious titre that did not reach significance (noting that a one log-unit drop defines a minimum for a significant change in titre determined by bioassay (Prusiner, 1987)). To resolve the paradox of lower amounts of pathogenic species yet abbreviated incubation times, we infer that C2 PrP—normally present at much lower levels than C1 PrP—may be important and have a greater ability to perform signalling from pathogenic forms of PrP (Fig7C). Future experiments with knock-in mice and *ex vivo* cultures may be of great use to tease apart the relationships between truncated PrP^C^ species and toxic signalling originating from PrP^Sc^ or Aβ oligomers.

### Octarepeat region binding partners and uncoupling of disease phenotypes

We redesigned the PrP OR with an expectation that biological properties of the conformationally locked S1 and S3 alleles would differ from WT PrP—this expectation was fulfilled for aspects of disease pathogenesis and for some aspects of a physiological function in maintaining myelination of peripheral nerves (Figs3, 4, 6 and 7), thus confirming a modulatory function for the OR. The effects ascertained in infected TgPrP(S1) and TgPrP(S3.F88W) mice are of special interest because uncoupling between neurological disease and accumulation of PrP^Sc^ has been seen previously, and at one stage, it was used to argue against the validity of the prion hypothesis that the infectious agent is composed of misfolded PrP (Czub *et al*, 1986; Lasmezas *et al*, 1997). While disease transmission from recombinant PrP molecules address the latter issue (Legname *et al*, 2004; Wang *et al*, 2010), data presented here now begin to suggest a mechanistic basis for how uncoupling might occur, namely by modulating OR rigidity.

From our studies using amino acid substitutions to perturb OR rigidity, we deduce that altered interactions with OR-binding co-factors that vary within the physiological environment will also modulate OR rigidity and consequently affect roles of the PrP linker region and globular domain in prion replication and pathogenic signalling from PrP^C^ (Brandner *et al*, 1996; Spevacek *et al*, 2013). While our initial focus has been copper binding, the WT OR can bind other species (Beland & Roucou, 2012). Binding partners with repetitive structures can make multiple contacts within the OR, and one molecule that holds the OR in an extended conformation, pentosan sulphate, is a potent inhibitor of prion accumulation (Taubner *et al*, 2010); in studies here, an extended conformation, ‘component 1’ geometry is inferred for the S1 PrP allele (Fig1B). In the case of the S3 PrP allele tailored for a compact OR conformation, the mechanism of pathogenesis may be different and might encompass altered susceptibility to β-cleavage occurring as a consequence of altered OR rigidity. In sum, the pleiotropic effects of proline substitutions that limit OR flexibility can be reconciled with this domain serving as a cell surface scaffold to bind diverse macromolecules and co-factors.

Beyond prion infections, the clinical onset of genetic prion diseases varies greatly, even when the effects of a common *PRNP* codon 129 polymorphism on the disease chromosome and on the non-mutated chromosome are excluded from the analyses. For example, corrected data on the P102L mutation in the PrP^C^ linker region define an average age of onset of 46.8 years with a standard deviation 12.4 years (Mead *et al*, 2006). Within an extended kindred with six extra octarepeats and early disease onset, the codon 129 genotype accounted for only 41% of the variance (Mead *et al*, 2006). Since our studies define OR flexibility as impacting diverse biological endpoints, we suggest it will prove to be an important variable in infectious disease paradigms where levels of PrP^Sc^ and infectivity appear dissociated from the onset of clinical disease. More generally, the OR can be likened to a clutch that controls the degree to which abnormally folded PrPs engage or disengage mechanisms to drive neurological disease. This action may also apply to familial prion diseases where a single mutant allele is capable of producing multiple clinical presentations.

## Materials and Methods

### Mutant PrP plasmid constructs

To create novel alleles in the PrP OR equivalent to the synthetic peptides (sequences available in Supplementary Materials and Methods) examined by electron paramagnetic resonance (EPR) spectroscopy (Supplementary Fig S1), the sequences encoding the N-terminal portion (with the mutations included) of PrP and part of the 5' UTR were synthesized by GenScript Inc. The cassettes encoding the OR-encoding sequences of interest were excised from the plasmid provided by GenScript using *KpnI* (New England Biolabs), annealed into the full-length PrP sequences in an existing pCDNA3 plasmid using T4 DNA ligase (New England Biolabs), and correct full-length PrP sequences were then moved into another vector pBudgfp by digestion with *XbaI* and *HindIII* (New England Biolabs), followed by gel extraction and ligation. For phenylalanine substitutions into the hydrophobic domain, proline substitutions into the octarepeats (G62P, G70P, G78P, G86P) and H95A mutations, primers were designed as per the GeneTailor Site-Directed Mutagenesis kit (Invitrogen) and the mutagenesis reaction was carried out as per the manufacturer's protocol. One microlitre of each reaction was transformed into competent DH5α cells (Invitrogen), and plasmids were isolated using a miniprep kit (Qiagen). Correct plasmids were confirmed by sequencing.

### Cell culture

Rabbit kidney epithelial (RK13), neuroblastoma (N2a), cured SMB (SMB-PS), human kidney epithelial (HEK) and human neuroblastoma (SH-SY5Y) cells were maintained in Dulbecco's modified Eagle's medium (DMEM; Invitrogen) supplemented with 10% foetal bovine serum (FBS; Invitrogen) and penicillin/streptomycin (Invitrogen) at 37°C and 5% CO_2_ levels. Cells of approximately 90–100% confluency were transfected using Lipofectamine 2000 (Invitrogen), according to the manufacturer's instructions. RK13 cell lines stably expressing wild-type (WT) PrP (WT-10) and mutant S1 (S1–29), S3 (S3–27) and S3.F88W (F88W-5) PrP were created by transfection with plasmids encoding the proteins, and clones were selected using zeocin (Invitrogen). Clones isolated with cloning rings (Scienceware) were amplified, lysed with RIPA buffer (50 mM Tris–HCl pH 7.5, 150 mM NaCl, 0.5% sodium deoxycholate, 1% NP-40) and analysed by Western blot for PrP expression.

### Transgenic mouse lines

For the generation of transgenic mice, sequences encoding WT PrP and mutant S1 and S3.F88W PrP were inserted into the MoPrP.*Xho* ‘half-genomic’ vector (Borchelt *et al*, 1996; Fischer *et al*, 1996). Mutant PrP sequences were extracted from the mutant PrP plasmids described above using the primers 5′AAAAACTCGAGGCCCTCATCCCACGATCAGG3′ and 5′AAAAACTCGAGAGTCCAATTTAGGAGAGCCAAG3′, which contain *XhoI* restriction enzyme sites. Polymerase chain reaction (PCR) fragments were cloned into pCR2.1.TOPO (Invitrogen), and sequences were verified. DNA from TOPO clones was digested using *XhoI* (New England Biolabs), isolated by gel electrophoresis and purified with a gel extraction kit (Qiagen). The MoPrP.*Xho* vector was digested with *XhoI*, treated with calf intestinal alkaline phosphatase (New England Biolabs), phenol:chloroform-extracted and ethanol-precipitated or purified with the Ultraclean Gel Spin DNA Purification Kit (MoBio Laboratories). After ligation of digested fragments, the sequenced DNA was amplified and purified using the Endo-Free Maxiprep Kit (Qiagen). To prepare for injection, the DNA was digested with *NotI* and purified using the gel extraction kit (Qiagen) or Ultraclean Gel Spin DNA Purification Kit (MoBio Laboratories). The DNA was then injected into the pronuclei of mouse FVB embryos by the University of Calgary Transgenic Services.

### Tissue homogenate preparation, gradient fractionation, CDI and SSCA

Ten per cent homogenates (w/v) of brain, spleen, heart, kidney, liver and lung were prepared in PBS complemented with Complete Mini Protease Inhibitor (Roche) using a homogenizer (VWR, model VDI 12) at 4°C. Sucrose gradient and the conformation-dependent immunoassay (CDI) were performed as previously described (Kim *et al*, 2012). Briefly, the 400-μl aliquots of 10% brain homogenate in PBS, pH 7.4, containing 2% sarkosyl were clarified by centrifugation at 500 *g* for 5 min and carefully layered onto the top of the 10% to 45% sucrose gradient prepared in PBS, pH 7.4, containing 1% sarkosyl. Ultracentrifugation was performed at 237,000 *g* for 73 min at 5°C in an Optima TL ultracentrifuge (Beckman) equipped with a Beckman SW 55 Ti rotor. After the centrifugation, fractions were collected from the bottom of the tube. The CDI assay was similar to previous descriptions (Safar *et al*, 2005; Choi *et al*, 2009) albeit with incorporation of several recent modifications (Mays *et al*, 2014). The standard scrapie cell assay (SSCA) was performed as described using L929 cells (Mays *et al*, 2014).

### Western blotting

Samples were run on 12% or 14% Tris-glycine gels using a Bio-Rad system and transferred to polyvinyl difluoride (PVDF; Millipore) membranes. Blots were immediately incubated with PrP antibody Sha31 (Spi-Bio Inc; 1/30,000 in 0.5% TBST) or were blocked with 5% skim milk in 0.1% TBST for 1 h at room temperature and incubated with primary antibody at 4°C overnight (PrP antibodies: 9A2 1/4,000, 12B2 1/8,500, SAF83 (Cayman), VRQ61 1/5,000, and 2D6 1/10,000, 1A6 1/5,000). 9A2 and 12B2 were gifts from Dr. Jan Langeveld (Langeveld *et al*, 2006), VRQ61 and 2D6 from Dr. Human Rezaei (Moudjou *et al*, 2004), and 1A6 was produced in-house. Membranes were subsequently incubated with secondary antibody at 1/10,000 (Bio-Rad) for 2 h at room temperature and visualized using ECL (Pierce). After stripping, membranes were incubated in anti-actin primary antibody (Sigma; 1/5,000).

### Proteinase K and PNGaseF digestion

Samples were treated with 50 μg/ml of proteinase K (PK; Invitrogen) for 1 h at 37°C. The reaction was stopped with 2 mM phenylmethanesulfonyl fluoride (PMSF) and centrifuged at 13,000 rpm for 1 h at 4°C. The pellets were resuspended in 15 μl PBS and 3 μl of 6× SDS loading buffer was added before boiling. If samples were to undergo further PNGaseF digestion, the pellets were resuspended in 15 μl of 2% SDS and heated at 100°C for 10 min. Two microlitres of 10% NP-40, 2 μl of 10× G7 Reaction Buffer and 1 μl of PNGaseF were added, and the reaction was incubated at 37°C for 2 h. The reaction was stopped by adding 6× SDS loading dye and boiling. Samples not PK digested were digested by PNGaseF according to the manufacturer's protocol, overnight at 37°C with 0.2 μl of PNGaseF.

### Guanidine hydrochloride denaturation of PrP^Sc^

The 10% brain homogenate was centrifuged at 700* g* for 5 min, and the supernatant was collected. Equal amounts of brain homogenate were added to increasing concentrations of guanidine hydrochloride (GdnHCl) (Sigma), and the samples were shaken at 800 rpm for 2 h at room temperature. The concentration of GdnHCl was diluted to 0.4 M using RIPA buffer, and the total volume of each sample was made equal by adding 0.4 M GdnHCl. Samples were treated with 50 μg/ml PK for 1 h at 37°C, and the reaction was stopped using PMSF (1 mM final concentration). The samples were centrifuged at 100,000 *g* at 4°C, the pellet was resuspended in PBS, and 6× SDS loading buffer was added.

### Mouse inoculations, scoring and sample collection

Animals were housed in ventilated cages with a 12-h light/dark cycle and were given *ad libitum* access to food and water. Male and female FVB mice 4–7 weeks of age were randomly assigned for inoculation with RML (source S. Prusiner), natural scrapie from a Canadian ARQ/ARQ sheep (CFIA, Nepean, ON), classical BSE from a Canadian animal (CFIA, Nepean, ON), or uninfected WT mouse brain homogenate (control). Approximate sample sizes for each group were determined by previous inoculation experiments in the literature. Thirty microlitres of 1% brain homogenate in PBS was injected using a 25-gauge needle into the parietal lobe of each mouse. The inoculum was heated for 20 min at 80°C before inoculations were performed. After inoculations, mice were checked for symptoms of prion disease, including head tilt, ataxia, extension, clasp, weight loss and stupor; initial diagnosis of clinical disease onset was performed by animal staff performing health monitoring. Animals euthanized due to non-prion disease-related illness or injury were excluded from analyses. Animal experiments were performed in accordance with the Animal Care Committee of the University of Alberta, protocols AUP00000356 and AUP00000357.

### Immunohistochemistry and image analysis

Brains were fixed in 10% formalin for 48 h and were subsequently paraffin-embedded. Sections 4.5–6 μm thick underwent standard de-paraffinization and hydration, were treated with 4 M guanidine thiocyanate and probed for PrP^Sc^ using SAF83 antibody. Glutaraldehyde-fixed samples of sciatic nerve were processed as described previously (Westaway *et al*, 1994), with bright-field images of nerve cross sections captured with a 10× objective (Nikon Eclipse 90i microscope). GE Healthcare Investigator software was used to analyse individual nerves for area, circumference and fibre count (see Supplementary Materials and Methods).

### Electron microscopy and toluidine staining

Sciatic nerves fixed in 2% glutaraldehyde/1% paraformaldehyde and post-fixed in 1% osmium tetroxide were Epon-embedded using routine procedures, and ultrathin sections were cut. Semi-thin sections (0.5 μM) were used for toluidine blue staining, and ultrathin sections were used for electron microscopy. The ultrathin sections were mounted on formvar-coated copper grids and contrasted with uranyl acetate and lead citrate. The samples were viewed in a Jeol JEM-2100 electron microscope at an acceleration voltage of 120 kV and a magnification range of 1,500× to 4,000×. Electron micrographs were recorded on a Gatan Erlangshen ES500W CCD camera.

### Statistics

Normal distribution of data was confirmed by a Kolmogorov–Smirnov test, and unpaired, two-tailed Student's *t*-tests were used for comparisons of these sample groups with no *a priori* predictions for the performance of the PrP alleles. For incubation time data with *n* < 6 where normal distribution could not be confirmed, we used a Mann–Whitney test. Statistical tests were performed using InStat and GraphPad Prism software with *P*-values < 0.01; < 0.001 and < 0.0001 represented by 2, 3 or 4 asterisks, respectively. Exact *P*-values are reported in the main text or the figure legends and were calculated using GraphPad Prism and Microsoft Excel.

## For more information

http://www.cjdfoundation.org

http://www.prioninstitute.ca

## Supporting Information

Source Data for Supplementary Figure S9

Review Process File
