# Supplementary figures and images for: Octarepeat region flexibility impacts prion function, endoproteolysis and disease manifestation
Source: EMBO Mol Med. 2015 Feb 6;7(3):339–56. doi: 10.15252/emmm.201404588 (PMC4364950; doi:10.15252/emmm.201404588)

**Figure S1**

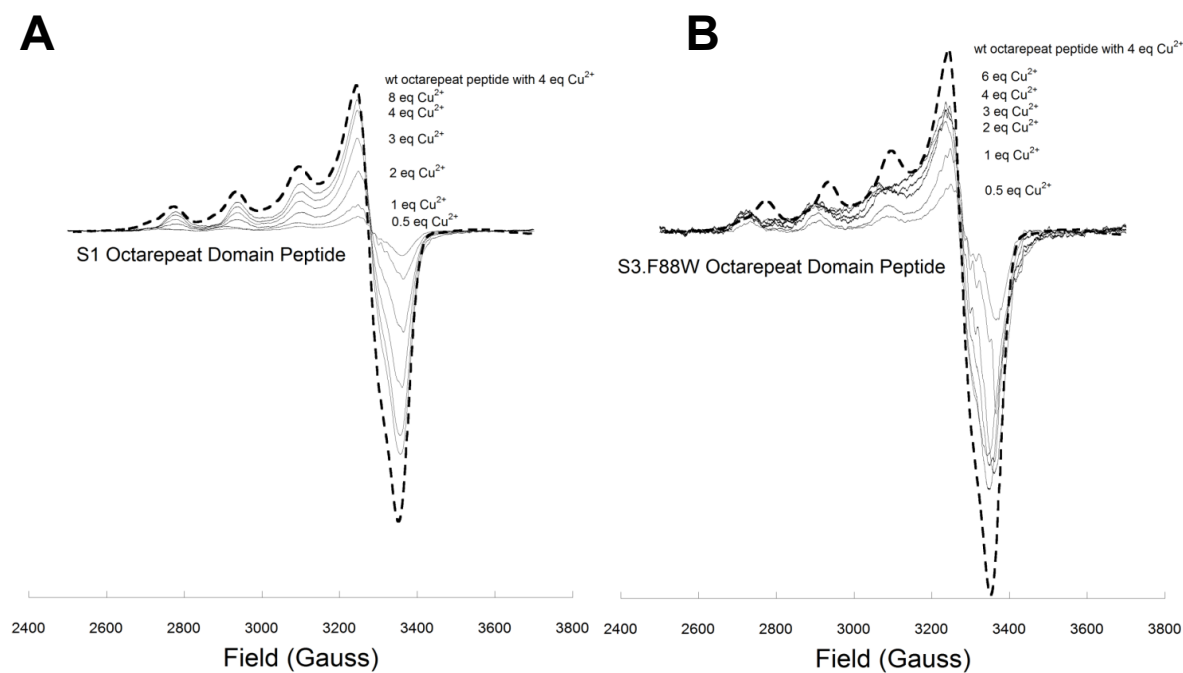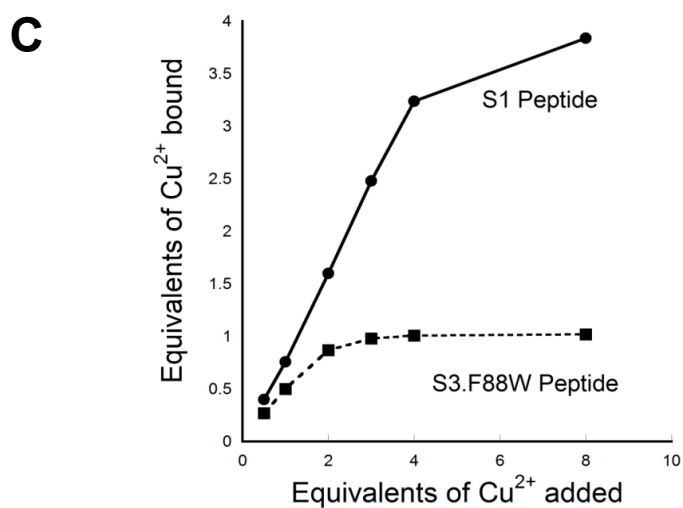

Supplement: Supplementary file 1 [file emmm0007-0339-sd1.pdf]

Figure S2

**A**

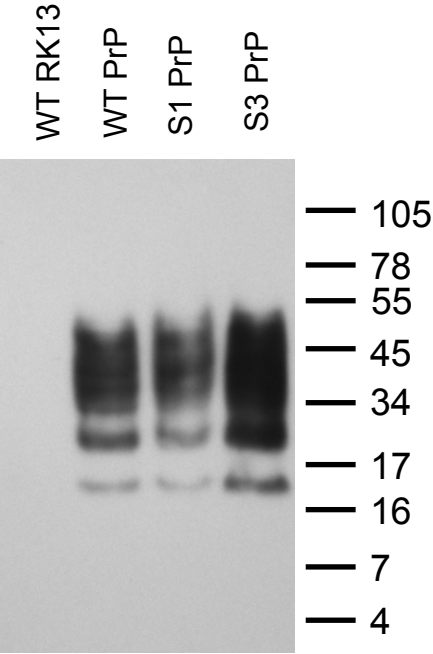

**B**

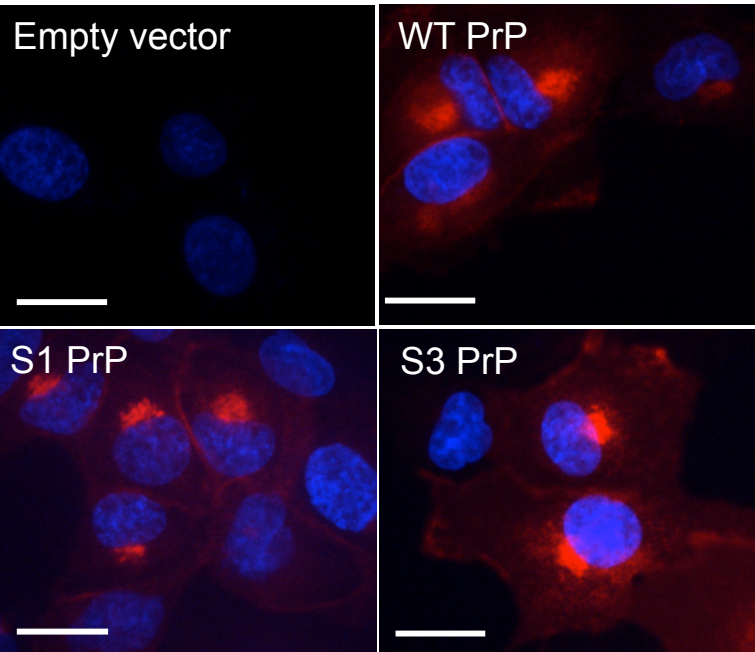

**C**

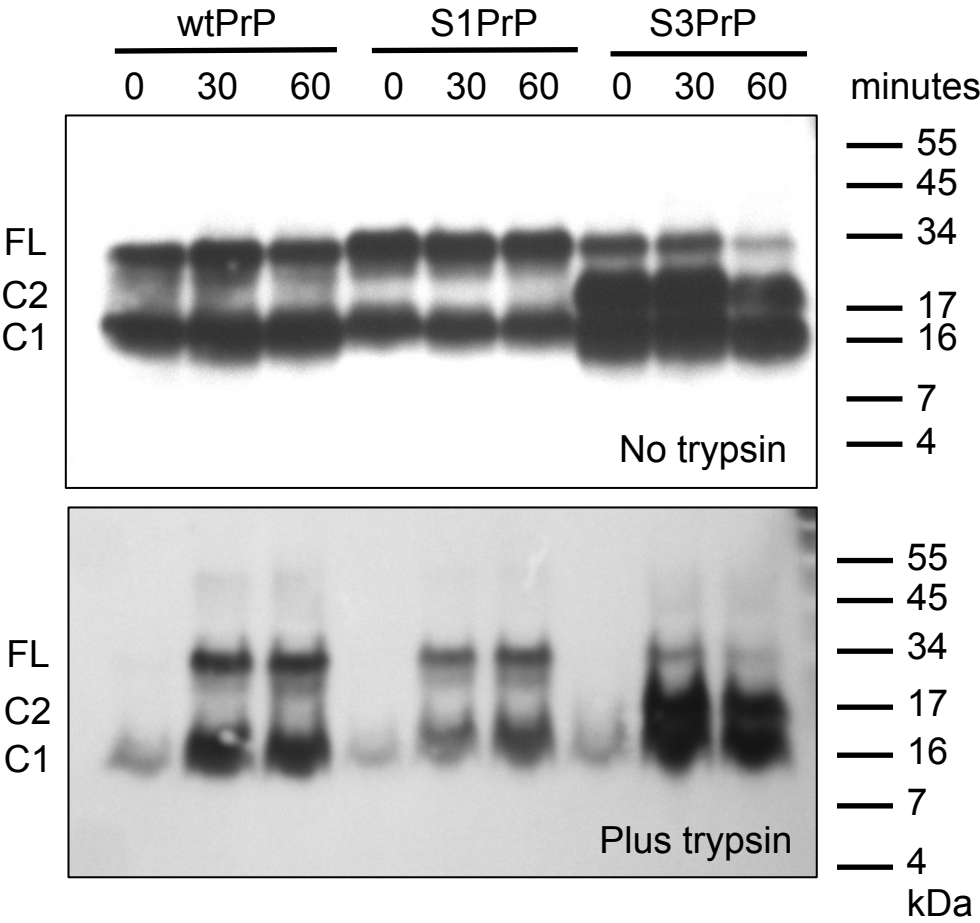

Supplement: Supplementary file 2 [file emmm0007-0339-sd2.pdf]

**Figure S3**

**A**

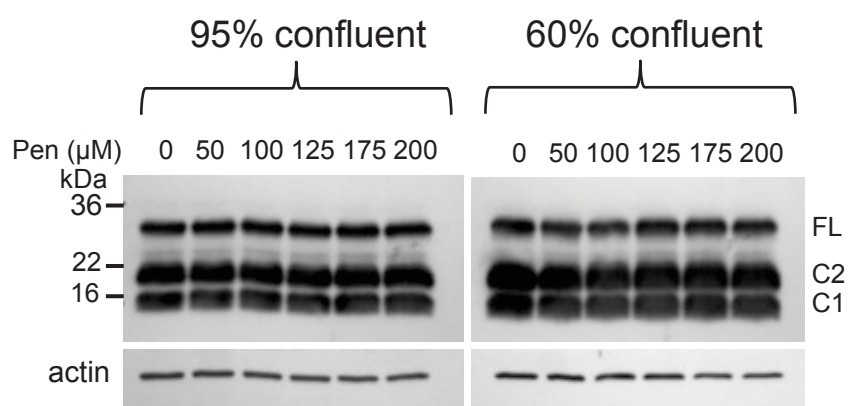

**B**

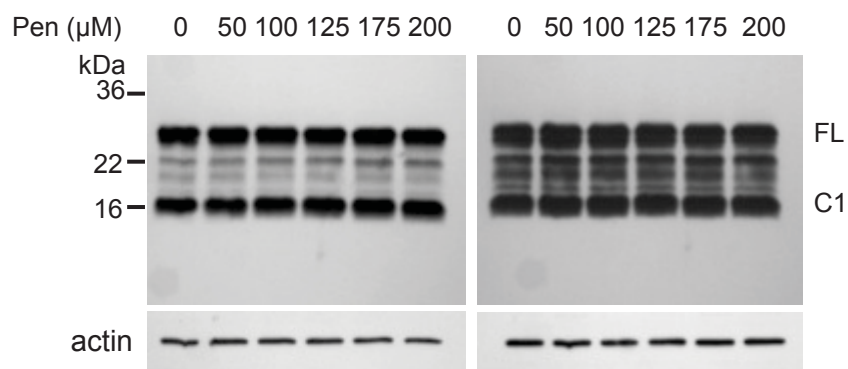

Supplement: Supplementary file 3 [file emmm0007-0339-sd3.pdf]

**Figure S4**

**A**

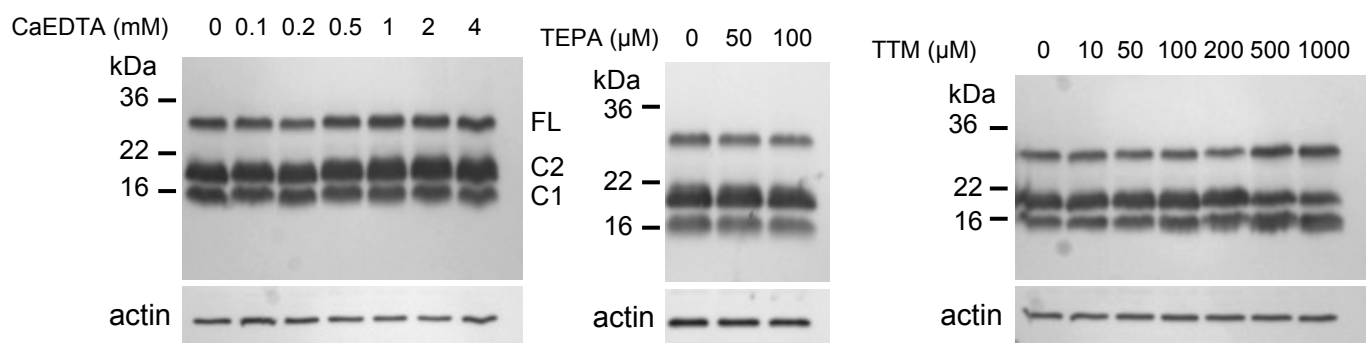

**B**

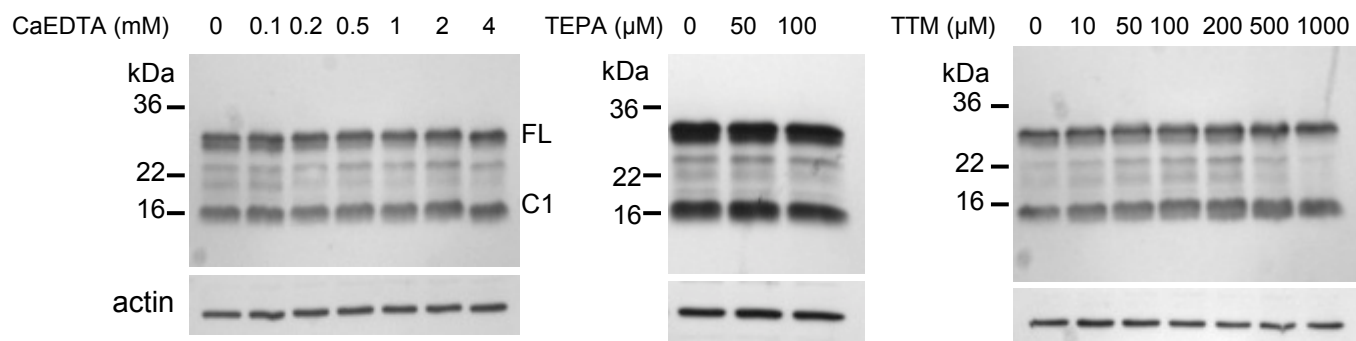

Supplement: Supplementary file 4 [file emmm0007-0339-sd4.pdf]

**Figure S5**

**A**

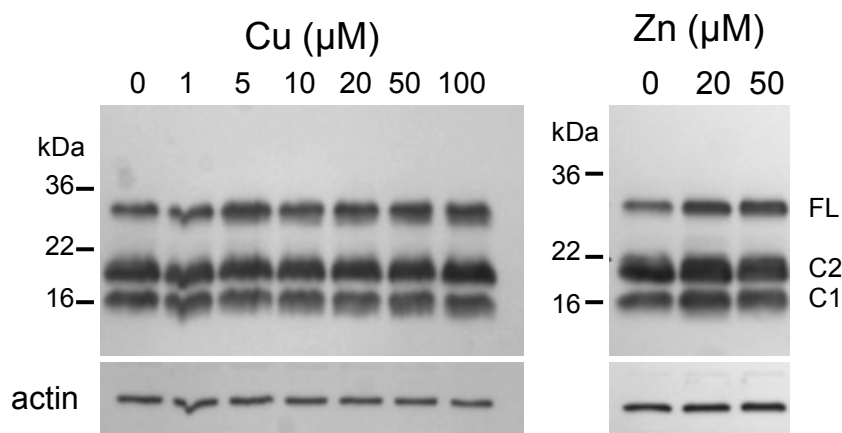

**B**

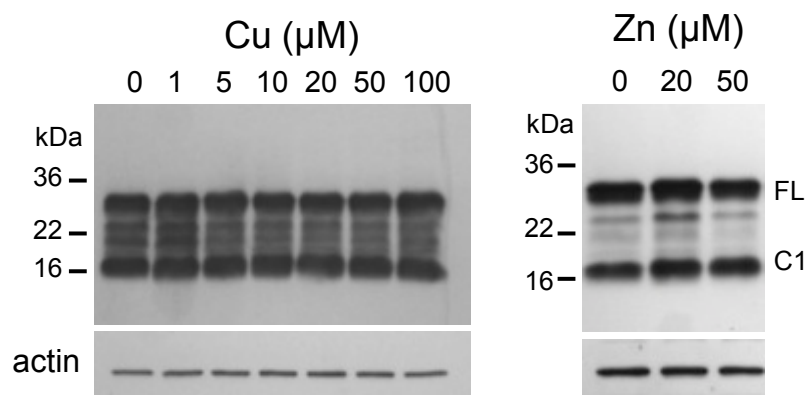

Supplement: Supplementary file 5 [file emmm0007-0339-sd5.pdf]

**Figure S6**

**A**

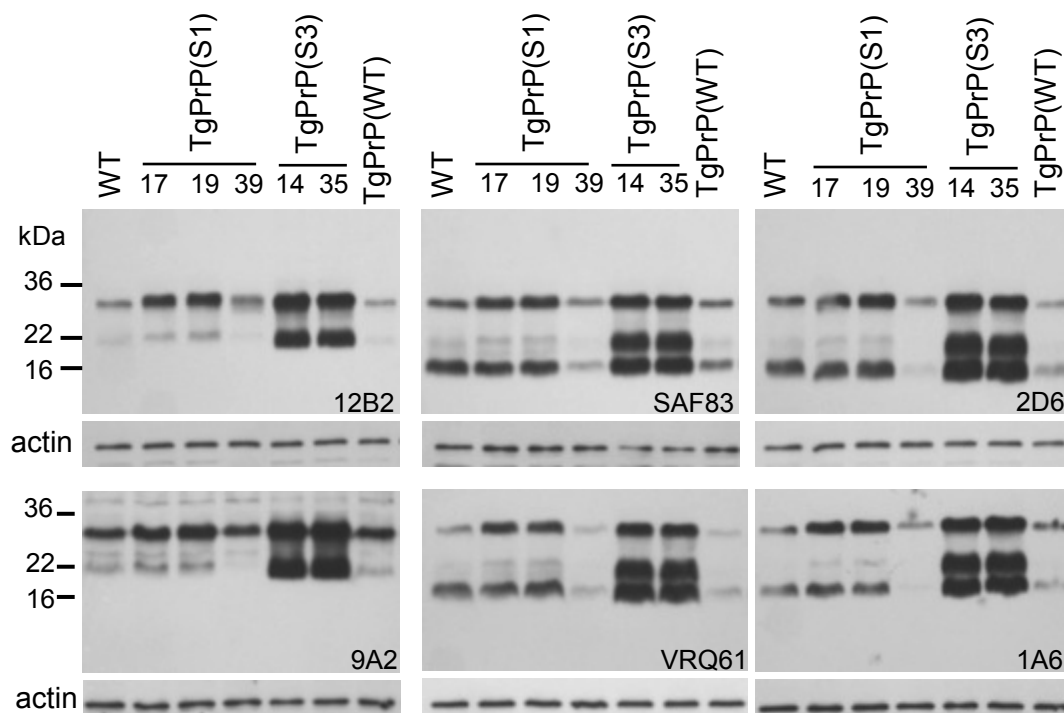

**B**

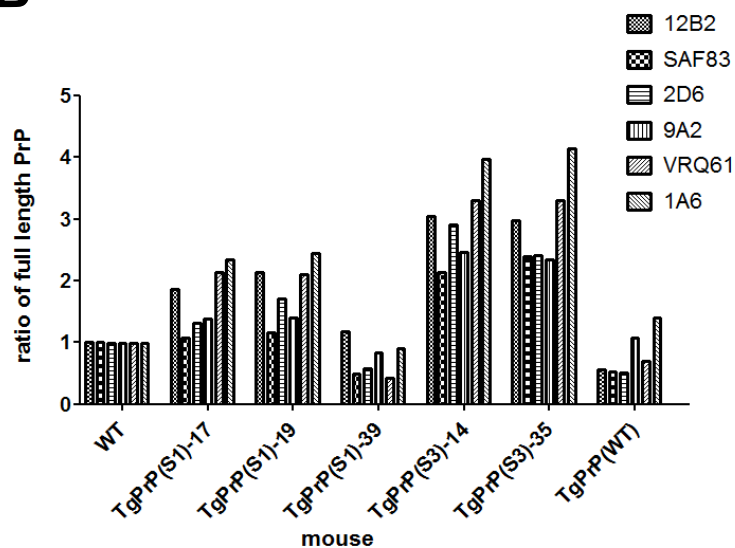

**C**

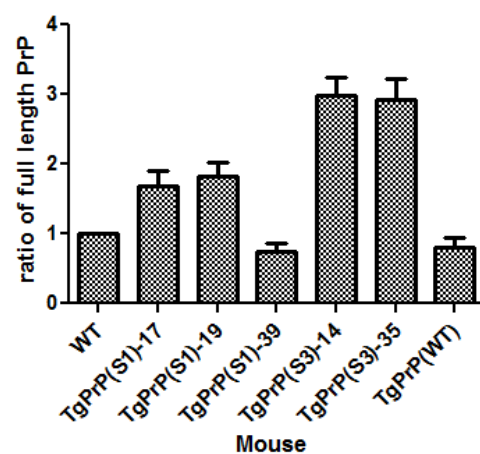

Supplement: Supplementary file 6 [file emmm0007-0339-sd6.pdf]

**Figure S7**

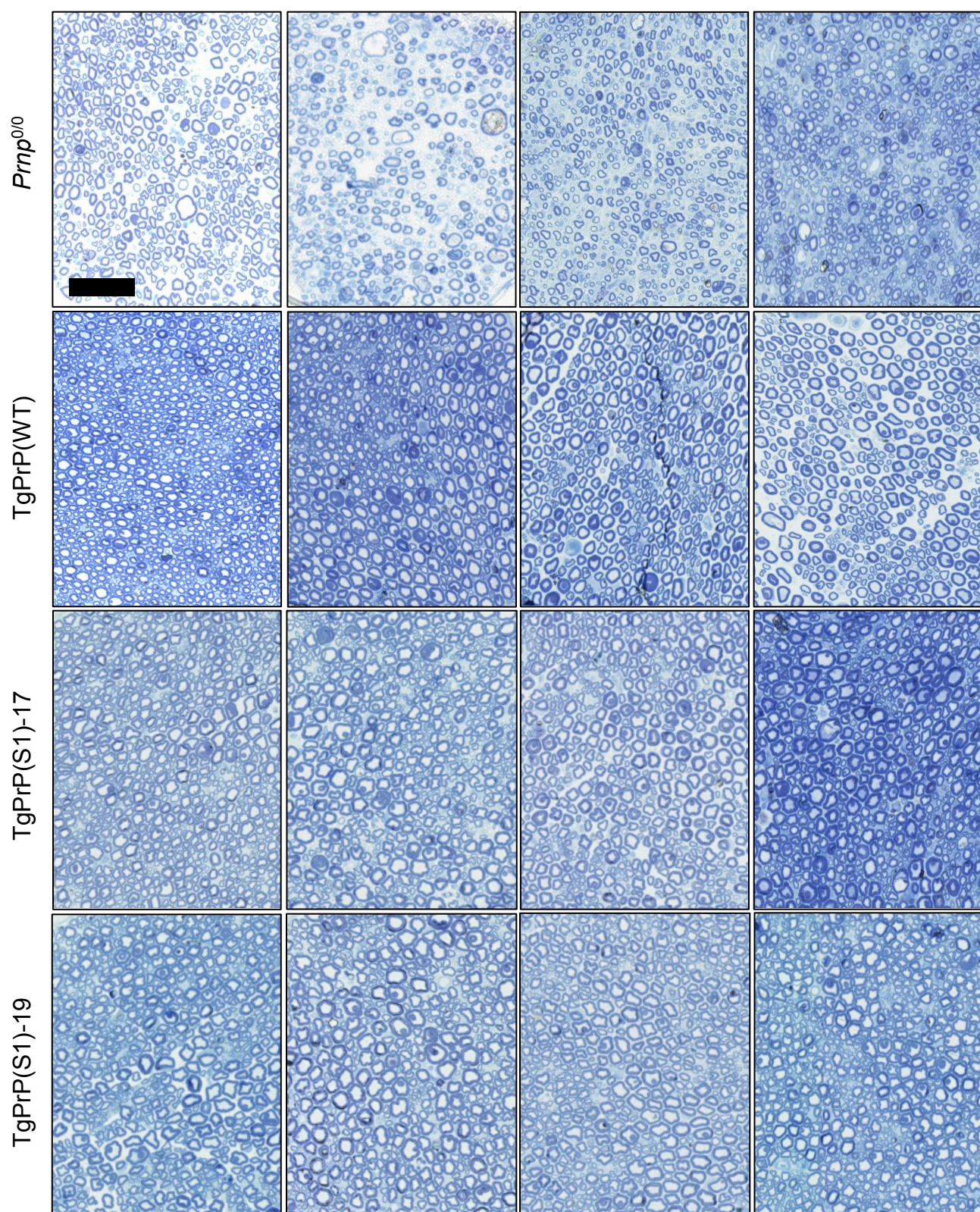

Supplement: Supplementary file 7 [file emmm0007-0339-sd7.pdf]

**Figure S8**

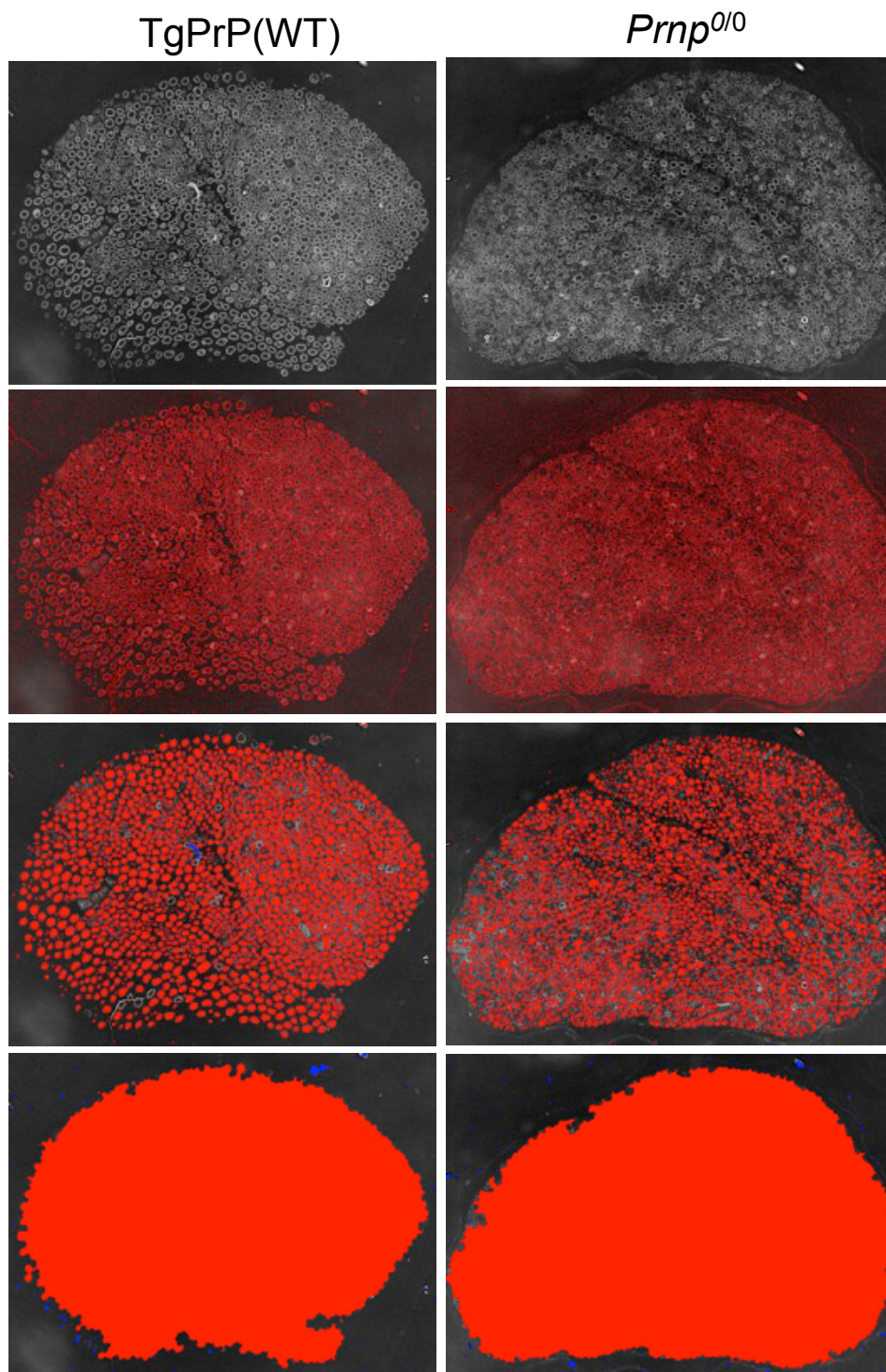

Supplement: Supplementary file 8 [file emmm0007-0339-sd8.pdf]

**Figure S9**

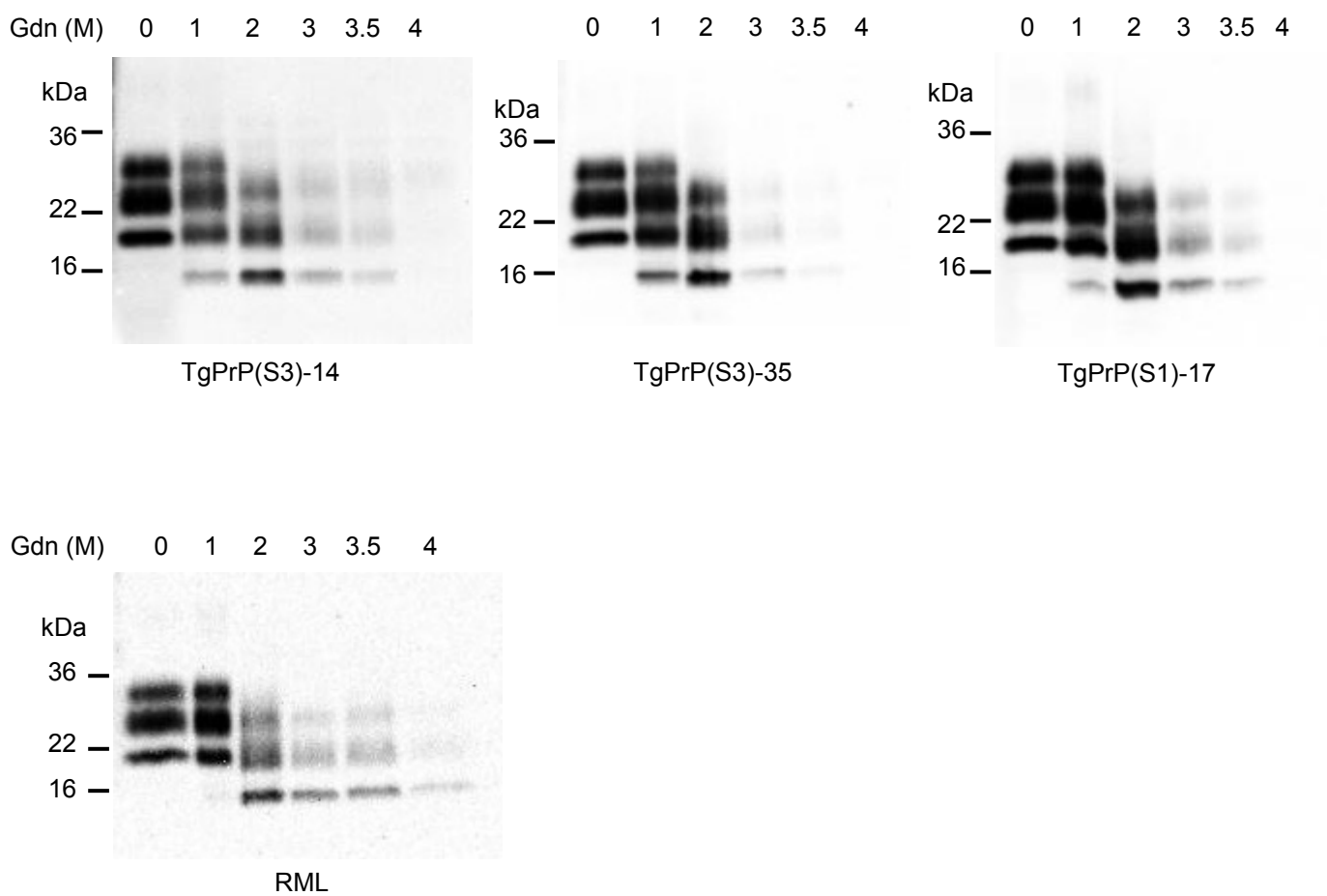

Supplement: Supplementary file 9 [file emmm0007-0339-sd9.pdf]

**Figure S10**

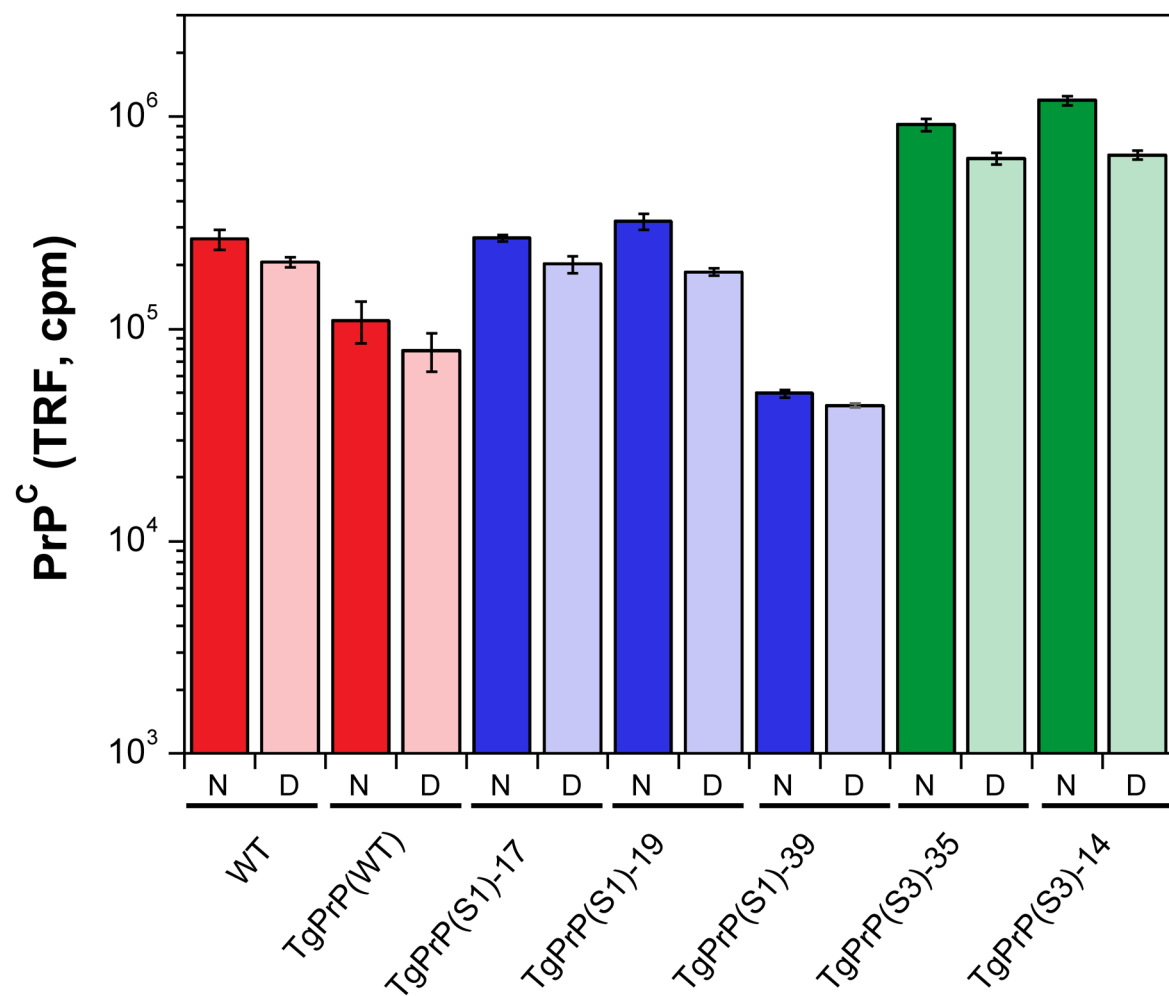

Supplement: Supplementary file 10 [file emmm0007-0339-sd10.pdf]

# Figure S11

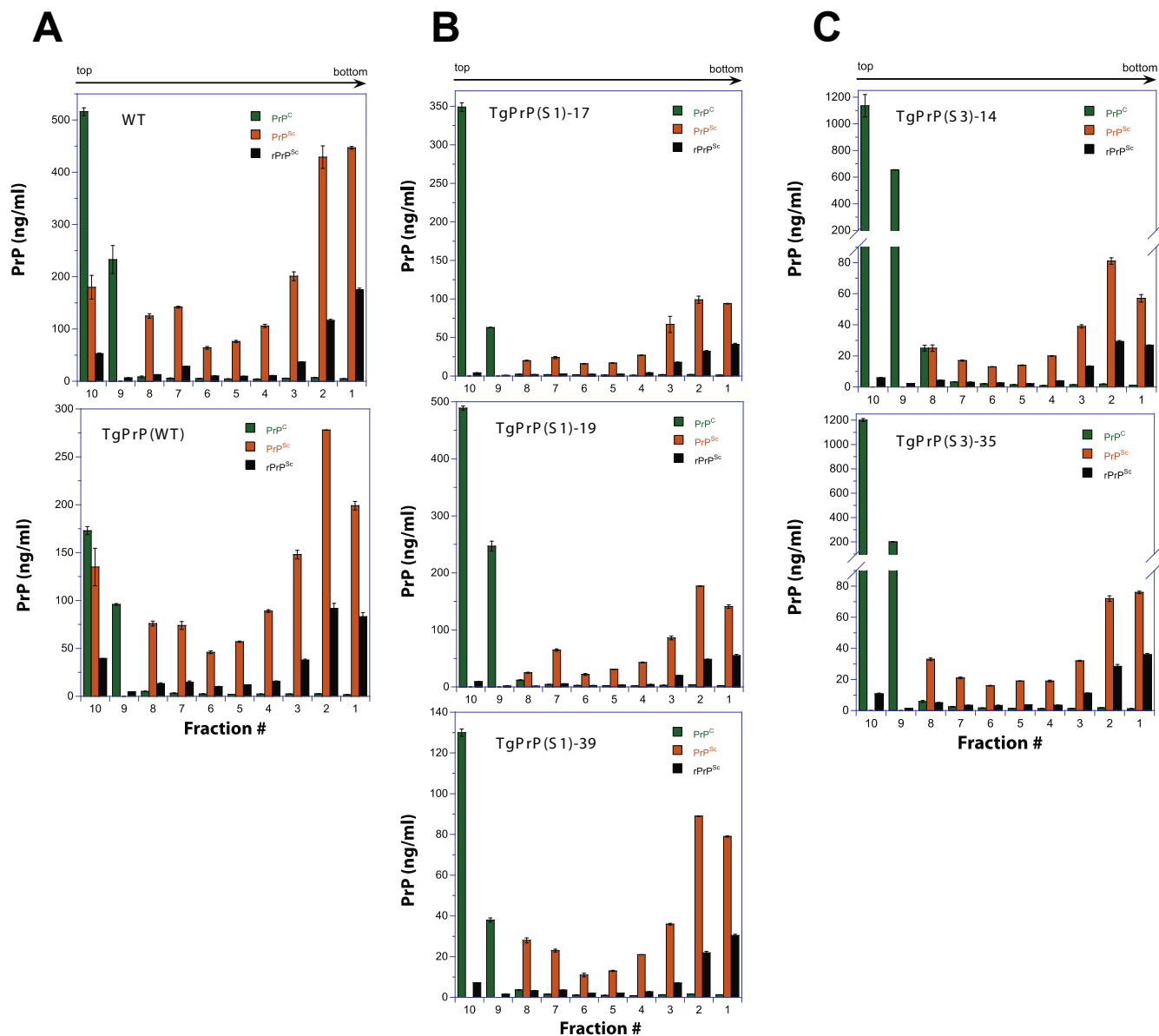

Supplement: Supplementary file 11 [file emmm0007-0339-sd11.pdf]

**Figure S12**

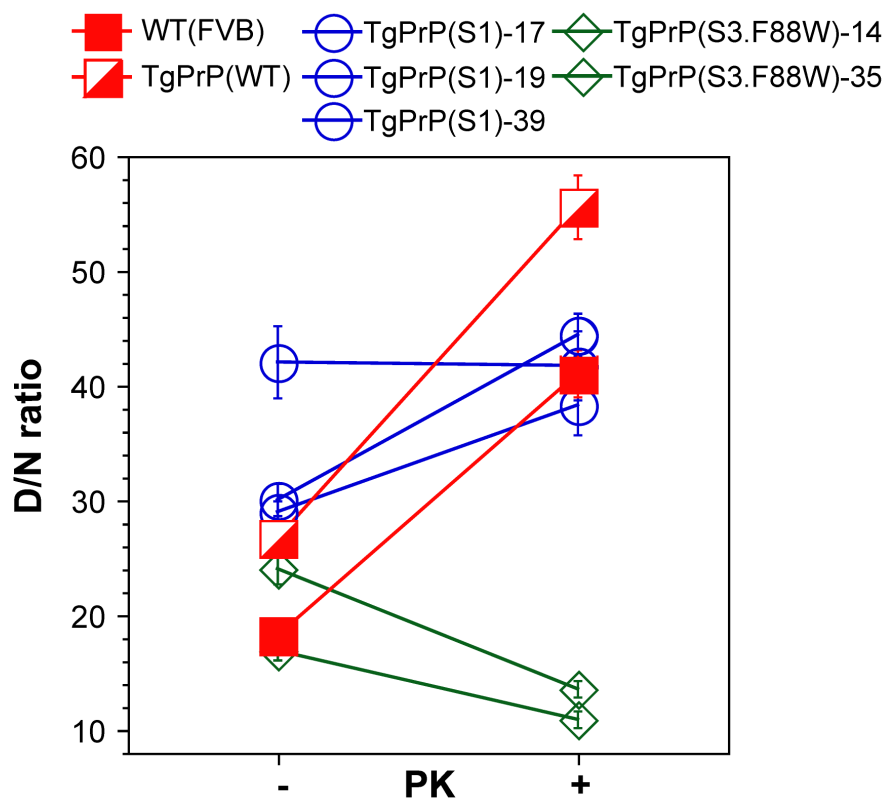

Supplement: Supplementary file 12 [file emmm0007-0339-sd12.pdf]

Figure S2

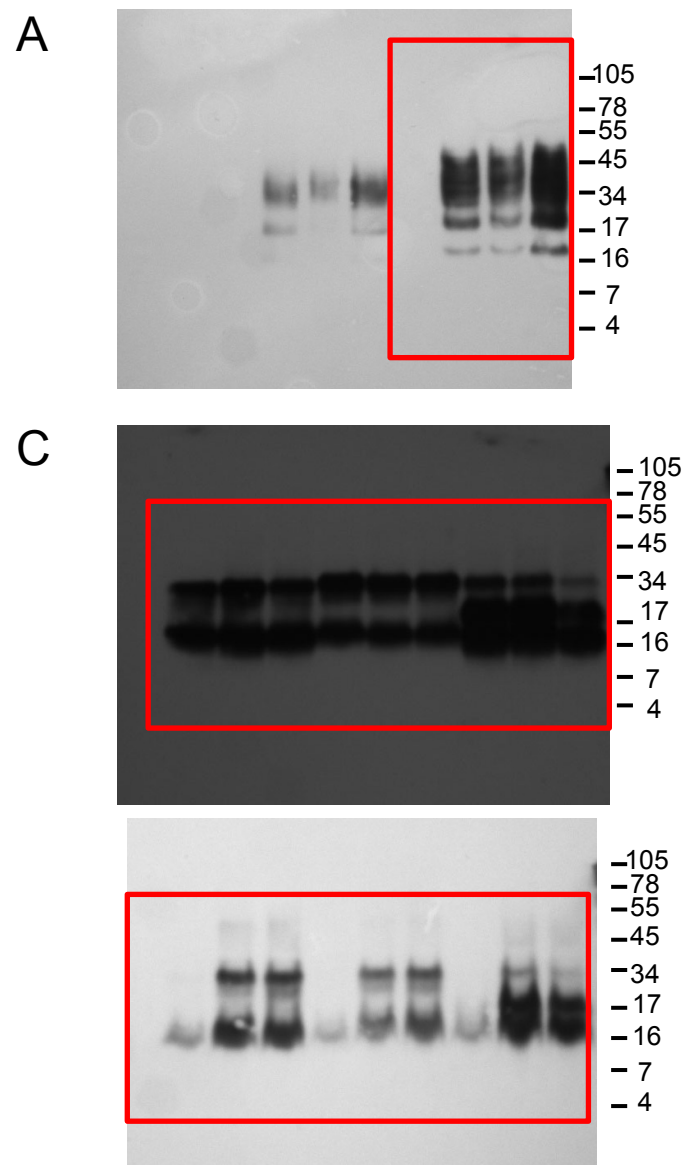

Supplement: Supplementary file 14 [file emmm0007-0339-sd14.pdf]

Figure S3A

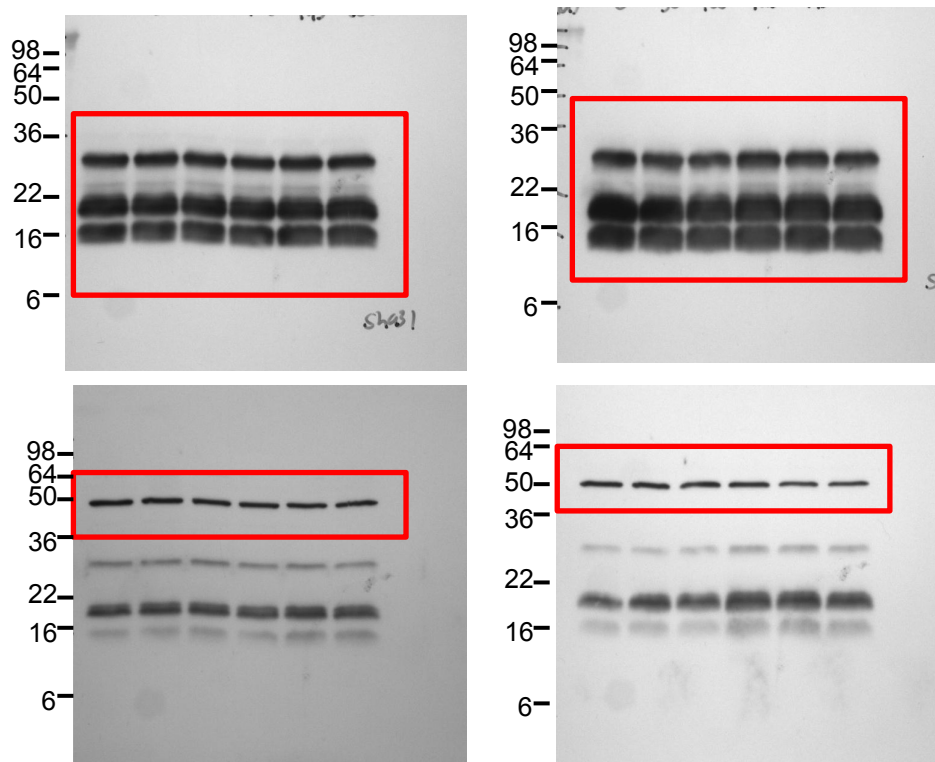

Figure S3B

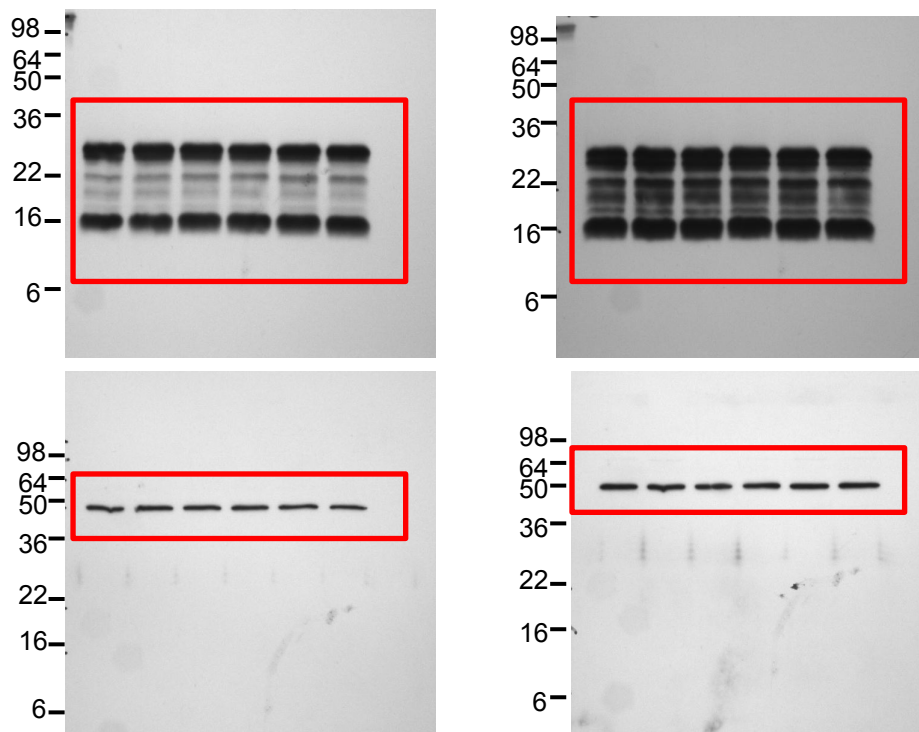

Supplement: Supplementary file 15 [file emmm0007-0339-sd15.pdf]

Figure S4A

CaEDTA

TEPA

TTM

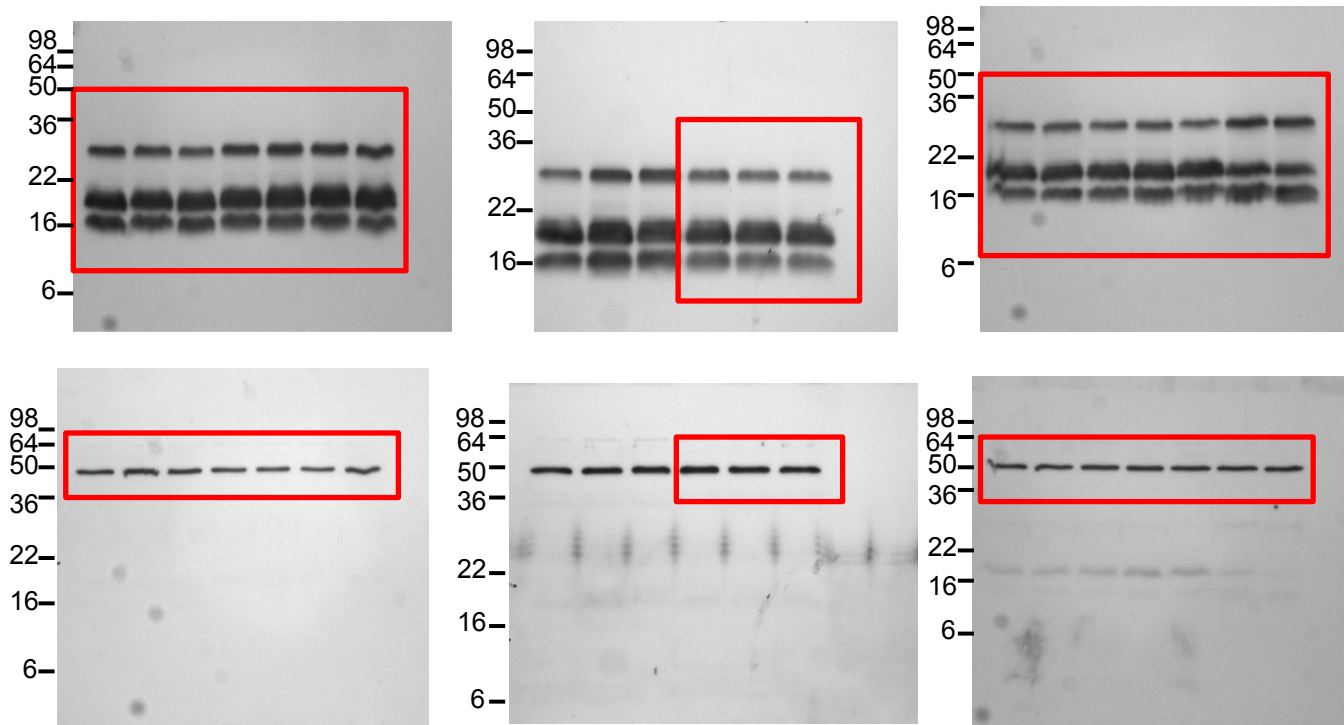

Figure S4B

CaEDTA

TEPA

TTM

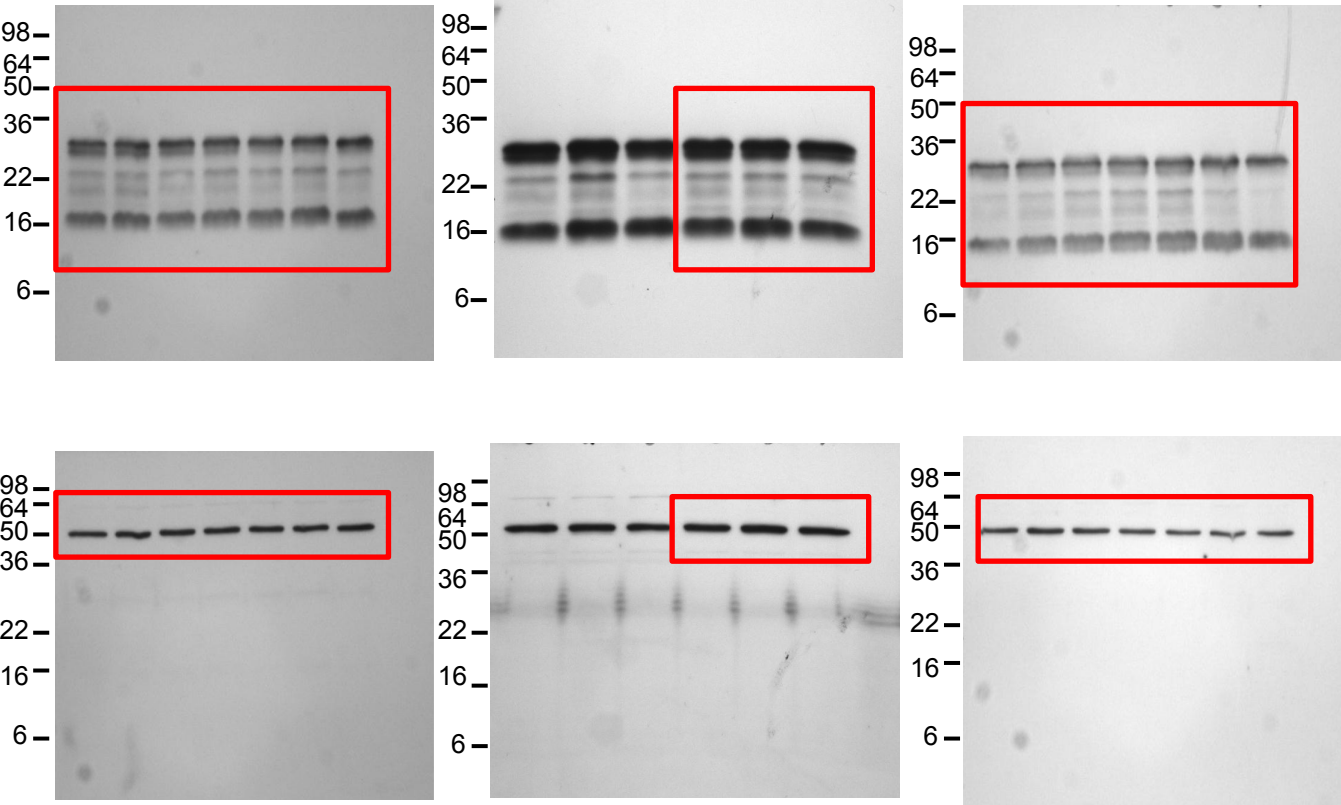

Supplement: Supplementary file 16 [file emmm0007-0339-sd16.pdf]

Figure S5A

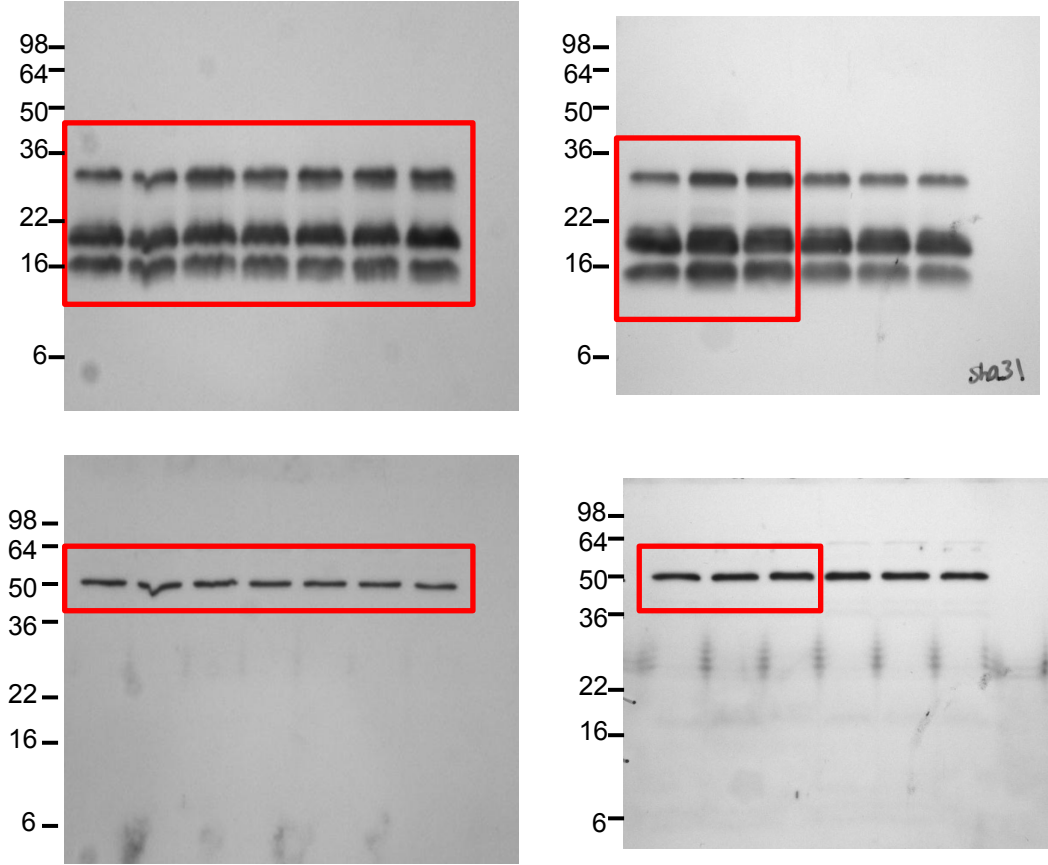

Figure S5B

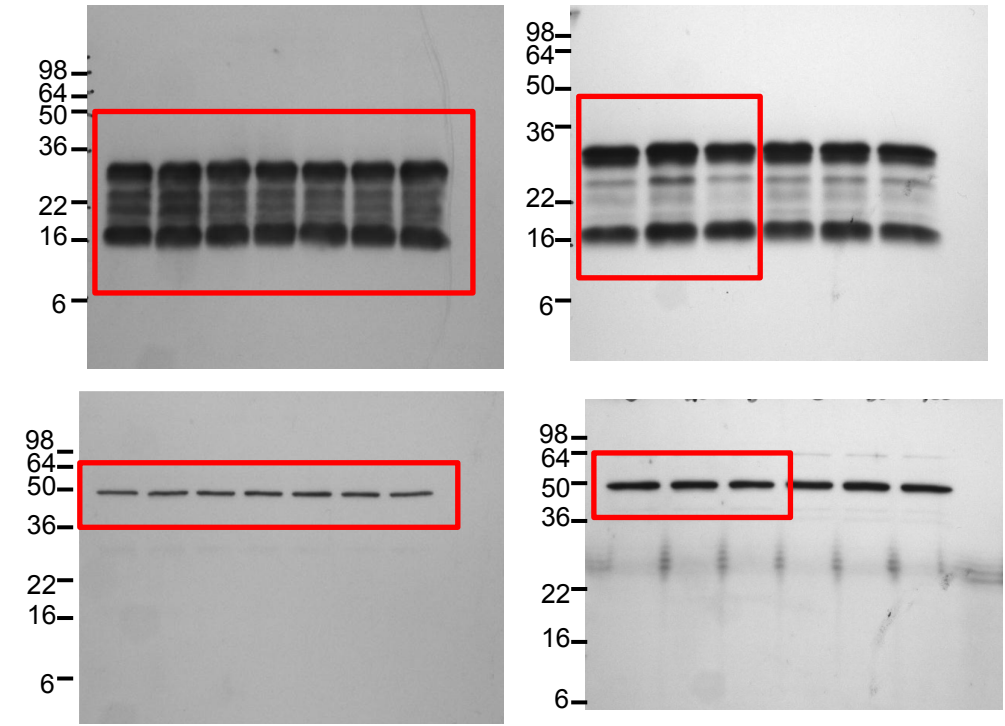

Supplement: Supplementary file 17 [file emmm0007-0339-sd17.pdf]

Figure S6A

12B2

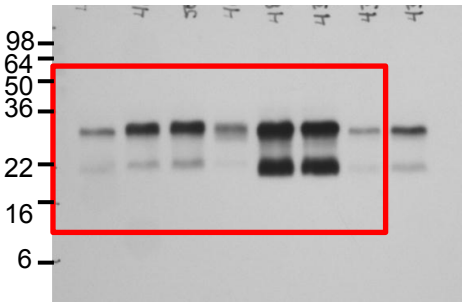

SAF83

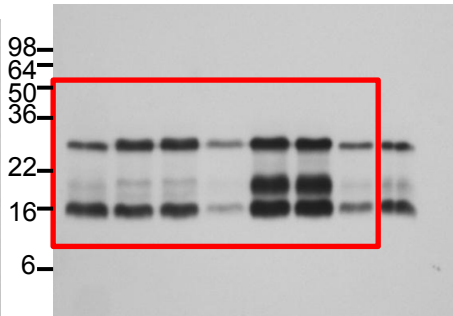

2D6

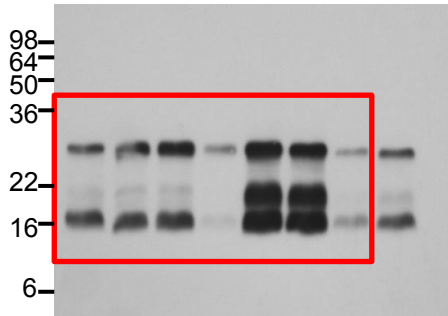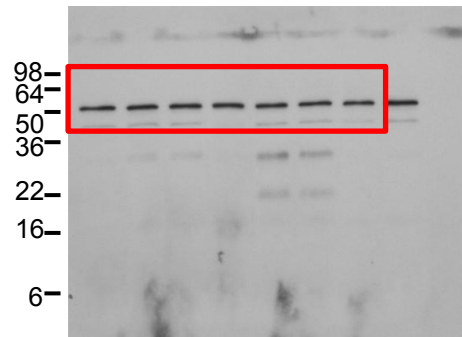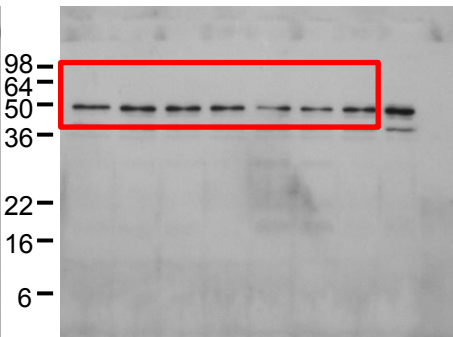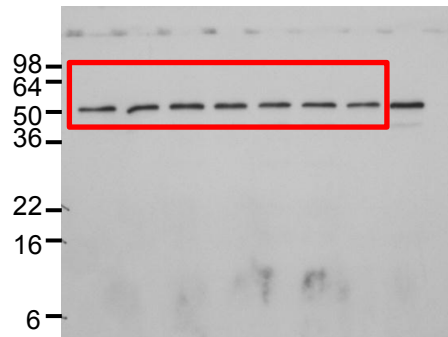

9A2

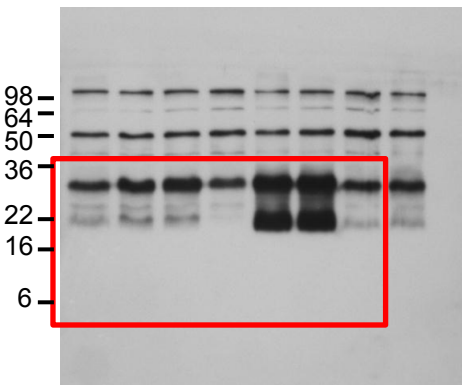

VRQ61

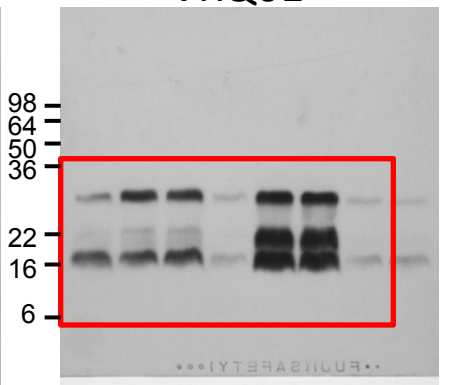

1A6

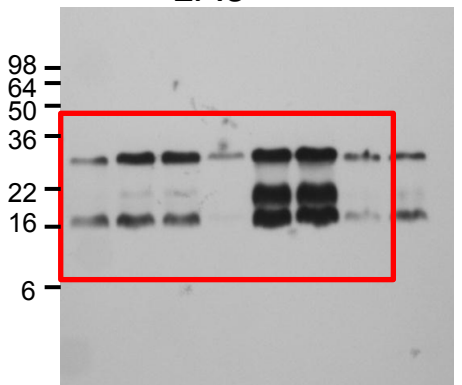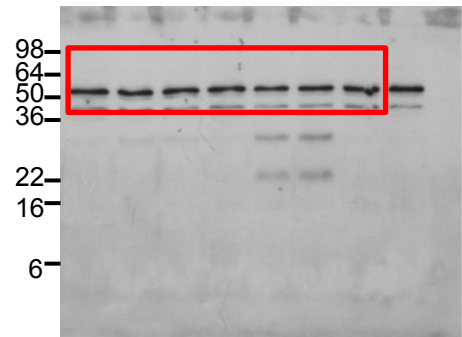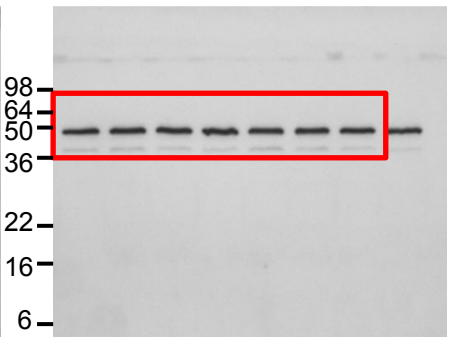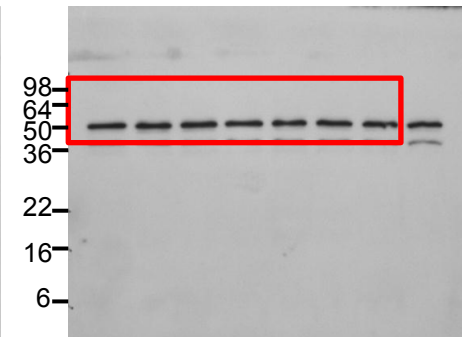

Supplement: Supplementary file 18 [file emmm0007-0339-sd18.pdf]

Figure S9

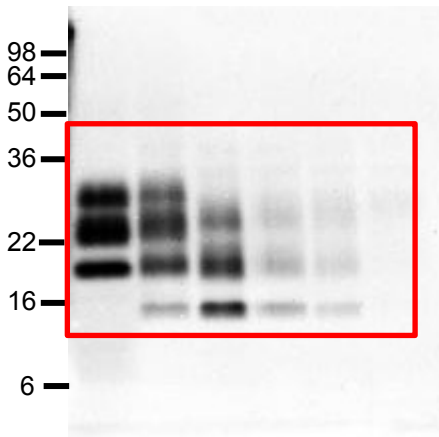

TgPrP(S3)-14

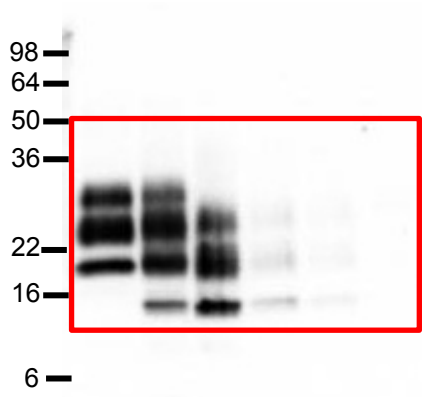

TgPrP(S3)-35

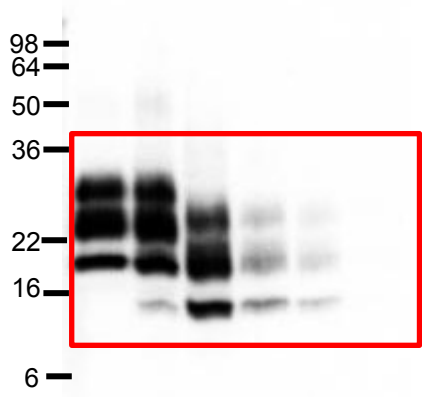

TgPrP(S1)-17

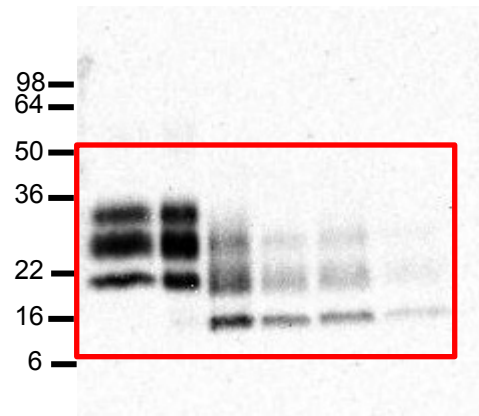

RML

Supplement: Supplementary file 19 [file emmm0007-0339-sd19.pdf]

Figure 2A

Sha31

9A2

12B2

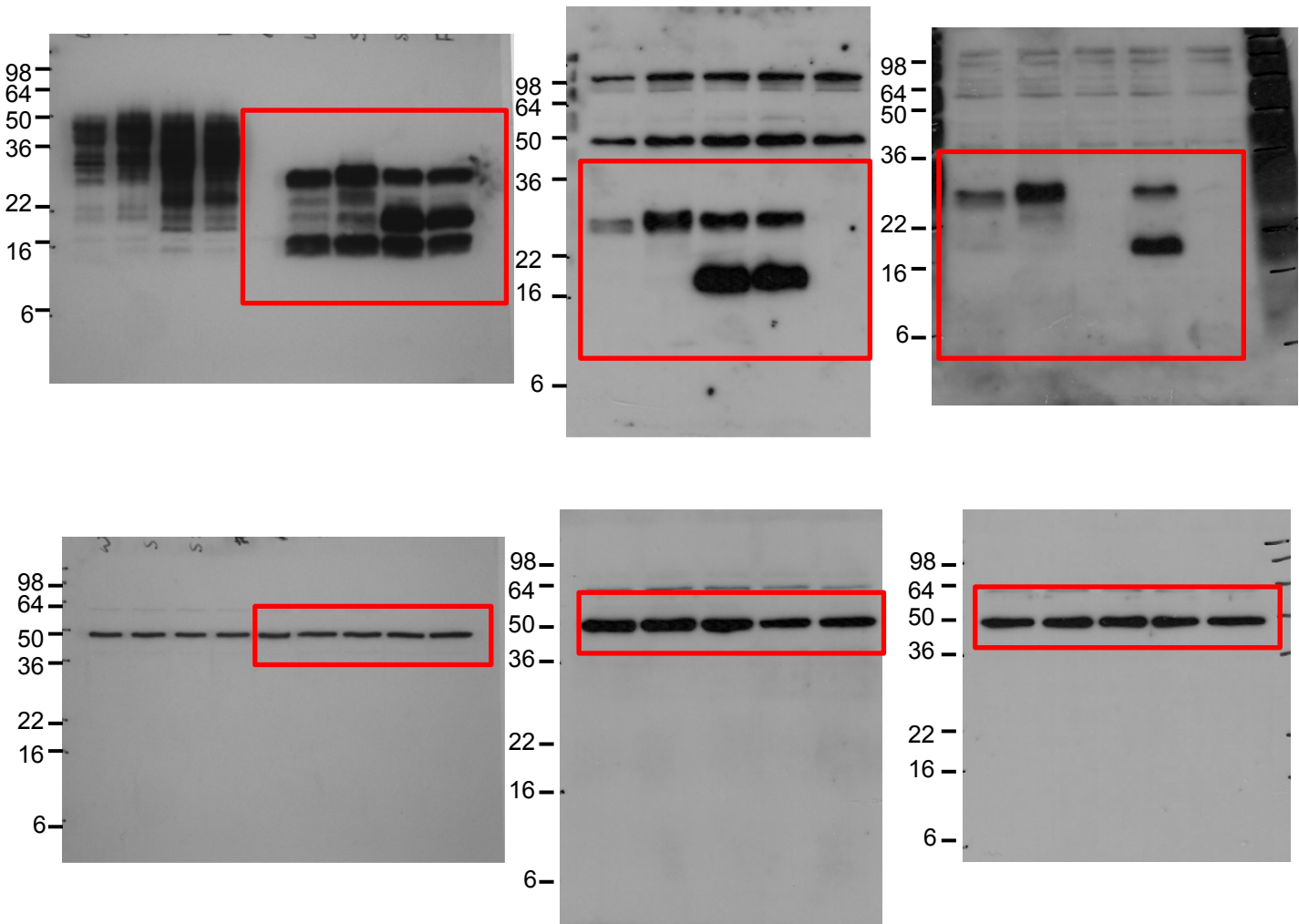

Figure 2B

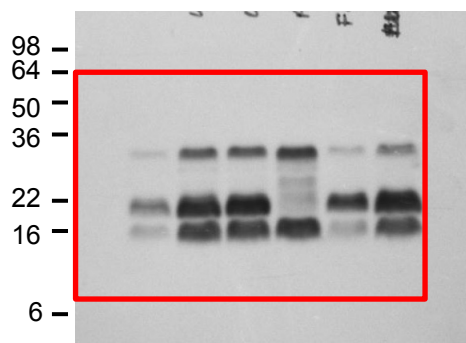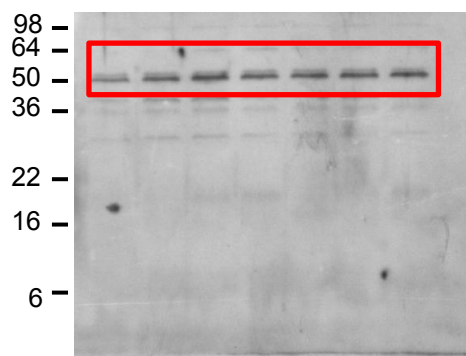

Figure 2C

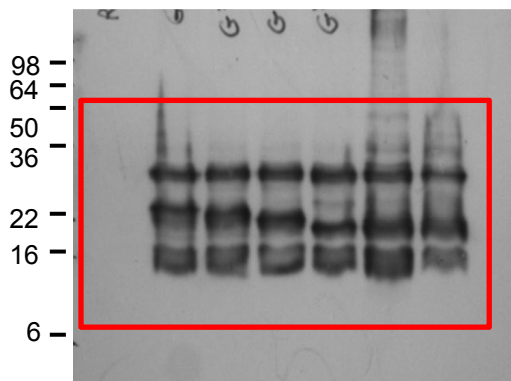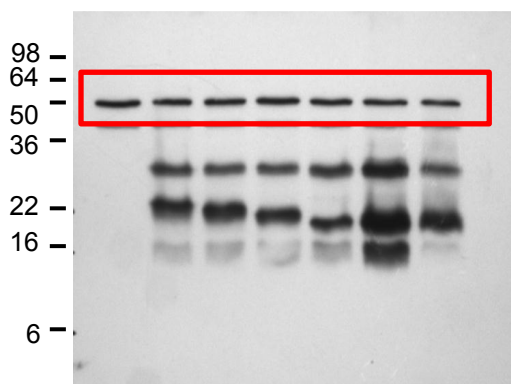

Figure 2D

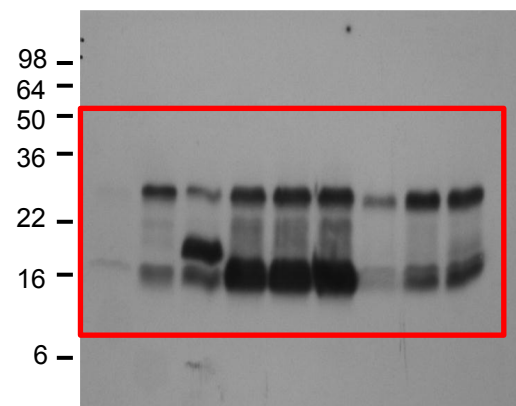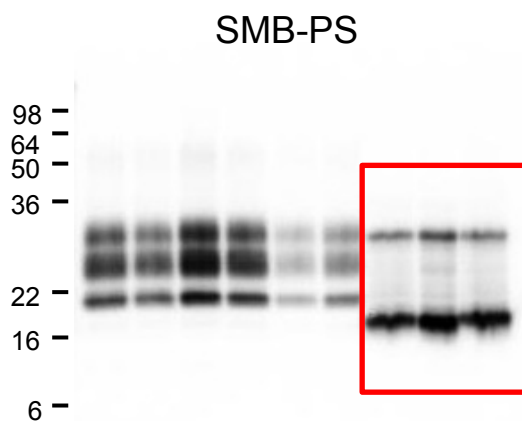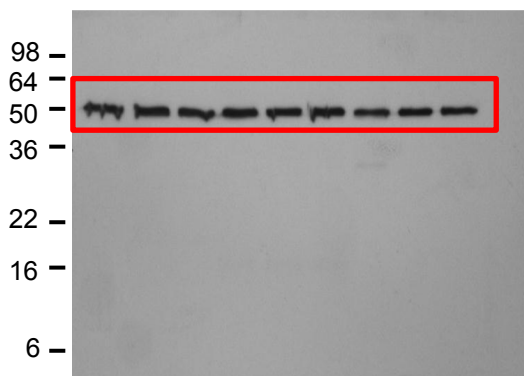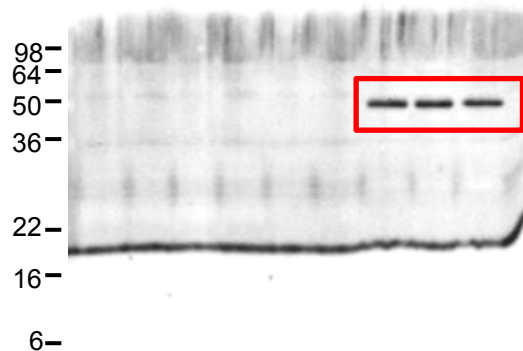

SH-SY5Y

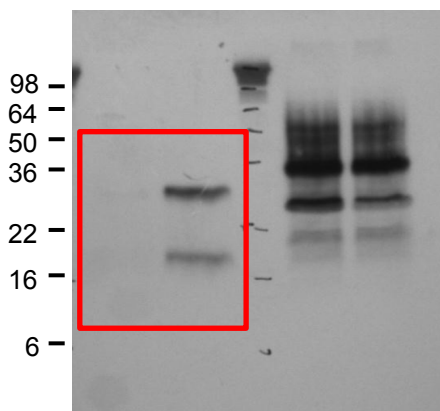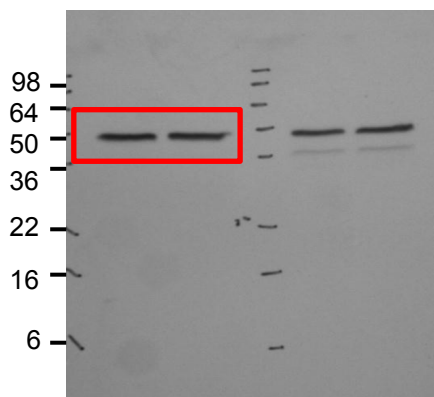

Figure 2E

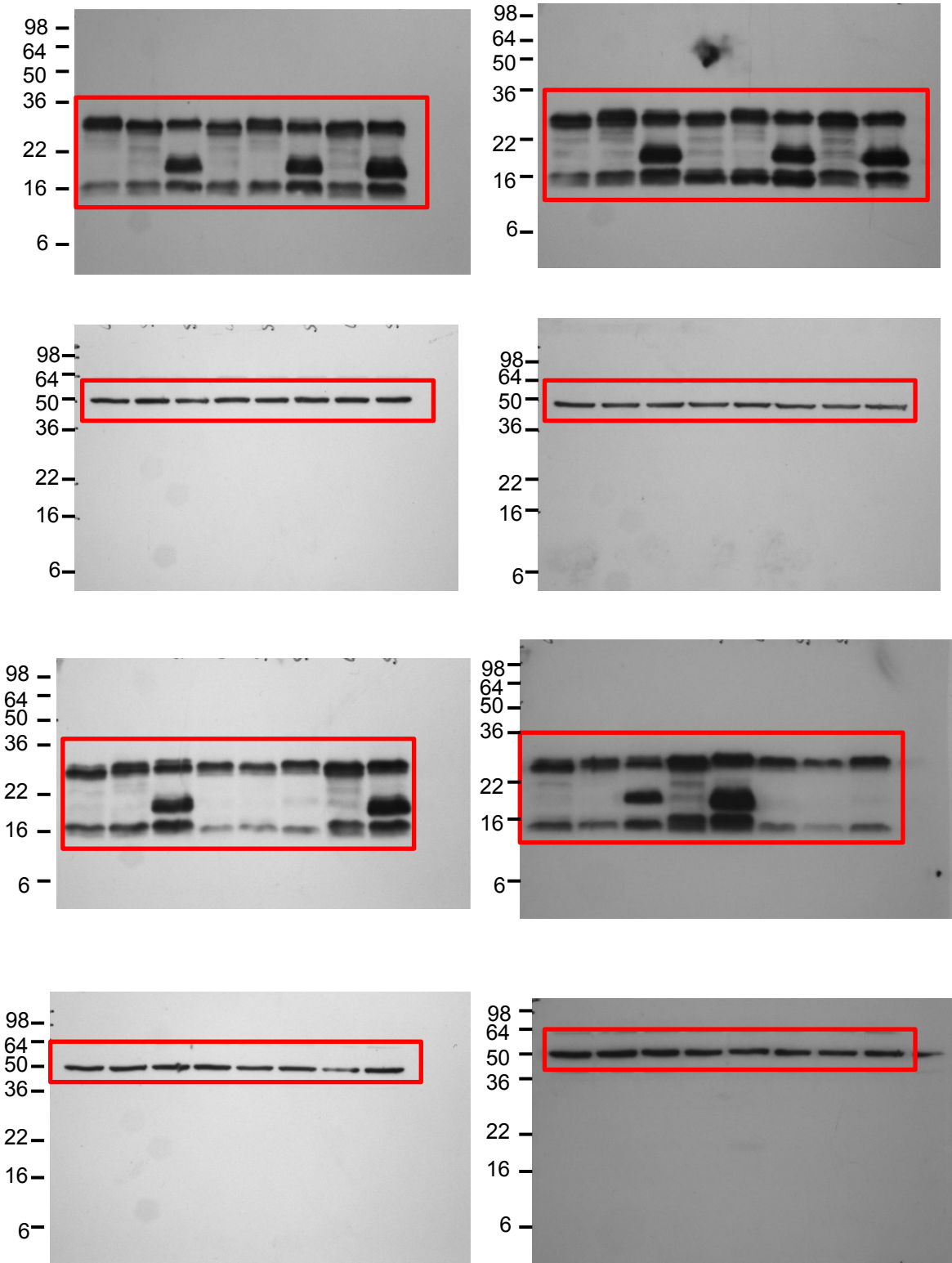

Supplement: Supplementary file 21 [file emmm0007-0339-sd21.pdf]

Figure 3A

Sha31

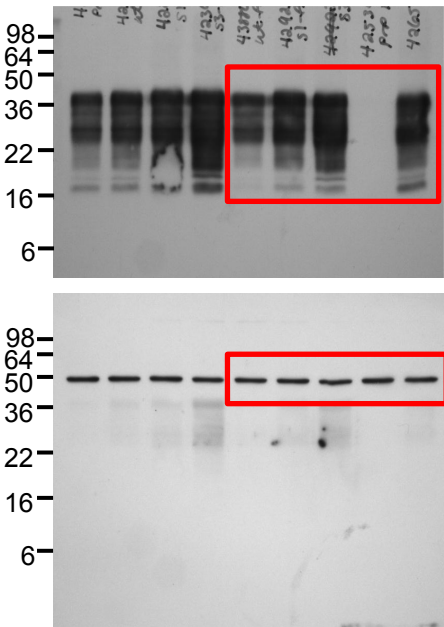

12B2

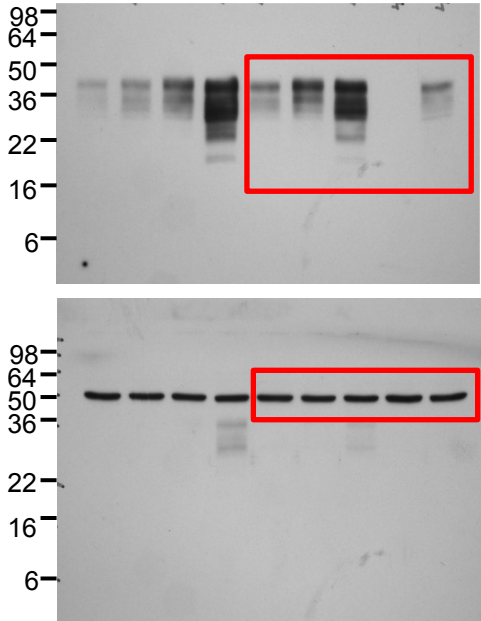

9A2

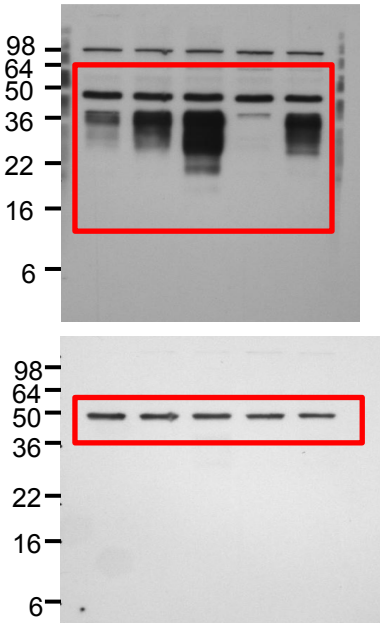

Sha31 PNGaseF

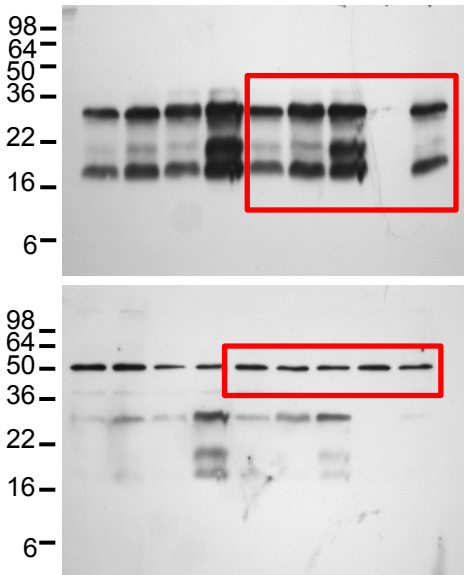

12B2 PNGaseF

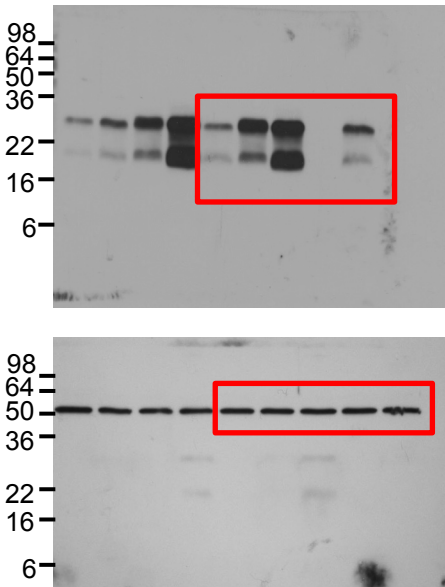

9A2 PNGaseF

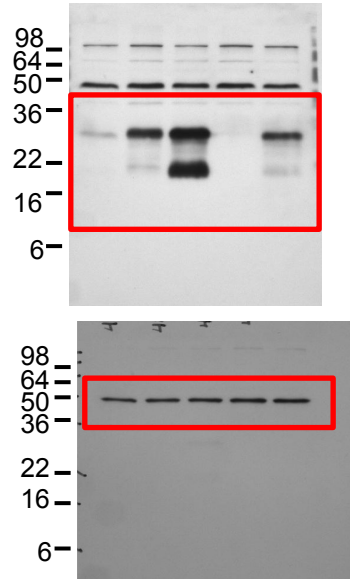

Figure 3C

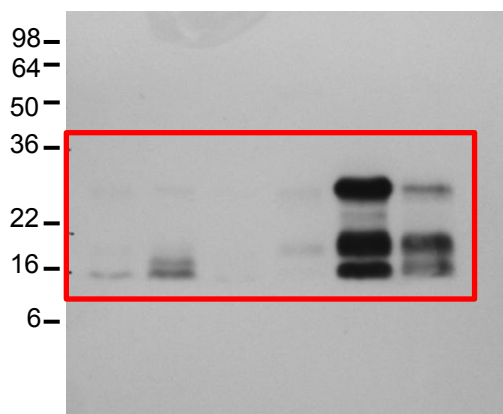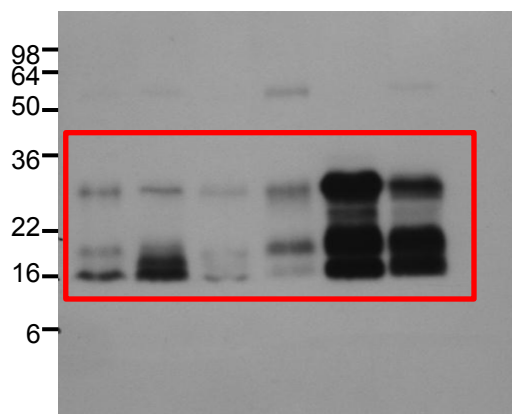

Higher exposure

Supplement: Supplementary file 22 [file emmm0007-0339-sd22.pdf]

Figure 4B

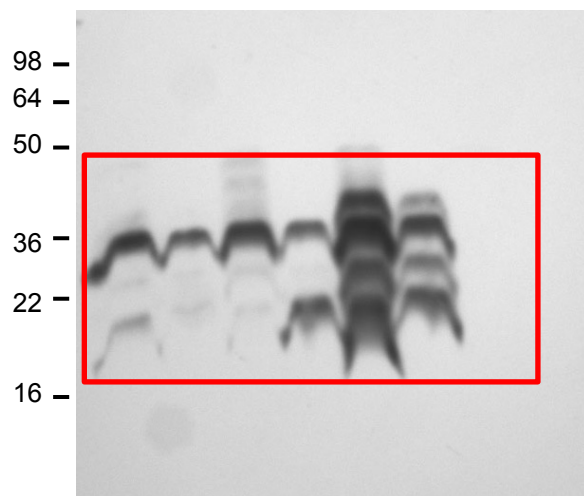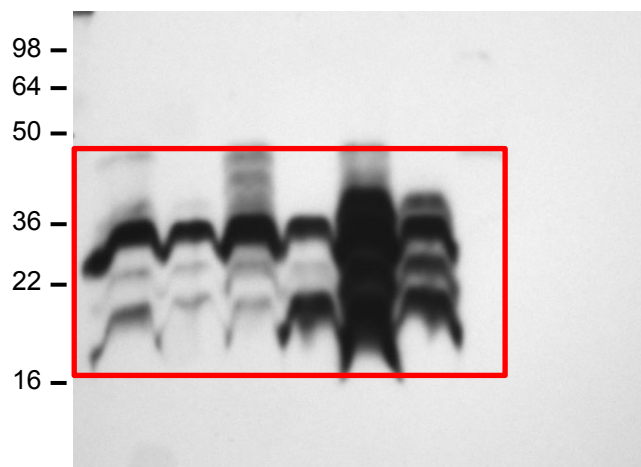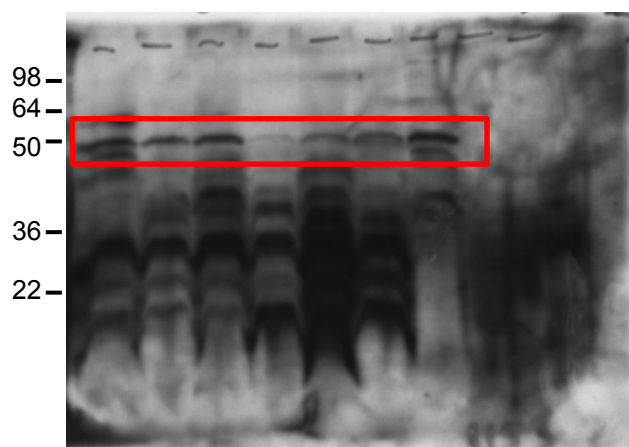

Supplement: Supplementary file 23 [file emmm0007-0339-sd23.pdf]

Figure 5A

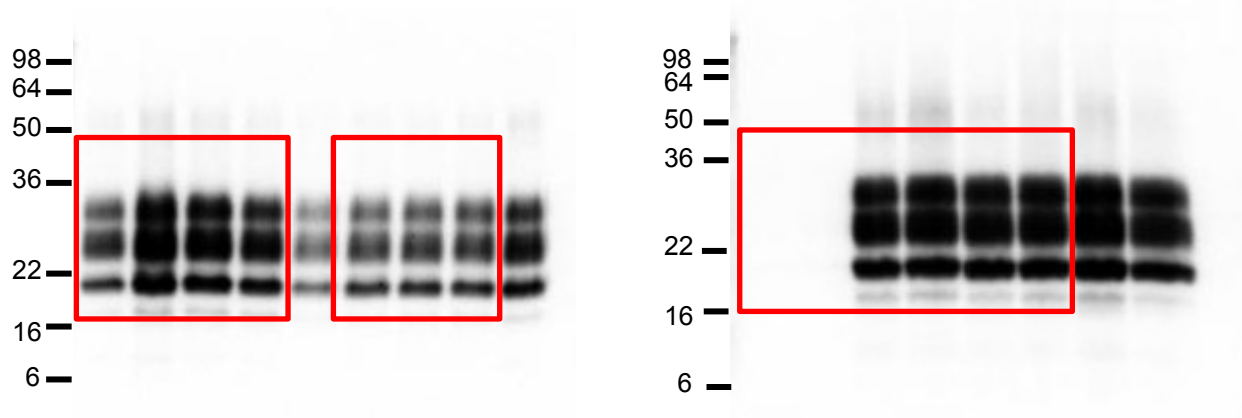

Figure 5B

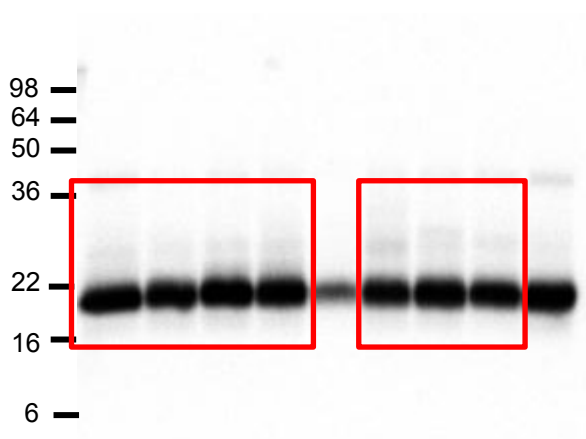

Supplement: Supplementary file 24 [file emmm0007-0339-sd24.pdf]
