# Supplementary material for: Octarepeat region flexibility impacts prion function, endoproteolysis and disease manifestation
Source: EMBO Mol Med. 2015 Feb 6;7(3):339–56. doi: 10.15252/emmm.201404588 (PMC4364950; doi:10.15252/emmm.201404588)
Supplement: Supplementary file 13 [file emmm0007-0339-sd13.pdf]

SUPPLEMENTARY INFORMATION

Table of Contents

	page
Supplementary Materials and Methods	2
Supplementary Figure Legends	4
Table S1	7
Table S2	8

Supplementary Materials and Methods

Peptide Synthesis and Purification

All peptides were prepared using solid-phase Fmoc peptide methodologies on a Liberty 1 peptide synthesizer (CEM Corporation) as described previously (Chattopadhyay et al., 2005). Peptides were cleaved in 95% TFA, 2.5% triisopropylsilane, 1.25% liquidfied phenol, 1.25% water, and then purified by reverse-phase HPLC using a C18 column. Correct peptide masses were confirmed by mass spectroscopy. Materials were then lyophilized, and stored at -20°C. The sequence of the S1 OR peptide is

Ac-KKRPKPWGQPHGGGWPPPHGGSWPPPHGGSWPPPHGGGWGQ-NH2

and for the S3.F88W OR domain peptide the sequence is,

Ac-KKRPKPWGQPHGPGFGQPHGPSFGQPHGPSFGQPHGPGWGQ-NH2

Electron Paramagnetic Resonance (EPR) Spectroscopy

Lyophilized peptide samples were reconstituted in 0.2 µM filtered water, with concentrations quantified by UV-Vis. 300 µL EPR samples for Cu^{2+} titrations of S1 and S3.F88W peptides were made with a final concentration of 100 µM peptide, 25 mM MOPS buffer, pH 7.4, 25% glycerol, and 0-800 µM $Cu(OAc)_2$. EPR spectra were taken with a Bruker EMX X-band spectrometer at a frequency of 9.4 GHz. Spectra were double integrated in LabVIEW (National Instruments) to quantify the concentration of bound Cu^{2+} in each EPR sample.

Metal Addition and Chelation Studies

D-penicillamine (Sigma), tetraethylene pentamine (TEPA; Sigma), tetrathiomolybdate (TTM; Sigma), calcium ethylenediaminetetraacetic acid (CaEDTA; Sigma), $ZnSO_4$ (BDH Chemicals) and $CuSO_4$ (Sigma) were used to treat wt-10 or S3-27 cells near confluence or at 60% confluence in a 12-well plate. The compounds were added to 1mL of OptiMEM (Invitrogen) and incubated with the cells for 24 hours before lysis with RIPA buffer supplemented with Complete Mini Protease Inhibitor (Roche). Lysates were digested with PNGaseF before analysis by western blot.

Metal Analysis by Inductively Coupled Plasma Mass Spectrometry (ICP-MS)

Analysis of OptiMEM (Invitrogen) for copper was provided by the Earth and Atmospheric Sciences Department (University of Alberta) using a Perkin Elmer Elan6000 quadrupole ICP-MS.

Biotin Labeling

Labeling was carried out using components from the Cell Surface Protein Isolation Kit (Pierce). Confluent wt-10, S1-29 and S3-27 cells were washed with cold 1xPBS and labeled with EZ-Link Sulfo-NHS-SS-Biotin (Pierce) for 30mins at 4°C. Cells were washed with cold 1xPBS, DMEM (Invitrogen) supplemented with 10% FBS and penicillin/streptomycin was added and cells were incubated at 37°C for specific time points. After washing with PBS, Trypsin-EDTA (Invitrogen) was added and flasks were rocked at 4°C for 10mins. The cells were removed from the flasks using 1mL of media and centrifuged at 700xg for 5 mins at 4°C. After washing with 1xPBS, the pellet was then resuspended in RIPA buffer supplemented with a Complete Mini Protease Inhibitor (Roche) and lysis occurred on ice for 15mins. The lysates were then centrifuged at 7,000rpm for 3mins at 4°C and the supernatants retained. Equal amounts of total protein from each sample were added to streptavidin beads (Pierce), incubated at room temperature for 2 hours with rotation, and the beads were washed with 1xPBS. The beads were resuspended in RIPA buffer and PNGaseF digestion of the samples occurred as described above. 6X SDS-loading buffer was added and the samples were boiled.

Immunocytochemistry

Cells plated on glass coverslips were rinsed 3x with cold PBS, fixed with 4% paraformaldehyde for 30 min, washed 3x10 min with cold PBS and then permeabilized with 0.2% triton X-100 (Sigma) in PBS. Following washes, the cells were incubated with PrP antibody Sha31 (1:5000 in PBS) overnight at 4 °C. After washing with PBS, cells were blocked with 2% goat serum (Invitrogen) and incubated with Alexa Fluor 594 secondary (Invitrogen, 1:200) for 2 hours at room temperature. Nuclei were stained with 1µg/mL Hoechst in PBS for 10 min. Following 2x 10 min washes with cold PBS, cells were visualized using a Nikon Eclipse 90I motorized upright microscope (Nikon) and a CFI PL 40X/ N.A. 0.75 lens (Nikon) using the following excitation/emission filter properties: 325 – 375/500 – 575 nm with a 495 nm long-pass filter (blue channel) and, 505-615/570-720 nm with a 595 nm long-pass filter (red channel). Images were

acquired with a Retiga 2000R mono cooled camera, fast 1394 using NIS-Elements AR advanced research software at room temperature. Scale bar = 20 μm

Supplementary Figure Legends

Figure S1. EPR spectra of a Cu^{2+} titration of S1 and S3.F88W octarepeat domain peptides. **(A)** X-band EPR spectra of a 100 μM S1 peptide with 0.5 to 8 equivalents of Cu^{2+} (solid lines). As Cu^{2+} is added, Cu^{2+} coordination remains locked in single histidine component 1 (Figure 1B), matching the EPR spectrum of wildtype octarepeat domain peptide loaded with 4 equivalents of Cu^{2+} (dashed line) (Chattopadhyay et al., 2005). Full length MoPrP S1 protein coordinates Cu^{2+} in a manner consistent with S1 peptide. **(B)** X-band EPR spectra of 100 μM S3.F88W peptide with 0.5 to 6 equivalents of Cu^{2+} (solid lines). Cu^{2+} coordination remains locked in multi-histidine component 3 coordination, even as excess Cu^{2+} is added, unlike wildtype octarepeat domain peptide (dashed line, scaled for comparison). As excess Cu^{2+} was added, unbound Cu^{2+} caused signal broadening of the EPR spectra as seen with addition of 6 equivalents of Cu^{2+}, however the integrals of the spectra were not affected. Full length mouse PrP S3.F88W protein was consistent with S3.F88W peptide in Cu^{2+} coordination in the octarepeat domain. **(C)** Plot of equivalents of Cu^{2+} bound vs Cu^{2+} added calculated by taking the double integral of each EPR spectra from the Cu^{2+} titration for both S1 (solid line) and S3.F88W (dashed line) octarepeat domain peptides. S1 peptide is capable of coordinating up to 4 equivalents of Cu^{2+} while S3.F88W, which is locked into component 3 coordination (Figure 1B), is only capable of coordinating a single equivalent of Cu^{2+}.

Figure S2. **Analysis of PrP glycosylation, location and internalization**. **(A)** Western blot on glycosylated PrP shows a similar heterodisperse distribution for WT, S1 and S3 PrP. **(B)** Immunocytochemistry of WT-10, S1-29 and S3-27 in permeabilized RK13 cells analyzed using the PrP antibody Sha31 (red) and Hoechst counterstaining of nuclei (blue). Perinuclear and cell surface PrP staining is visible. Scale bars represents 20 microns. **(C)** Internalization of cell-surface exposed PrPs was analyzed using biotin labeling in WT-10, S1-29 and S3-27 cells with and without trypsin digestion.

Figure S3. Effect of penicillamine treatment on the proteolysis of WT PrP and S3 PrP. **(A)** S3-27 and **(B)** WT-10 cells stably expressing S3 PrP and WT PrP, respectively, were treated

with penicillamine at increasing concentrations and cell lysates were analyzed by western blot using Sha31 after PNGaseF digestion. Cells plated at 95% and 60% confluency were tested.

Figure S4. Effect of CaEDTA, TEPA and TTM on the endoproteolysis of WT PrP and S3 PrP. (A) S3-27 and **(B)** WT-10 cells were treated with chelators CaEDTA, TEPA (tetraethylenepentamine), and TTM (tetrathiomolybdate) at increasing concentrations and cell lysates were analyzed by western blot using Sha31 after PNGaseF digestion.

Figure S5. Effect of copper and zinc supplementation on the endoproteolysis of WT PrP and S3 PrP. Cu or Zn at increasing concentrations was used to treat **(A)** S3-27 and **(B)** WT-10 cells and cell lysates were analyzed by western blot using Sha31 after PNGaseF digestion.

Figure S6. Analysis of PrP expression in transgenic mouse brains using different PrP antibodies. (A) Brain homogenates from transgenic and non-transgenic mice were digested with PNGaseF and analyzed by western blot using the antibodies indicated. **(B)** Densitometric analysis was performed for full-length PrP and is presented as a ratio compared with a WT mouse for each antibody and **(C)** the average is shown.

Figure S7. Rescue of demyelinating polyneuropathy in *Prnp*$^{0/0}$ mice. Sections of sciatic nerve from four aged mice of four distinct genotypes were analyzed using toluidine blue staining. Age ranges were *Prnp*$^{0/0}$, 292-492 days; TgPrP(WT), 492-538 days; TgPrP(S1)-17, 592-720 days, and TgPrP(S1)-19, 604-608 days. No distinctions were noted between males and females and mice of both genders are presented. Scale bar represents 50 μm.

Figure S8. Quantitation of the net morphology of the sciatic nerve. A cross section of a sciatic nerve from a TgPrP(WT) and WT mouse are presented in left and right columns. Top row, unprocessed image. Second row, InCell Translator v2.0.92 Stack Builder was used to convert images to GE InCell.xdce format, then an analysis protocol was implemented with the InCell Developer Toolbox v1.9 to delineate individual fibres in the target through object segmentation (kernel size of 5 and using sensitivity in the range 65-85, depending on image gray level and contrast). Third row, post-processing was performed to fill in hollow fibres and hence the net area for all fibres. Fourth row, assignment of nerve perimeter to derive the total nerve cross-sectional area. Data from row 3 is divided by row 4 data to derive the % of the nerve occupied by fibres (see Figure 4C)

Figure S9. GdnHCl analysis of infected transgenic mouse brain homogenates. PrP^{Sc} from brain homogenates of infected TgPrP(S1) and TgPrP(S3) were treated with increasing concentrations of GdnHCl, PK-digested, and analyzed by western blot using the PrP antibody Sha31. Compared with the RML brain homogenate control, the pattern of PK-resistance in infected TgPrP(S1) and TgPrP(S3) were similar.

Figure S10. Comparison of D/N ratio for PrP^C in uninfected mice. The conformation-dependent immunoassay revealed no significant differences in signals before and after guanidine denaturation (denature: native, "D/N" ratio) in uninfected mice using the 12B2 antibody.

Figure S11. **Analysis of PrP species by velocity gradient centrifugation and CDI**. The amount of PrP^C, total PrP^{Sc} and PK-resistant PrP^{Sc} ($rPrP^{Sc}$) are shown for each fraction after velocity gradient centrifugation in mice expressing **(A)** WT PrP, **(B)** S1 PrP and **(C)** S3 PrP. Note that the scale of the y-axis changes between the columns and rows to accommodate different levels of accumulation of PrP species.

Figure S12. Comparison of D/N ratio for PrP^{Sc} in infected mice. Denatured/native ("D/N") CDI signals were determined for brain homogenates from mice expressing WT PrP, S1 PrP and S3 PrP that were treated and not treated with 4M GdnHCl at 80ºC before and after PK digestion. PrP^{Sc} from mice expressing WT PrP and S1 PrP had increased D/N values after PK treatment, whereas PrP^{Sc} from mice expressing S3 PrP had decreased values.

Reference:

Chattopadhyay, M., Walter, E.D., Newell, D.J., Jackson, P.J., Aronoff-Spencer, E., Peisach, J., Gerfen, G.J., Bennett, B., Antholine, W.E., and Millhauser, G.L. (2005). The octarepeat domain of the prion protein binds Cu(II) with three distinct coordination modes at pH 7.4. J Am Chem Soc *127*, 12647-12656.

Table S1. Failure to detect abnormal PrP species in uninfected TgPrP(S1) and TgPrP(S3.F88W) mice

Analysis	Mouse	Age (days)	# of animals
Western blot[a]	TgPrP(S1)-17	566-669	0/4
	TgPrP(S3.F88W)-14	325-367	0/2
	TgPrP(S3.F88W)-35	427-621	0/2
	TgPrP(WT)	484	0/1
IHC[b]	TgPrP(S1)-17	308-690	0/8
	TgPrP(S3.F88W)-14	325-584	0/9
	TgPrP(S3.F88W)-35	336-675	0/11
	TgPrP(WT)	447-671	0/5

[a]Digestions were performed at a concentration of 50ug/mL PK

[b]IHC was performed using SAF83 antibody after treatment using 4 M guanidine thiocyanate

Table S2. Failure to obviate transmission barriers by expression of S1 and S3 mouse PrP alleles in transgenic mice

Mouse line	Inoculum	# euthanized by 400 dpi
TgPrP(S3.F88W)-35	Classical sheep scrapie	0/4
	Classical BSE	0/5
TgPrP(S1)-17	Classical sheep scrapie	0/11
	Classical BSE	0/9
TgPrP(WT)	Classical sheep scrapie	0/11
	Classical BSE	0/14
