# Supplementary material for: Octarepeat region flexibility impacts prion function, endoproteolysis and disease manifestation
Source: EMBO Mol Med. 2015 Feb 6;7(3):339–56. doi: 10.15252/emmm.201404588 (PMC4364950; doi:10.15252/emmm.201404588)
Supplement: Supplementary file 20 [file emmm0007-0339-sd20.pdf]

EMBO Molecular Medicine

Octarepeat region flexibility impacts prion function, endoproteolysis and disease manifestation

Agnes Lau, Alex McDonald, Nathalie Daude, Charles E. Mays, Eric D. Walter, Robin Aglietti, Robert C. C. Mercer, Serene Wohlgemuth, Jacques van der Merwe, Jing Yang, Hristina Gapeshina, Chae Kim, Jennifer Grams, Beipei Shi, Holger Wille, Aru Balachandran, Gerold Schmitt-Ulms, Jiri G. Safar, Glenn L. Millhauser and David Westaway

Corresponding author: David Westaway, University of Alberta

Review timeline:

Submission date:	29 August 2014
Editorial Decision:	26 September 2014
Revision received:	02 December 2014
Editorial Decision:	18 December 2014
Revision received:	31 December 2014
Accepted:	08 January 2015

Transaction Report:

(Note: With the exception of the correction of typographical or spelling errors that could be a source of ambiguity, letters and reports are not edited. The original formatting of letters and referee reports may not be reflected in this compilation.)

Editor: Céline Carret

1st Editorial Decision 26 September 2014

Thank you for the submission of your manuscript to EMBO Molecular Medicine. We have now heard back from the three referees whom we asked to evaluate your manuscript.

As you will see, all three referees are enthusiastic about the study but do have suggestions and recommendations to further improve conclusiveness and clarity especially regarding the potential clinical implications of your findings and general interest, both points being particularly important for our scope.

I will not get into experimental details, but we feel that the referees' reports are very clear and nicely detailed and we would strongly encourage you to address all issues raised as recommended.

Given these evaluations, I would like to give you the opportunity to revise your manuscript, with the understanding that the referees' concerns must be fully addressed and that acceptance of the manuscript would entail a second round of review. Please note that it is EMBO Molecular Medicine policy to allow only a single round of revision and that, as acceptance or rejection of the manuscript will depend on another round of review, your responses should be as complete as possible.

EMBO Molecular Medicine has a "scooping protection" policy, whereby similar findings that are published by others during review or revision are not a criterion for rejection. Should you decide to submit a revised version, I do ask that you get in touch after three months if you have not completed it, to update us on the status.

Please also contact us as soon as possible if similar work is published elsewhere. If other work is published we may not be able to extend the revision period beyond three months.

I look forward to seeing a revised form of your manuscript as soon as possible.

***** Reviewer's comments *****

Referee #1 (Remarks):

The manuscript of Lau et al. investigates the role of the octarepeat region of the prion protein, situated in the unstructured N-terminal domain, by rationally designing two new PrP alleles. The idea is to study, individually, two distinct conformations of PrP, restricting inter-conversion between them. Authors first identify the residue substitutions in the octarepeat region, and then they generate two transgenic model systems to study the effects of different mutations. This systematic study addresses scientifically relevant questions. There are, however, still points requiring further clarification/discussion.

Major concerns:
1) The two new alleles are not naturally occurring. Hence, although the study may give important information on the molecular mechanism of prion pathology and how the octarepeat region impacts prion function, the real clinical relevance in the human disease is not clear.
2) Fig 2 shows the protein expression analysis by western blot of the effects of the different mutations on the cleavage of C1 and C2 fragments. The question whether some aminoacid substitutions may induce intracellular aggregation of PrP is never addressed. Only the soluble pool of proteins is analyzed. The analysis of the insoluble pellets should be shown not only because is particularly relevant to the possible pathogenic activity of the abnormally produced fragments, but also because it can explain differences in expression level (see for example lower expression of triple or quadruple mutations (F2F3F4 or F1F2F3F4).
3) Fig 3 shows the protein expression analysis in brain homogenates of transgenic mice. Again, the high variability of protein expression level that Authors find in their gels is not discussed and it is never related to the possibility of intracellular aggregation or higher degradation of PrP.
4) Fig 3B shows the ratio of C2 to full-length PrP in TgPrP(S3)-35. These mice have a 3-fold increase of C2 fragment versus WT, but also total PrP (FL + C2) is markedly increased. Since PrP expression level influences prion replication and pathogenesis, this point should be better discussed.

Referee #2 (Remarks):

The manuscript by Agnes Lau and colleagues describes that flexibility in the octarepeat region of the prion protein impacts endoproteolysis and prion disease manifestation. In an extremely comprehensive and thorough approach, the authors generated two artificial 'alleles' which were locked in their N-terminal structure and compared them to wild-type PrPc which has an unstructured N-terminal region. When expressed in cultured cells, the authors observed a substantial increase in PrP beta-cleavage resulting in more C2 fragment in RK13 cells for the S3 allele. This was not the case in various other cell lines tested. The authors then generated transgenic mouse lines and mice expressing an S3 allele again showed pronounced C2 fragments. When these mice were infected with RML prions, the authors found an uncoupling of prion titres and incubation times, most pronounced for the S3 PrP. Having lower prion titers but shorter incubation times implies a signaling function for the C2 fragment in this context. Overall, this is a very interesting study and the authors produced important new data.
An alternative explanation for the observed cell type and tissue differences in C2 fragment levels might be variations in the subcellular trafficking of PrPs which either target or bypass a subcellular compartment in which this proteolytic activity resides. The authors might want to comment on such a possibility. A polarized sorting of PrP was described for epithelial cells (Sarnataro et al., 2002; Pasquet et al., 2004) and the authors could characterize whether their PrP constructs all behave

identical in a given cell type. Figure E2 provides some surface biotinylation data but it is not clear whether also the C2 fragment undergoes surface biotinylation and whether there are cell type and/or PrP construct differences. Since the authors postulate a protease which is involved in the production of C2 PrP, application of protease inhibitors in RK13 cells might be interesting and reveal the nature of this protease. Finally, to address the molecular basis for the dissociation of prion titers and incubation time, the authors might want to analyze the underlying cell death mechanisms in more detail, although this probably is beyond the scope of this manuscript. Apart from these mainly minor caveats, this is a technically very well done manuscript. This work is of excellent quality and is likely to have a great impact for future studies.

Minor points:
1.) Providing the ratios of wt, C1 and C2 fragments for the in vitro data might be helpful, as done for the in vivo data in Fig. 3B.
2.) No blots without PNGaseF treatment are shown for the in vitro data. Are all PrPs indicative in their appearance of proper glycosilation?
3.) On page 8, second paragraph, an autocatalytic event is mentioned. This statement stands quite alone and might need some more explanation.

Referee #3 (Remarks):

The prion hypothesis was debated for many years but is generally accepted today and can be regarded as proven. The prion protein PrP in its pathogenic conformation PrSc is the infectious agent of prion diseases. That does not infer that the molecular mechanisms of infectivity and pathogenicity are known today in great detail. Since it is known that some principles of prions have general importance for protein misfolding diseases like Alzheimer, Parkinson etc. the importance of prion mechanism is much more general than anticipated for the so-called rare disease, i.e. the human prion disease Creutzfeldt-Jakob-disease. In the present manuscript Westaway et al. deal with a particular structure-function problem of PrP. PrP consists of a more or less globular C-terminal domain (aa90-aa231) and a flexible N-terminal domain. Whereas the (90-231)-domain is known since long as essential for infectivity and pathogenicity the function of the flexible N-terminus is not well known, but is exactly the subject of the present manuscript. In vivo cleavage leads to the fragments C1 and C2 in the linker region between the flexible and the globular region; the function of these fragments is not well understood. Five octarepeats (8 aa) and two hexarepeats (6 aa) in the N-terminus form binding sites for three or four copper-ions. The authors have constructed two groups of mutations, which either bind only one copper-ion (S3) with a compact structure or bind similarly to the wild type 3-4 copper-ions (S1) with a flexible structure.

Mechanisms of infectivity and pathogenicity of prions are of general interest in medicine, the detailed functional problems of the N-terminus are of interest mainly for prion-researchers. Therefore, the whole work is at the edge between general and special interest.

The authors apply a whole battery of methods: transgenetics, biochmical and biophysical analysis, cell biology test, in vivo tests etc.. The experimental effort is impressive and of high quality. The functional problem is not novel but the very detailed experimental approach is novel, and the complexity of the problem has never been dealt with before. Parameters like C1- and C2-expression, stability against GdnHCl denaturation, resistance/sensitivity for PK-proteolysis, spot counts in cell assays, incubation times and infectivity titers have been analysed. As far as possible the analysis was applied to the cellular form of PrP isolated from brain homogenate and to the pathogenic form PrPSc. Corresponding to the many methods also many results are presented. They are well presented in the figures, not always easy to follow by non-specialists.

The results show quantitative differences, i.e. not yes-or-no-answers. Accordingly the discussion has to be somewhat vage and the conclusions show tendencies only. The referee can see two lines of conclusions: (i) Not the bound copper-ions are essential for the function of the N-terminus but the

flexibility. This conclusion is more derived from the kind of aa-substitutions than from experimental facts: (ii) The N-terminus regulates more the pathology of the disease than the infectivity. It would be in line with the assumption that pathology and infectivity of prion diseases are uncoupled.
The somewhat weak conclusions appear inherent in the problem. The referee cannot recommend a clear cut experiment to improve the strenght of the conclusions. Also the function of the C1- and C2-fragments could not be clarified; for the sake of completeness the features of the proteolytic fragment PrP27-30 could be included into the discussion.

In summary, a very extended, rigorous and detailed experimental work on a complex question. It is of relevance for our basic understanding of prion diseases on the level of molecular medicine. At present consequences for treatment or theapy of the disease cannot be drawn.
The high quality of the work deserves publication, the same is true in respect to the interest of the reviewer; the editor has to decide about the general interest of the journal.

1st Revision - authors' response 02 December 2014

We thank the referees for their insightful remarks and supportive comments. We also thank the editor for the guiding comments and the punctual manner in which the review process was executed. The point-by-point response is detailed below and new text is highlighted in yellow in a modified manuscript.

Referee #1 (Remarks):

The manuscript of Lau et al. investigates the role of the octarepeat region of the prion protein, situated in the unstructured N-terminal domain, by rationally designing two new PrP alleles. The idea is to study, individually, two distinct conformations of PrP, restricting inter-conversion between them. Authors first identify the residue substitutions in the octarepeat region, and then they generate two transgenic model systems to study the effects of different mutations. This systematic study addresses scientifically relevant questions. There are, however, still points requiring further clarification/discussion.

Major concerns:
1) The two new alleles are not naturally occurring. Hence, although the study may give important information on the molecular mechanism of prion pathology and how the octarepeat region impacts prion function, the real clinical relevance in the human disease is not clear.

The remit of EMBO Molecular Medicine concerns "a new research discipline at the interface of clinical research and basic biology" and we believe our paper including analyses of animals with clinical disease does fall into this broad territory.

With regards to genetic prion diseases, mutations in familial prion disease do not represent a uniform sampling of all PrP coding nucleotides but are instead skewed by sequence-specific mutagenic mechanisms. For the octarepeat region (OR), naturally occurring mutant human alleles affect the number of tandem octarepeat units and likely arise through unequal crossing over between strands or slippage of DNA replication forks on a DNA strand. As the full mutational repertoire of the PrP OR is not represented by the familial mutation database, we used site-directed mutagenesis to address a hypothesis as to how conformational flexibility and ligand binding might affect this region's role in maintaining peripheral myelination and susceptibility to infection.

In our approach, the iterative design with synthetic peptides to derive the S1 and S3 allelic versions of PrP (Figure E1 and supplementary materials) was performed with a mouse version of the OR. Unlike human PrP, the mouse PrP OR has serine residues in two of the central octarepeats. To reduce the number of variables in the experiment (e.g., the possibility of N-terminal/C-terminal

interaction in cis; Spevacek et al, Structure, 2013; Sonati et al, Nature, 2013) the S1 and S3 OR variants were engineered into a wt mouse PrP backbone to match the genetic background of the host and its endogenous macromolecules. If we had conducted the prion challenges with human prions, we would also have had to introduce a C-terminal PrP domain akin to human PrP to avoid a species-barrier to infection. So while the study does not deal directly with CJD and GSS pathogenesis, we have uncovered a new effect in the biology of the OR and set the stage for future studies on human prion disease.

2) Fig 2 shows the protein expression analysis by western blot of the effects of the different mutations on the cleavage of C1 and C2 fragments. The question whether some amino acid substitutions may induce intracellular aggregation of PrP is never addressed. Only the soluble pool of proteins is analyzed. The analysis of the insoluble pellets should be shown not only because is particularly relevant to the possible pathogenic activity of the abnormally produced fragments, but also because it can explain differences in expression level (see for example lower expression of triple or quadruple mutations (F2F3F4 or F1F2F3F4).

3) Fig 3 shows the protein expression analysis in brain homogenates of transgenic mice. Again, the high variability of protein expression level that Authors find in their gels is not discussed and it is never related to the possibility of intracellular aggregation or higher degradation of PrP.

*Dealing first with aggregation, this question is relevant when dealing with mutant PrPs and we reasoned that long-term observation of the Tg mice might comprise the most sensitive indicator of protein aggregates, be they intracellular or extracellular. At one stage we were particularly interested in aged TgPrP(S3.F88W) mice because unpublished tissue culture studies showed an interaction of the S3 allele with certain type of artificial cell medium (presented as a poster at the international Prion congress in Trieste in May 2014). To assess this question in vivo we observed our Tg animals for extended periods of time. Our analyses listed in the first submission included observation of aged animals for neurological disease (Table E1) and assessment of brain homogenates for proteinase K resistant PrP (Table E1); both types of analysis were negative. We may have erred in not mentioning the assessment of protease-resistant PrP by gel analysis (negative, included in Table E1) in the main text of the original version and a minor addition adjustment to our prose has been incorporated into the revised submission. This reads… "we were unable to detect spontaneous neurologic disease in TgPrP(S1) and TgPrP(S3.F88W) mice at nearly two years of age, nor the presence of a PK-resistant PrP species by western blot of brain homogenates from these mice (**Table E1**)." The new text is on page 8, third paragraph.*

Also, we have yet to find anything remarkable by immunohistochemical analysis of the aged TgPrP(S1) and TgPrP(S3.F88W) animals. While defining a spontaneous disease would have been exciting in some respects, we note that excluding this type of process was a useful preamble for interpreting the results of prion inoculation experiments.

The gels of S1 and S3-expressing cells represent denaturing SDS-PAGE analyses of total cell lysates made in RIPA buffer (i.e., a detergent lysis including the intracellular pool of proteins) without an ultracentrifuge spin to generate a pellet and supernatant. There is, however, a 7,000xg spin to pellet cellular debris with the supernatant then being boiled and run on SDS-PAGE.

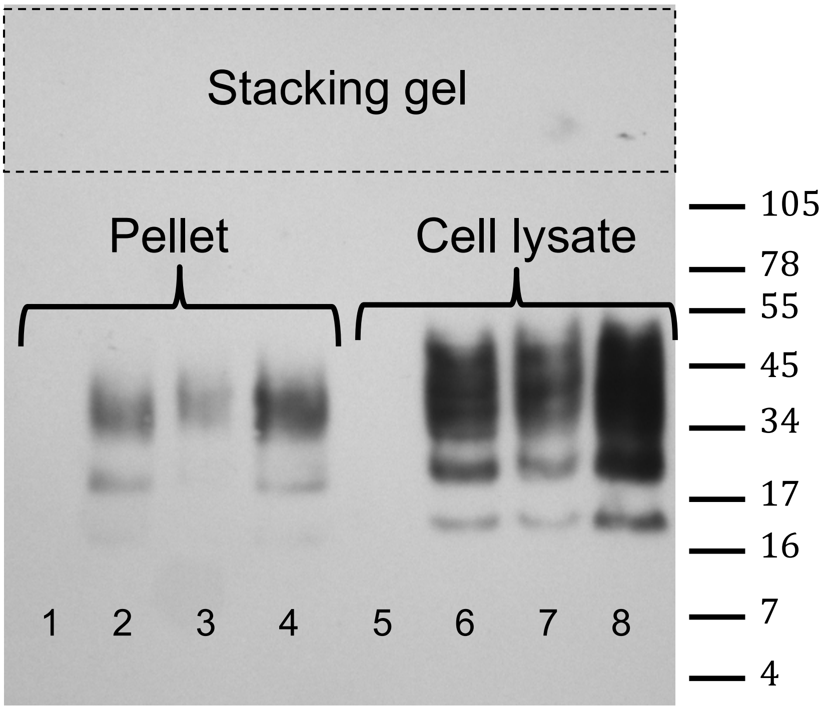

To address the referee's point we have added two new types of analysis. First (above) we have looked at the stacking gel of our gels from transfected cells grown in DMEM to see if any proteins (immunoblot signal) have been left behind in the stack. This is not the case, as there is no signal in this area of the blot (dotted lines). The lanes in the gel above correspond to: 1 and 5 untransfected RK13 cells; Lanes 2 and 6 wt PrP, lanes 3 and 7 S1PrP, lanes 4 and 8 S3 PrP. Also, most of the PrP signal is in the cell lysate (lanes 5-8) not the low speed pellet (lanes 1-4), and there is no overt underrepresentation of the S3 PrP protein compared to the other alleles (lanes 4 and 8). The ratios of pellet:lysate signal are not obviously different between the three alleles.

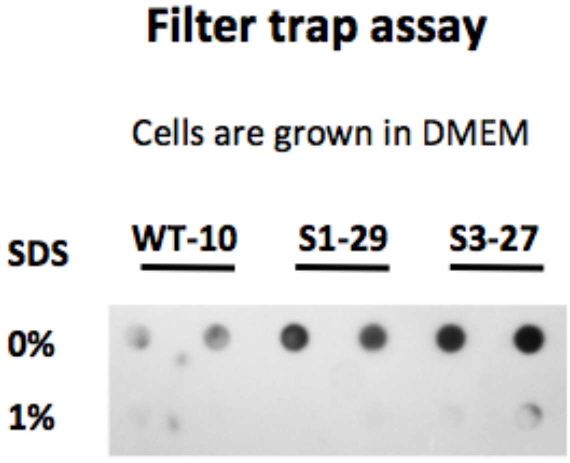

Secondly, (above) we have now added a further analysis to look for PrP aggregates in the cells, a filter-trap assay. This technique does not include a centrifugation step of any kind yet it also failed to reveal data suggestive of aggregation in S1 and S3 PrP-expressing cells. We have previously used this assay successfully for a non-infectious version of PrP where aggregates persist in the presence of 1% or higher SDS. However in the lysates of RK13 cells expressing S1 and S3 PrP, little to no signal is seen in the samples treated with 1% SDS.

In sum we do not believe that failure to detect higher order aggregates is a substantial source of error in our analyses.

We will next deal with the issue of variation within the gel immunoblot signals that that might concern the level of protein expression.

There are two parameters in Fig. 3 that are intrinsic sources of variation. The first is our use of a panel of antibodies, which have minor differences in affinity even with the same allele (Zanusso et al., PNAS 95: 8812-8816, 1998; Liu et al., Brain Research 896: 118-129, 2001). The second is that the alleles are each represented by different Tg lines. By nature of the pronuclear microinjection method, each Tg line contains a different transgene array integrated at a distinct chromosomal insertion site. The different Tg lines engender different levels of steady-state PrP^C *expression.*

Distinctions between transgenic lines were noted not just by western blot analyses (Fig. 3), but also by conformation dependent immunoassay (CDI). Data from the two types of analyses were presented in Table 1 and agree rather well. From a technical standpoint, CDI is performed on homogenates in a plate format, so the consideration that hypothetical aggregates formed by S1 or S3 PrP (see also comments above) might be "left behind" in a pellet or in the sample well of a protein stacking gel does not apply.

With the above points about antibody panels and distinct Tg lines noted, we now consider the comment about variation between protein gels. Experiments presented in the paper do derive from the efforts of different lab members and in this respect will encompass variations originating from minor differences in technique. Selective presentation of experiments could have been used to obtain neater clustering of data, but this would then impose a type of artificiality. Overall we are convinced we have presented a realistic maximum "spread" of the data yet can reach firm conclusions about relative expression.

For the comment about "higher degradation" of PrP, if this is taken to mean increased endoproteolysis as a result of expression level, this can be absolutely excluded; Tga20 mice with about 6x PrP overexpression do not overproduce C2 fragment. We have added a sentence to clarify this point. "Though Tg mice expressing S3 PrP have increased levels of full length PrP compared to WT, this alone does not account for the increased levels of C2 fragment as tga20 mice overexpressing PrP 6-fold do not overproduce the C2 fragment (Fischer et al., 1996, Westergard et al., 2011)". The new text is on page 13, first paragraph.

If by "higher degradation", the referee means net protein destruction (so as to reduce the steady state levels of mutant alleles) this might be an issue if the Tg lines expressed less than wt. For various reasons presented here (closely concordant results from blot and CDI analyses) this was not the case and expression from the mutant alleles equals or transcends wt expression levels.

4) Fig 3B shows the ratio of C2 to full-length PrP in TgPrP(S3)-35. These mice have a 3-fold increase of C2 fragment versus WT, but also total PrP (FL + C2) is markedly increased. Since PrP expression level influences prion replication and pathogenesis, this point should be better discussed.

We have reconstructed a whole paragraph in the Discussion to deal with this issue: "In the case of S3 PrP, the Tg mice expressing this allele succumbed to prion disease ~60 days earlier than TgPrP(S1)-17 and TgPrP(S1)-19 mice. Two variables are relevant in drawing these comparisons: the levels of full-length PrP^C *in uninfected TgPrP(S3.F88W)-14 and TgPrP(S3.F88W)-35 mice are increased 0.7x over TgPrP(S1)-17 and TgPrP(S1)-19 mice, while the ratio of C2 production changes by greater than 3-fold (**Table 1**). Regarding the former, it is well known that increasing full-length* PrP^C *above WT levels decreases disease duration (Carlson et al., 1994). In the case of mice expressing WT PrP at 3-4x levels incubation times with the same RML prion isolate were 100*

*± 17 days (Fischer et al., 1996) versus 89.1 ± 2.5 and 84.1 ± 2.6 in the TgPrP(S3.F88W)-14 and TgPrP(S3.F88W)-35 lines with 3x expression, respectively (**Tables 1, 2**); these figures are in reasonable agreement given the margins of error in scoring the terminal phase of prion disease and could suggest that expression level is the key variable in arriving at incubation times less than 100 days. However, quite curiously, S3 PrP alleles also have levels of pathogenesis-associated species that are below those of WT controls; lower spot counts in SSCA (**Fig. 7A**), a 4-fold drop in $rPrP^{Sc}$ and a >6 fold drop in protease-sensitive PrP^{Sc} isoforms at disease endpoint (**Table 3, Fig. E11**). Our studies also found a prolongation of incubation time period upon passage through TgPrP(S3.F88W)-35 mice (P<0.0001) but the drop in infectious titre that did not reach significance (noting that a one log-unit drop defines a minimum for a significant change in titre determined by bioassay (Prusiner, 1987)). To resolve the paradox of lower amounts of pathogenic species yet abbreviated incubation times we infer that C2 PrP – normally present at much lower levels than C1 PrP – may have a greater ability to perform signaling from pathogenic forms of PrP (**Fig. 7C**). Future experiments with knock-in mice and ex vivo cultures may be of great use to tease apart the relationships between truncated PrP^C species and toxic signaling originating from PrP^{Sc} or Ab oligomers." The new text starts on page 15, last paragraph.*

Referee #2 (Remarks):

The manuscript by Agnes Lau and colleagues describes that flexibility in the octarepeat region of the prion protein impacts endoproteolysis and prion disease manifestation. In an extremely comprehensive and thorough approach, the authors generated two artificial 'alleles' which were locked in their N-terminal structure and compared them to wild-type PrPc which has an unstructured N-terminal region. When expressed in cultured cells, the authors observed a substantial increase in PrP beta-cleavage resulting in more C2 fragment in RK13 cells for the S3 allele. This was not the case in various other cell lines tested. The authors then generated transgenic mouse lines and mice expressing an S3 allele again showed pronounced C2 fragments. When these mice were infected with RML prions, the authors found an uncoupling of prion titres and incubation times, most pronounced for the S3 PrP. Having lower prion titers but shorter incubation times implies a signaling function for the C2 fragment in this context. Overall, this is a very interesting study and the authors produced important new data.

An alternative explanation for the observed cell type and tissue differences in C2 fragment levels might be variations in the subcellular trafficking of PrPs which either target or bypass a subcellular compartment in which this proteolytic activity resides. The authors might want to comment on such a possibility. A polarized sorting of PrP was described for epithelial cells (Sarnataro et al., 2002; Pasquet et al., 2004) and the authors could characterize whether their PrP constructs all behave identical in a given cell type.

Figure E2 provides some surface biotinylation data but it is not clear whether also the C2 fragment undergoes surface biotinylation and whether there are cell type and/or PrP construct differences.

We appreciate these supportive comments and we will address these related issues together.

We do not have the kidney and thyroid cell-lines growing in the lab and as we identified four cell lines in Fig 2D that do not have the C2 activity versus RK13 cells with the activity, there is a good chance that these new cell lines will not be capable of making C2 either. But, the point is well taken and there is a precedent for looking into compartmentalization effects for ectodomain proteases such as BACE 1. Some comments about protease identity are listed further below.

We have re-performed the biotinylation experiment from scratch to generate a new version of Figure E2. C2 clearly undergoes surface biotinylation in RK13 cells. We have added immunocytochemistry of permeabilized RK13 cells that show similar Golgi and cell surface staining for the WT, S1 and S3 PrP alleles. These data are now added to Figure E2 and the description on page 5 was adjusted to reflect these changes.

Cell type variations in C2 processing were noted in Fig. 2D. With respect to DNA construct differences there are no variables in promoter sequences or PrP 5' untranslated leader regions. The only variable is in the structure of the OR and we would contend that on a practical level overproduction of C2 fragment by the S3 allele in cells or in brain is a solid finding (we had five members of this lab do these studies).

Since the authors postulate a protease which is involved in the production of C2 PrP, application of protease inhibitors in RK13 cells might be interesting and reveal the nature of this protease.

This question does evolve naturally from our studies. The requirements for this type of experiment are a panel of class-specific inhibitors that can be without toxic side effects. We have begun to explore this question in some probe studies; to avoid complications that may arise from PrP fragments in stably expressing cells having a long half-life, we used "empty" RK13 cells acutely transfected with an S3PrP plasmid and then dosed with chemical inhibitors. The protein samples from lysed cells were PNGaseF-treated and then immunoblotted.

[an unpublished gel analysis presented to referees was removed from the letter at this point and references to specific chemical inhibitors are replaced below by the use of wwwww, xxxxx, yyyyy and zzzzz)

In these gels the top band is full-length PrP, the middle band is C2 and the lowest band is C1. Unexpectedly, although this initial inhibitor survey failed to define a "hit" with a strong effect on C2 production, C1 production (a de facto internal control) was affected by a wwwww protease inhibitor xxxxx - lane 6 (as well as by yyyyy protease inhibitor zzzzz, lane 9). This effect in plain sight diverges for the conventional view of C1 cleavage controlled by a zinc-containing disintegrin metalloprotease and will need to be dealt with. In addition, our preliminary mass spectrometry studies on brain material reveal points of C1 and C2 cleavage that differ from the conventional assignments (though showing some relationships to analyses by McDonald et al, JBC, 289:803-813, 2014).

Overall, while we can draw a provisional conclusion that C2 and C1 cleavage are going to be different enzymatically, our preliminary experiments in this area do not align with the mainstream literature and presenting them in isolation may cause confusion. We believe that a well validated answer to the question of the C2 protease identity will require further studies with inhibitor panels derived from synthetic chemistry, use of peptide and polypeptide protease inhibitors, precise mapping of the C2 cleavage sites in cells and in brain by immunoprecipitation and mass spectrometry, as well as cell biological and bioinformatics studies. For these reasons we believe the issue of protease identity is best left to a separate paper.

Finally, to address the molecular basis for the dissociation of prion titers and incubation time, the authors might want to analyze the underlying cell death mechanisms in more detail, although this probably is beyond the scope of this manuscript.

These mechanisms will ultimately be of great importance but we already have 27 display items so we are inclined to agree with the referee that these might be best reserved for a future study.

Apart from these mainly minor caveats, this is a technically very well done manuscript. This work is of excellent quality and is likely to have a great impact for future studies.

We appreciate these supportive comments.

Minor points:

1.) Providing the ratios of wt, C1 and C2 fragments for the in vitro data might be helpful, as done for the in vivo data in Fig. 3B.

Agreed. We have added a new panel to the right hand side of Fig. 3B. The distinction between S3 cells and WT PrP cells is p<0.0001. The trend is the same and corresponds to a slightly larger ratio of 4.2:1 (versus 3.5:1 for the brain). Text was adjusted accordingly on page 7.

2.) No blots without PNGaseF treatment are shown for the in vitro data. Are all PrPs indicative in their appearance of proper glycosilation?

For RK13 cells grown in vitro we have added the unglycosylated samples probed using the Sha31 antibody into Figure E2. This analysis is the same as on page 3 of this letter. There are no overt differences.

Glycosylated brain samples from healthy animals were presented in Fig 3A and PK-resistant glycosylated PrP from S1 and S3 mice is presented in Fig 5A.

3.) On page 8, second paragraph, an autocatalytic event is mentioned. This statement stands quite alone and might need some more explanation.

We have changed the text from

"...derives from an autocatalytic event mediated by the polypeptide chain."

to…

"…derives from a metal-assisted hydrolysis event mediated by the PrP polypeptide chain itself". The adjusted text is on page 8, second paragraph.

Referee #3 (Remarks):

The prion hypothesis was debated for many years but is generally accepted today and can be regarded as proven. The prion protein PrP in its pathogenic conformation PrSc is the infectious agent of prion diseases. That does not infer that the molecular mechanisms of infectivity and pathogenicity are known today in great detail. Since it is known that some principles of prions have general importance for protein misfolding diseases like Alzheimer, Parkinson etc. the importance of prion mechanism is much more general than anticipated for the so-called rare disease, i.e. the human prion disease Creutzfeldt-Jakob-disease. In the present manuscript Westaway et al. deal with a particular structure-function problem of PrP. PrP consists of a more or less globular C-terminal domain (aa90-aa231) and a flexible N-terminal domain. Whereas the (90-231)-domain is known since long as essential for infectivity and pathogenicity the function of the flexible N-terminus is not well known, but is exactly the subject of the present manuscript. In vivo cleavage leads to the fragments C1 and C2 in the linker region between the flexible and the globular region; the function of these fragments is not well understood. Five octarepeats (8 aa) and two hexarepeats (6 aa) in the N-terminus form binding sites for three or four copper-ions. The authors have constructed two groups of mutations, which either bind only one copper-ion (S3) with a compact structure or bind similarly to the wild type 3-4 copper-ions (S1) with a flexible structure.

Mechanisms of infectivity and pathogenicity of prions are of general interest in medicine; the detailed functional problems of the N-terminus are of interest mainly for prion-researchers. Therefore, the whole work is at the edge between general and special interest.

The authors apply a whole battery of methods: transgenetics, biochemical and biophysical analysis, cell biology test, in vivo tests etc.. The experimental effort is impressive and of high quality. The functional problem is not novel but the very detailed experimental approach is novel, and the complexity of the problem has never been dealt with before. Parameters like C1- and C2-expression, stability against GdnHCl denaturation, resistance/sensitivity for PK-proteolysis, spot counts in cell assays, incubation times and infectivity titers have been analysed. As far as possible the analysis was applied to the cellular form of PrP isolated from brain homogenate and to the pathogenic form PrPSc. Corresponding to the many methods also many results are presented. They are well presented in the figures, not always easy to follow by non-specialists.

We thank the referee for the supportive comments and we have been through the text to simplify/clarify some of the more technological aspects, as follows:

"PNGaseF treatment to remove glycans" – page 5

"We assessed this possibility for prions deriving from use of the S1 and S3.F88W allelic forms of PrP^C versus WT PrP^C employing PK digestion to examine changes in glycosylation profile or fragment size of PrP^{Sc}, as well as observing differences in susceptibility to PK digestion after exposure to increasing concentrations of the denaturant guanidine that may indicate a change in structure". – page 10

"SSCA; a technique to measure prion infectivity where a cell monolayer is infected with prions and eventually digested with PK to examine the number of cells with PrP^{Sc} and is in some respects similar to a plaque assay for viral particles" – page12

The results show quantitative differences, i.e. not yes-or-no-answers. Accordingly the discussion has to be somewhat vague and the conclusions show tendencies only. The referee can see two lines of conclusions: (i) Not the bound copper-ions are essential for the function of the N-terminus but the flexibility. This conclusion is more derived from the kind of aa-substitutions than from experimental facts: (ii) The N-terminus regulates more the pathology of the disease than the infectivity. It would be in line with the assumption that pathology and infectivity of prion diseases are uncoupled.

The somewhat weak conclusions appear inherent in the problem. The referee cannot recommend a clear-cut experiment to improve the strength of the conclusions. Also the function of the C1- and C2-fragments could not be clarified; for the sake of completeness the features of the proteolytic fragment PrP27-30 could be included into the discussion.

We have made several changes to the discussion with a view to improving clarity and the "yes/no" types of issues the referee is concerned about. The principle scientific finding is that changing the OR affects several endpoints; in other words, the effects of our mutations are pleiotropic. We suggest that our findings are compatible with PrP^C's OR serving as an extracellularly-displayed scaffold for a variety of interactors and that this role is modulated by chain flexibility. New text in the Discussion is as follows:

"In sum, the pleiotropic effects of proline substitutions that limit OR flexibility can be reconciled with this domain serving as a cell surface scaffold to bind diverse macromolecules and co-factors." See first paragraph, page 17

We deleted part of the last sentence of the Abstract (an inference about OR expansion alleles -- "...and is likely germane in familial prion disease where a single mutant OR produces multiple presentations within the same kindred.") to make the text simpler, more declarative. (Also see discussion about human prion disease with referee #1)

We have added new text to clarify that C2 PrP in an infection may not necessarily arise from endoproteolysis but from a processive protease activity often referred to as "N-terminal trimming" and attributed, at least in cells, to the action of lysosomal proteases (Taraboulos et al, Mol Biol Cell 3: 851-863, 1992). This new text is inserted towards the beginning of the Discussion and reads as follows:

*Lastly, by way of clarification, supraendogenous of C2 PrP are observed in prion infections (Chen et al., 1995; Yadavalli et al., 2004). In this context C2 PrP resembles the core of the infectious prion protein PrP27-30 that is PK-resistant, while C2 derived from PrP^C remains PK-sensitive (Mays et al., 2014)(**Table E1**). This type of C2 PrP may arise from a processive protease activity often referred to as "N-terminal trimming" and attributed, at least in cells, to the action of lysosomal proteases (Taraboulos et al., 1992). The Ca^{2+} activated non-lysosomal protease calpain has also been invoked for the production of this species (Yadavalli et al., 2004). Whether the ultimate effect of C2 PrP on infection differs depending on the mechanism of production remains to be determined. The new text is on page 14, last paragraph.*

In summary, a very extended, rigorous and detailed experimental work on a complex question. It is of relevance for our basic understanding of prion diseases on the level of molecular medicine. At present consequences for treatment or therapy of the disease cannot be drawn.

The high quality of the work deserves publication, the same is true in respect to the interest of the reviewer; the editor has to decide about the general interest of the journal.

We have made adjustments noted elsewhere in this letter to increase accessibility and hopefully also the general interest.

2nd Editorial Decision 18 December 2014

Thank you for the submission of your revised manuscript to EMBO Molecular Medicine. We have now received the enclosed report from the referee asked to re-assess it. As you will see this reviewer is now globally supportive and I am pleased to inform you that we will be able to accept your manuscript pending editorial final amendments.

Please submit your revised manuscript within two weeks. I look forward to seeing a revised form of your manuscript as soon as possible.

***** Reviewer's comments *****

Referee #1 (Remarks):

The revised version of the paper has dealt adequately with all concerns and points raised and I do not have any other comment to do on this manuscript.
